# TCC-GUI: a Shiny-based application for differential expression analysis of RNA-Seq count data

**DOI:** 10.1186/s13104-019-4179-2

**Published:** 2019-03-13

**Authors:** Wei Su, Jianqiang Sun, Kentaro Shimizu, Koji Kadota

**Affiliations:** 1 Graduate School of Agricultural and Life Sciences, The University of Tokyo, Yayoi 1-1-1, Bunkyo-ku, Tokyo, 113-8657 Japan; 2 Collaborative Research Institute for Innovative Microbiology, The University of Tokyo, Yayoi 1-1-1, Bunkyo-ku, Tokyo, 113-8657 Japan

**Keywords:** RNA-Seq, Bioinformatics, Differential expression analysis, Shiny app

## Abstract

**Objective:**

Differential expression (DE) is a fundamental step in the analysis of RNA-Seq count data. We had previously developed an R/Bioconductor package (called *TCC*) for this purpose. While this package has the unique feature of an in-built robust normalization method, its use has so far been limited to R users only. There is thus, a need for an alternative to DE analysis by *TCC* for non-R users.

**Results:**

Here, we present a graphical user interface for *TCC* (called TCC-GUI). Non-R users only need a web browser as the minimum requirement for its use (https://infinityloop.shinyapps.io/TCC-GUI/). TCC-GUI is implemented in R and encapsulated in Shiny application. It contains all the major functionalities of *TCC*, including DE pipelines with robust normalization and simulation data generation under various conditions. It also contains (i) tools for exploratory analysis, including a useful score termed average silhouette that measures the degree of separation of compared groups, (ii) visualization tools such as volcano plot and heatmap with hierarchical clustering, and (iii) a reporting tool using R Markdown. By virtue of the Shiny-based GUI framework, users can obtain results simply by mouse navigation. The source code for TCC-GUI is available at https://github.com/swsoyee/TCC-GUI under MIT license.

**Electronic supplementary material:**

The online version of this article (10.1186/s13104-019-4179-2) contains supplementary material, which is available to authorized users.

## Introduction

RNA-Seq is a common technique used to obtain gene expression data [1]. A major application of RNA-Seq data is to identify differentially expressed genes (DEGs) under different groups or conditions [2, 3]. Till date, many methods have been developed for this purpose [4–9], most of them implemented as R/Bioconductor packages [10, 11]. We had previously developed an R/Bioconductor package named *TCC* [7], the main characteristic of which is to implement a robust normalization procedure originally proposed by Kadota et al. [12]. It can provide accurate differential expression (DE) results especially when up- and down-regulated DEGs in one of the groups are extremely biased in their number. However, due to its limitations of usage by non-R users, there is a need of an alternative for DE analysis by *TCC*.

Here, we present a graphical user interface (GUI) for *TCC*, named TCC-GUI. Using the Shiny framework [13], it enables non-R users to manipulate the package and adjust parameters easily in order to view the DE results. The users only need a modern web browser as the minimal requirement. Contrary to the original *TCC* and like any other Shiny app, TCC-GUI provides plenty of visualization tools: principal component analysis (PCA) for exploratory analysis [14], Volcano plot [15] to view the DE results, and so on. While making figures with high customizability is not a trivial task even for experienced R users, TCC-GUI facilitates such a task in real-time.

## Main text

### Implementation

TCC-GUI is built in R. The current implementation depends on the following R packages: *TCC*, *shiny*, *shinyBS*, *shinycssloaders*, *shinydashboard*, *shinyWidgets*, *plotly*, *dplyr*, *DT*, *heatmaply*, *tidyr*, *utils*, *rmarkdown*, *data.table*, *RColorBrewer*, *knitr*, *cluster*, and *MASS*. While we primarily intend to use TCC-GUI via its URL [16] without installation for non-R users, it will launch locally from any R environment where prerequisite packages are installed. Although we have provided the source code in the additional file (Additional file 1), the latest version can also be downloaded through our GitHub page [17]. The latest version can be launched as follows:

$${\text{shiny}}::{\text{runGitHub}}\left( {{\text{``TCC}} - {\text{GUI''}},{\text{ ``swsoyee''}} {\text{ subdir}} = {\text{``TCC}} - {\text{GUI''}},{\text{ launch}} . {\text{browser}} = {\text{TRUE}}} \right)$$

### System

**Fig. 1:**
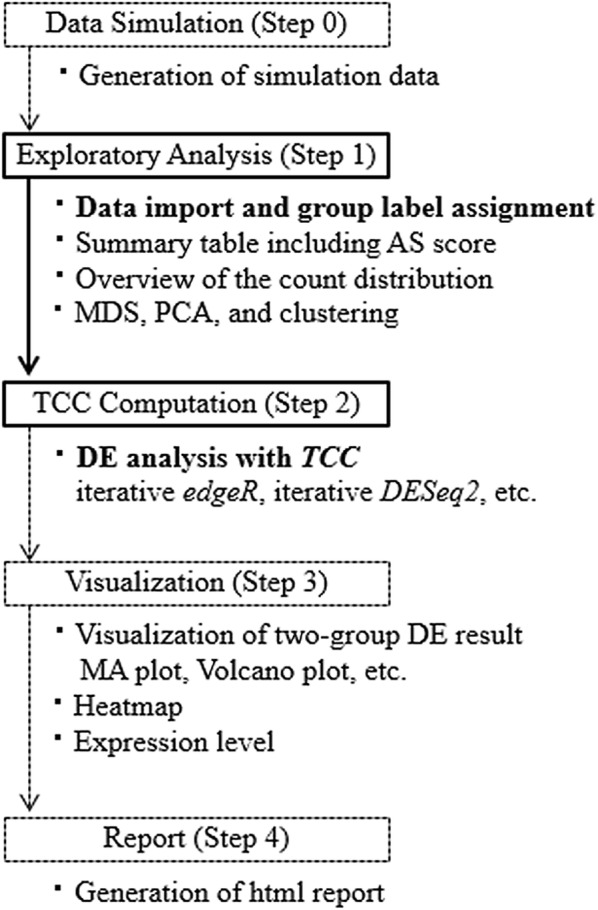
Analysis workflow and main functionalities in TCC-GUI. TCC-GUI provides a total of five steps (Steps 0–4), amongst which Steps 1 and 2 are mandatory

TCC-GUI provides a total of five steps for DE analysis (Fig. 1): data simulation (Step 0), exploratory analysis (Step 1), TCC computation (Step 2), visualization (Step 3), and report (Step 4). The minimum DE procedure only requires two steps (Steps 1 and 2). This is because Step 1 includes (i) data import and (ii) group label assignment as mandatory information for DE analysis. Step 2 is the core step of TCC-GUI application, where the DE analysis (i.e., statistical test), as implemented in *TCC*, is performed. The input is taken from Step 1 and DE result can be obtained in Step 2. Therefore, the remaining steps (Steps 0, 3, and 4) are not mandatory. While we will detail each step below, a tutorial for individual steps with appropriate screenshots is also provided in Additional file 2.

### Data simulation (Step 0)

Similar to the original *TCC*, TCC-GUI can generate simulation data with various conditions in Step 0. The generated data can, of course, be used as input for DE analysis within TCC-GUI, as well as other tools. The “hypoData” provided as sample dataset in Step 1 is essentially the same as that generated in Step 0 with almost default settings (except for the proportion of assigned DEGs in individual groups); the total number of genes was 10,000 (*N*_gene_ = 10,000), 20% of the genes were DEGs (*P*_DEG_ = 0.2), the number of groups was 2 (two-group comparison; G1 vs. G2), the levels of DE (fold-change; *FC*) for individual groups were fourfold (i.e., *FC*_G1_ = 4 and *FC*_G2_ = 4), the number of replicates (*NR*) were *NR*_G1_ = 3 and *NR*_G2_ = 3, and the proportions of assigned DEGs (*P*) were *P*_G1_ = 0.9 and *P*_G2_ = 0.1. Utilizing the advantage of GUI, users can recognize the number of DEGs assigned in individual groups in real-time. Simulation data for three-group comparison used in Tang et al. [18], for example, can be generated in Step 0.

### Exploratory analysis (Step 1)

**Fig. 2:**
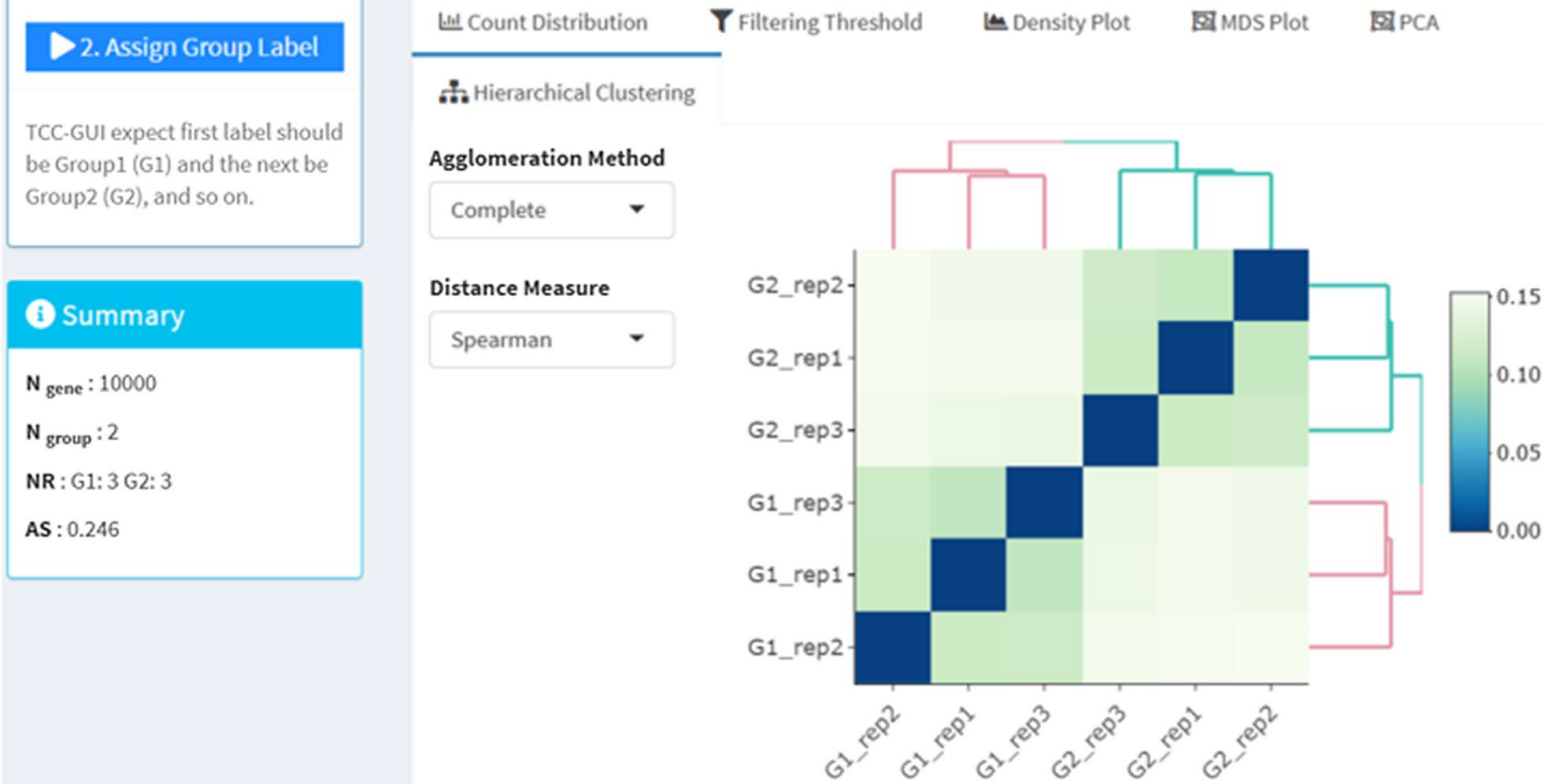
Exploratory analysis (Step 1). A dendrogram obtained from hierarchical sample clustering for a sample dataset “hypoData” is shown. The AS score (0.246) can be seen on the left side of this figure

At the first stage in Step 1, the user will be requested to import count data and assign group information for individual samples. The user can perform exploratory analysis (quality control) based on the group labels. The exploratory analysis includes count distribution, multi-dimensional scaling (MDS), PCA, hierarchical clustering, and so on. Figure 2 shows the dendrogram of hierarchical sample clustering (SC) for “hypoData”. There are two main clusters, each containing three samples belonging to the same group; this is reasonable because the data consists of two groups and contains 20% of DEGs (*P*_DEG_ = 0.2).

As a unique feature of TCC-GUI, it provides an average silhouette (AS) score for objectively estimating the degree of group separation [19, 20]. Silhouette was originally proposed for the interpretation and validation of cluster analysis [19]. Silhouettes provide a measure of how well a sample is classified when it is assigned to a cluster, based on both their tightness and the separation between them. Since the silhouette scores are calculated for individual samples, the AS value can be obtained by taking the mean across all samples.

We recently demonstrated that the AS value can be utilized for estimating the degree of group separation [20]. It ranges from − 1.0 to 1.0, and a higher AS value indicates a higher degree of group separation (i.e., a higher percentage of DEGs). In case of hypoData with *P*_DEG_ = 0.2, TCC-GUI outputs AS = 0.246 (see the left side of Fig. 2). For data that contain no or few DEGs (i.e., *P*_DEG_ = 0.0 approximately), the AS value would be around zero [20]. Although the AS values can be calculated independent of SC, they also provide a relevant measure for the degrees of separation between the groups of interest (e.g., G1 vs. G2) in SC results.

### TCC Computation (Step 2)

This step includes data normalization and DEG identification. It provides several analysis pipelines that can be performed by changing options in the parameter setting panel (see Step 2 in Additional file 2), They include the iterative *edgeR* pipeline (as default), iterative *DESeq* *2* pipeline, and the original *edgeR* or *DESeq* *2* pipeline.

The DE results will appear in the “Result Table” panel after the operation ends. While the main output is a *p*-value that indicates the degree of DE between the compared groups, other information, such as adjusted *p*-values (i.e., *q*-values) and log ratio (M) values, are also provided. The user can download the complete DE results and TCC-normalized data as CSV files. The user can also extract any subset of genes by the column of interest in the table. This can be done by utilizing the boxes at the bottom of the table. For example, the user will see a range of log-ratios (= log_2_(G2/G1)) as [− 6.63, 6.48] in the box of the “M Value” column. The user can extract genes that are twofold higher in G2 than in G1 by setting the range appropriately, as in [1.0, 7.0].

TCC-GUI also provides R codes internally used to execute the *TCC*. Researchers can learn the functions that are used internally, utilize this code as a template, and obtain reproducible results.

### Visualization (Step 3)

**Fig. 3:**
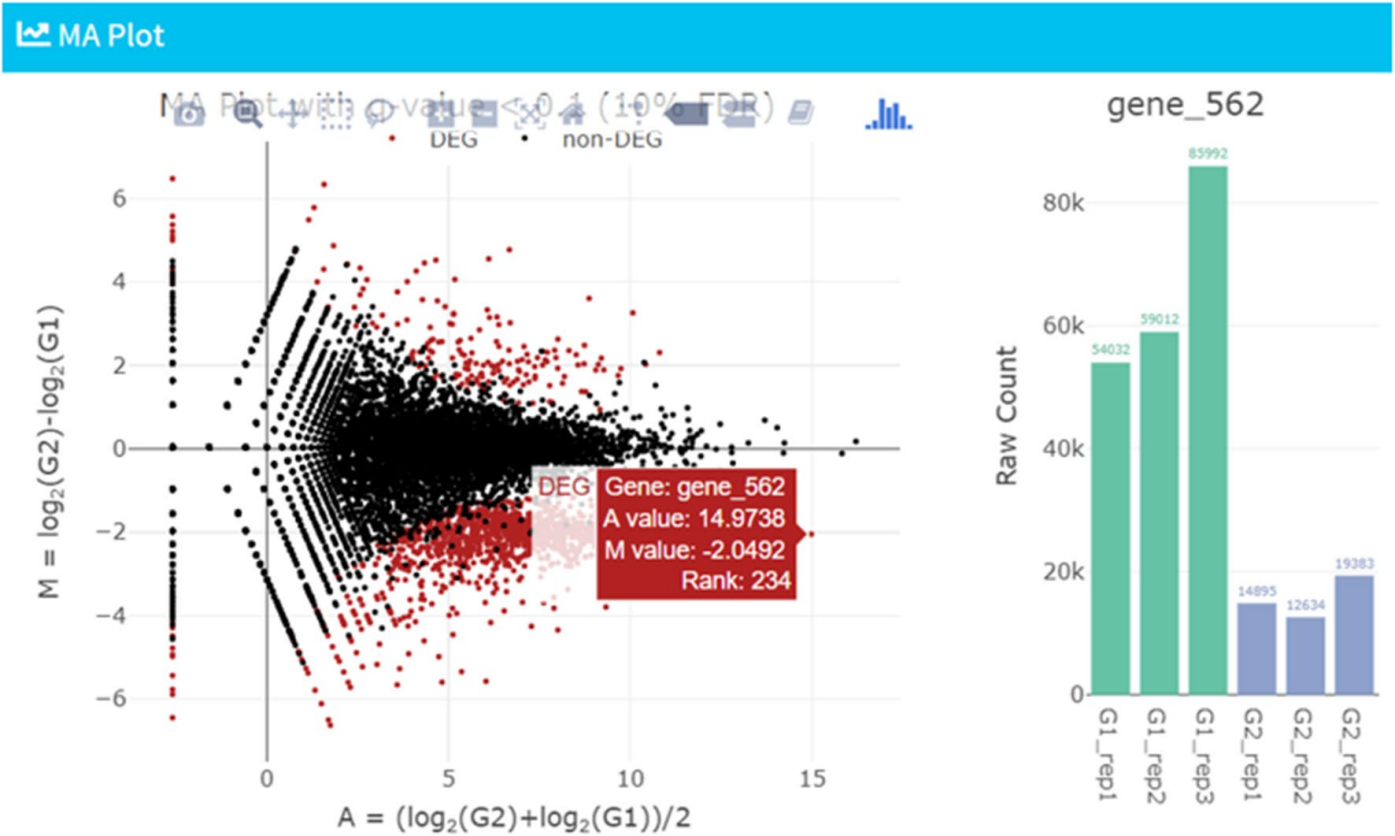
Visualization (Step 3). As a representative visualization of DE result, the MA plot for two-group comparison, using “hypoData”, is shown. By hovering data point (i.e., gene_562) of interest in the scatter plot, the expression pattern across samples can be seen on the right side

The MA plot is commonly used to visualize the DE result of two-group comparisons, by transforming the data onto log-ratio (M) scale as Y-axis and average expression (A) scale as X-axis (Fig. 3). While 1320 genes, satisfying 10% false discovery rate (FDR), are colored in dark red by default, both the cut-off value and color may be changed by the user (see Step 3 in Additional file 2). Owing to the interactive GUI framework, the user can obtain more detailed information about a gene of interest (e.g., “gene_562”) by placing the cursor in that location (see the right side of Fig. 3).

Based on the definition of FDR, the 1320 genes satisfying 10% FDR are theoretically composed of 1320 × 0.1 = 132 non-DEGs while the remaining 1320 × 0.9 = 1188 are *true* DEGs. Similarly, 1841 genes satisfying 40% FDR are composed of 1841 × 0.4 = 736.4 non-DEGs and 1841 × 0.6 = 1104.6 *true* DEGs. Although genes satisfying arbitrarily defined FDR cut-offs are usually defined as DEGs, an increase in the number of genes by loosening the FDR cut-off does not necessarily indicate an increase in the number of *true* DEGs [20]. In TCC-GUI, the number of genes satisfying various FDR thresholds can be interactively seen in the “MA Plot Parameters” panel. In addition, information of FDR cut-offs in increments of 0.05 is also provided in tabular form. This information, as well as the AS value, would be helpful to estimate how the *true* DEGs are included in the input data.

TCC-GUI provides Volcano plot as another way to visualize the DE result of two-group data. In contrast to MA plots that are constructed by plotting the M values (Y-axis) vs. A values (X-axis), it plots M values on the X-axis and statistical significances as − log_10_(*p*-value) on the Y-axis. Many users will be interested in genes located in the upper left or upper right areas in the plot. The user can, of course, change the colors and cut-off values for both axes and see the number of genes satisfying both the cut-offs. In case of hypoData with default settings, the user will see 1374 genes down-regulated and 283 genes up-regulated in G2. This is quite reasonable because the hypoData contains 1800 genes down-regulated and 200 genes up-regulated in G2.

TCC-GUI also provides two other visualization tools for somewhat general purposes: “Heatmap” and “Expression Level”. “Heatmap” is a graphical representation of data where the individual count values contained in a matrix are represented as pseudo-colors. Hierarchical clustering is usually performed on heatmap, enabling users to interpret the overall picture of expression patterns with ease [21]. “Expression Level” can be used to visualize expression patterns for genes of interest. It would be useful to visualize, for example, expression patterns of top-ranked DEGs obtained from two- or more-group comparison.

### Report (Step 4)

Although TCC-GUI has the option to export results after every step, some users may prefer the output merged into one single file. Like other sophisticated GUI-based tools, such as PIVOT [22], TCC-GUI supports this functionality in the final Step 4.

### Representative analysis of a real count dataset

Here, we demonstrate a representative analysis of a real count dataset [23], available at the ReCount website [24]. The dataset consisted of 36,536 genes × 21 liver samples. Bottomly et al. [23] had studied the expression levels of two common inbred mouse strains used in neuroscience research, i.e., 10 C57BL/6J strains and 11 DBA/2 J strains. TCC-GUI displayed the results of two-group comparison: (i) the AS value was 0.187 in Step 1, (ii) 22,604 low-count genes were filtered (i.e., 36,536-22,604 = 13,932 genes were used as input for TCC computation in Step 2), and (iii) 1530 genes satisfying 10% FDR (i.e., *P*_DEG_ = 10.98%) were detected as DEGs after TCC computation in Step 2. These values were exactly the same as those described in Zhao et al. [20]. A series of screenshots for this analysis is given in Additional file 3.

## Conclusion

TCC-GUI is a browser-based application for DE analysis of RNA-Seq data. It enables non-R users to perform the *TCC* package without installation. In addition to the functionalities originally implemented in *TCC*, TCC-GUI provides plenty of interactive visualization functions. The powerful in-built functions would also be satisfactory for experienced R users.

## Limitations

While the development is complete from the end-user perspective, the internally used R codes are still cluttered. Moreover, the GUI in Step 4 is still in need of further improvement. These refinements are desirable in near future.

## Additional files

**Additional file 1.** Source code for TCC-GUI. This file can be used to launch TCC-GUI locally. The primary aim is to provide reproducible results described in the manuscript.

**Additional file 2.** Tutorial for TCC-GUI. A step-by-step instruction to perform individual steps for TCC-GUI is provided.

**Additional file 3.** Representative analysis of Bottomly’s dataset. A series of screenshots while analyzing Bottomly’s real count dataset is provided.

## Abbreviations

A: average expression
AS: average silhouette
DE: differential expression
DEG: differentially expressed gene
FC: fold-changes
FDR: false discovery rate
GUI: graphical user interface
M: log ratio
MDS: multi-dimensional scaling
*N*_gene_: total number of genes
*NR*: number of replicates
PCA: principal component analysis
SC: sample clustering
TCC: Tag Count Comparison
