# Supplementary material for: TCC-GUI: a Shiny-based application for differential expression analysis of RNA-Seq count data
Source: BMC Res Notes. 2019 Mar 13;12:133. doi: 10.1186/s13104-019-4179-2 (PMC6417217; doi:10.1186/s13104-019-4179-2)
Supplement: Supplementary file 2 — Additional file 2. Tutorial for TCC-GUI. A step-by-step instruction to perform individual steps for TCC-GUI is provided. [file 13104_2019_4179_MOESM2_ESM.pptx]

## Slide 1
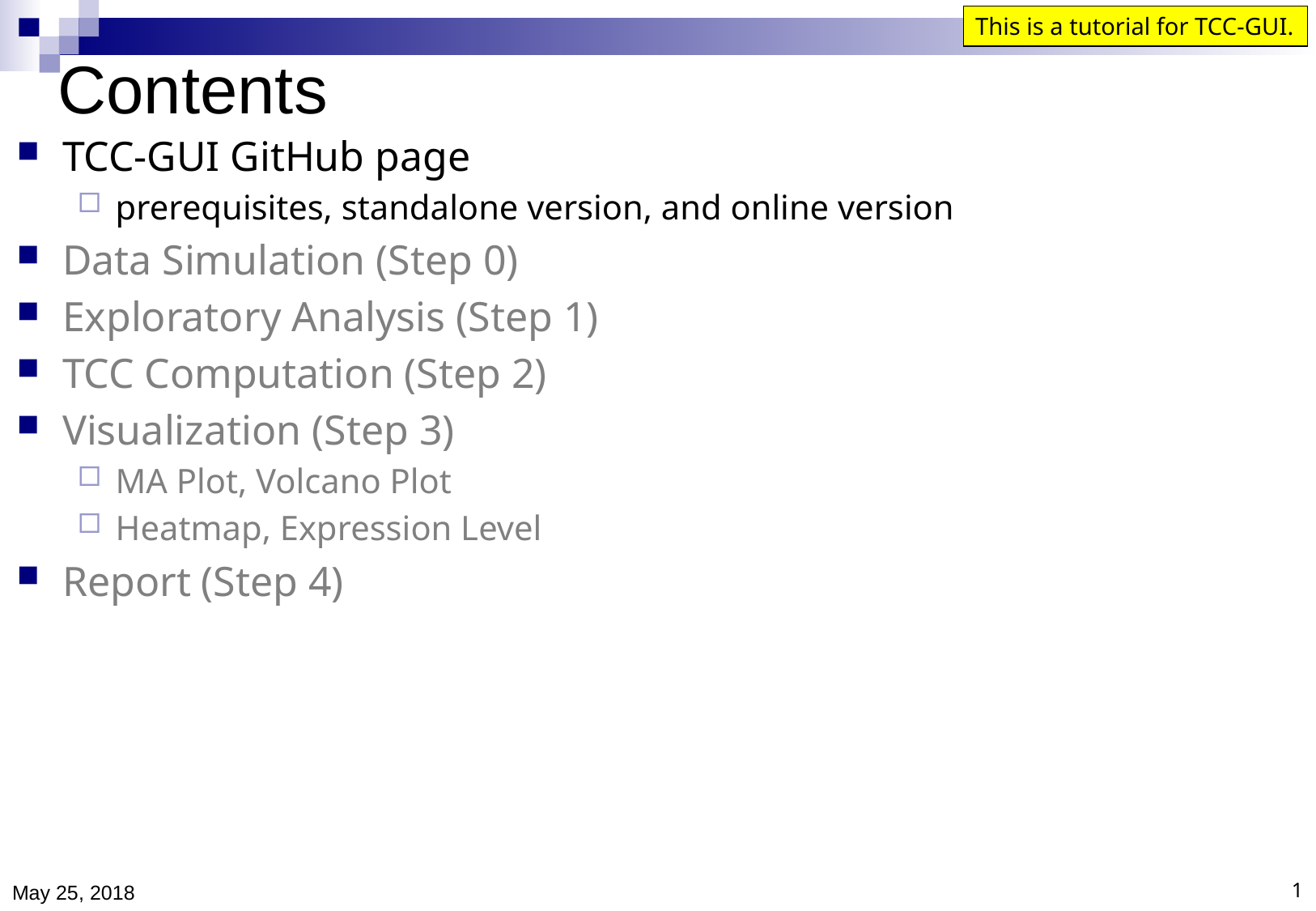

This is a tutorial for TCC-GUI.
# Contents
TCC-GUI GitHub page
prerequisites, standalone version, and online version
Data Simulation (Step 0)
Exploratory Analysis (Step 1)
TCC Computation (Step 2)
Visualization (Step 3)
MA Plot, Volcano Plot
Heatmap, Expression Level
Report (Step 4)
May 25, 2018
1

## Slide 2
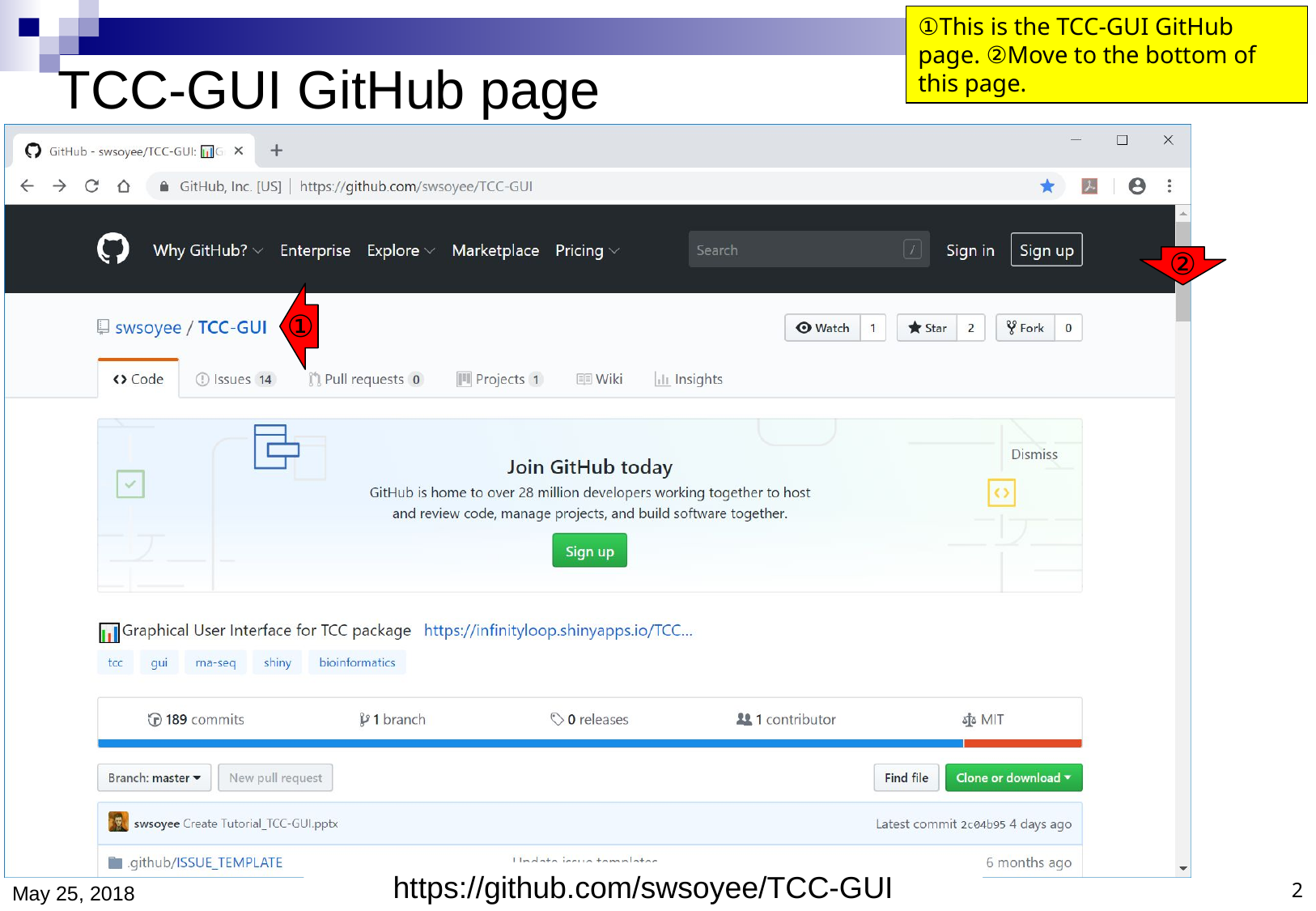

①This is the TCC-GUI GitHub page. ②Move to the bottom of this page.
# TCC-GUI GitHub page
②
①
May 25, 2018
2
https://github.com/swsoyee/TCC-GUI

## Slide 3
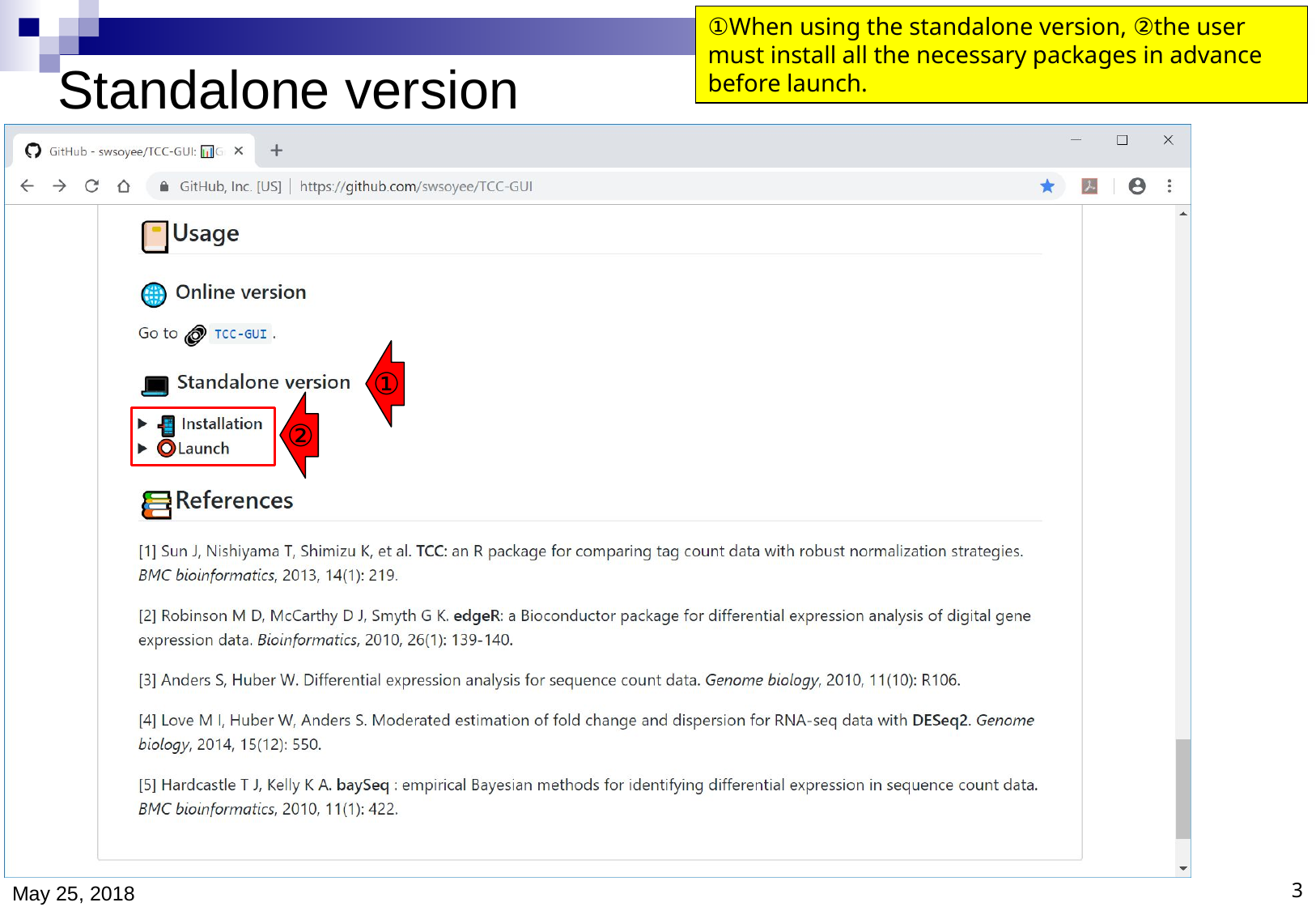

①When using the standalone version, ②the user must install all the necessary packages in advance before launch.
# Standalone version
①
②
May 25, 2018
3

## Slide 4
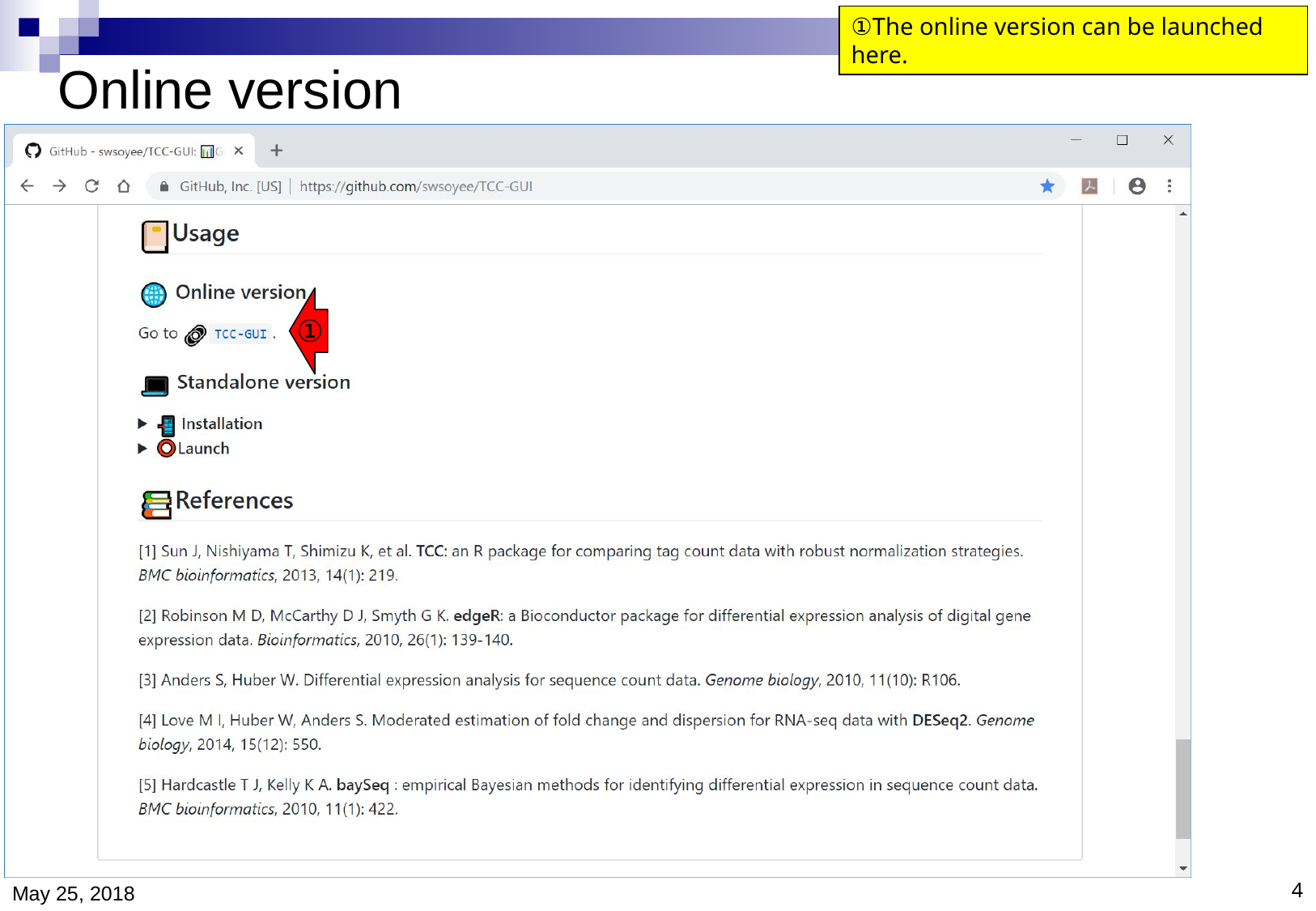

①The online version can be launched here.
# Online version
①
May 25, 2018
4

## Slide 5
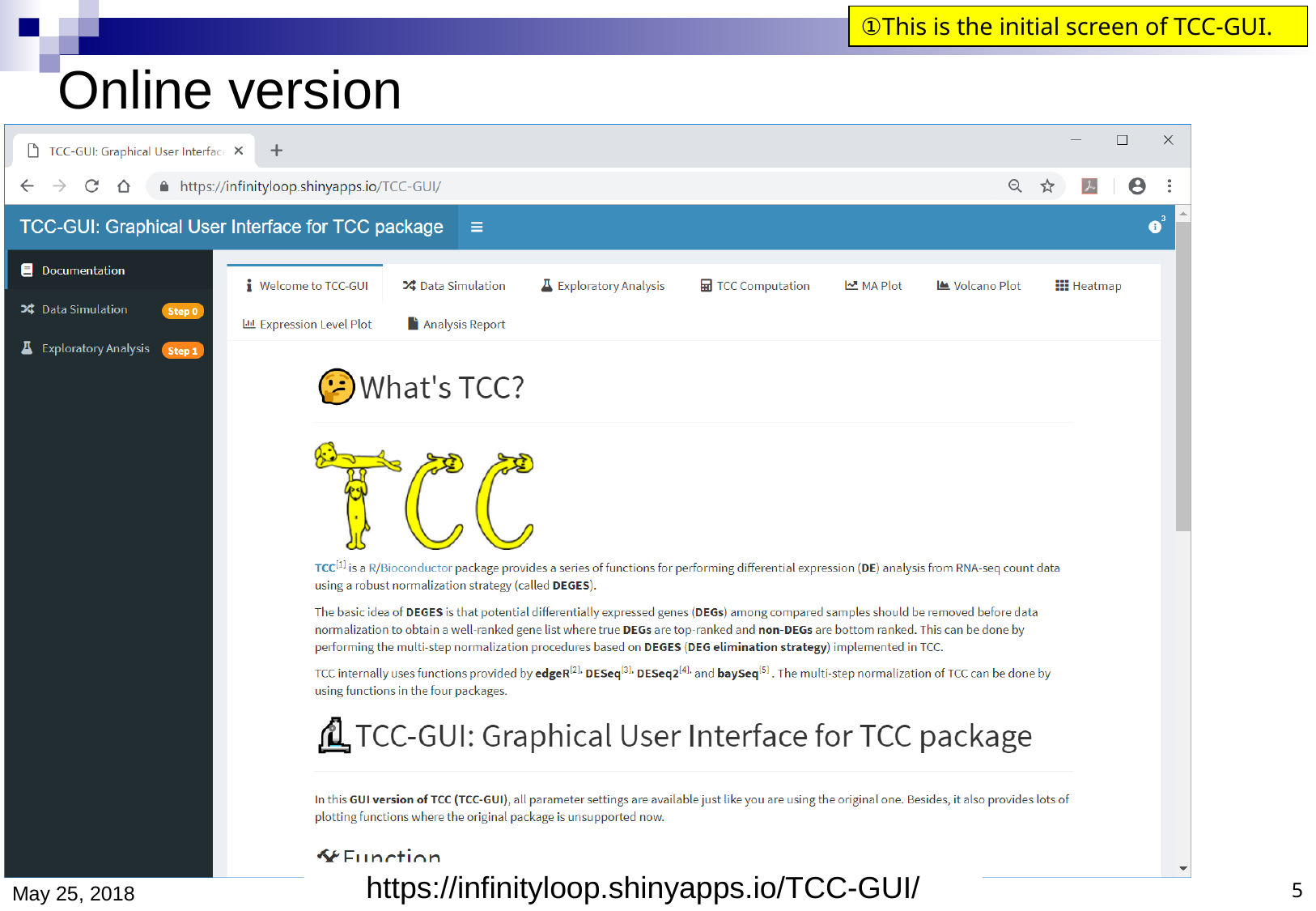

①This is the initial screen of TCC-GUI.
# Online version
May 25, 2018
5
https://infinityloop.shinyapps.io/TCC-GUI/

## Slide 6
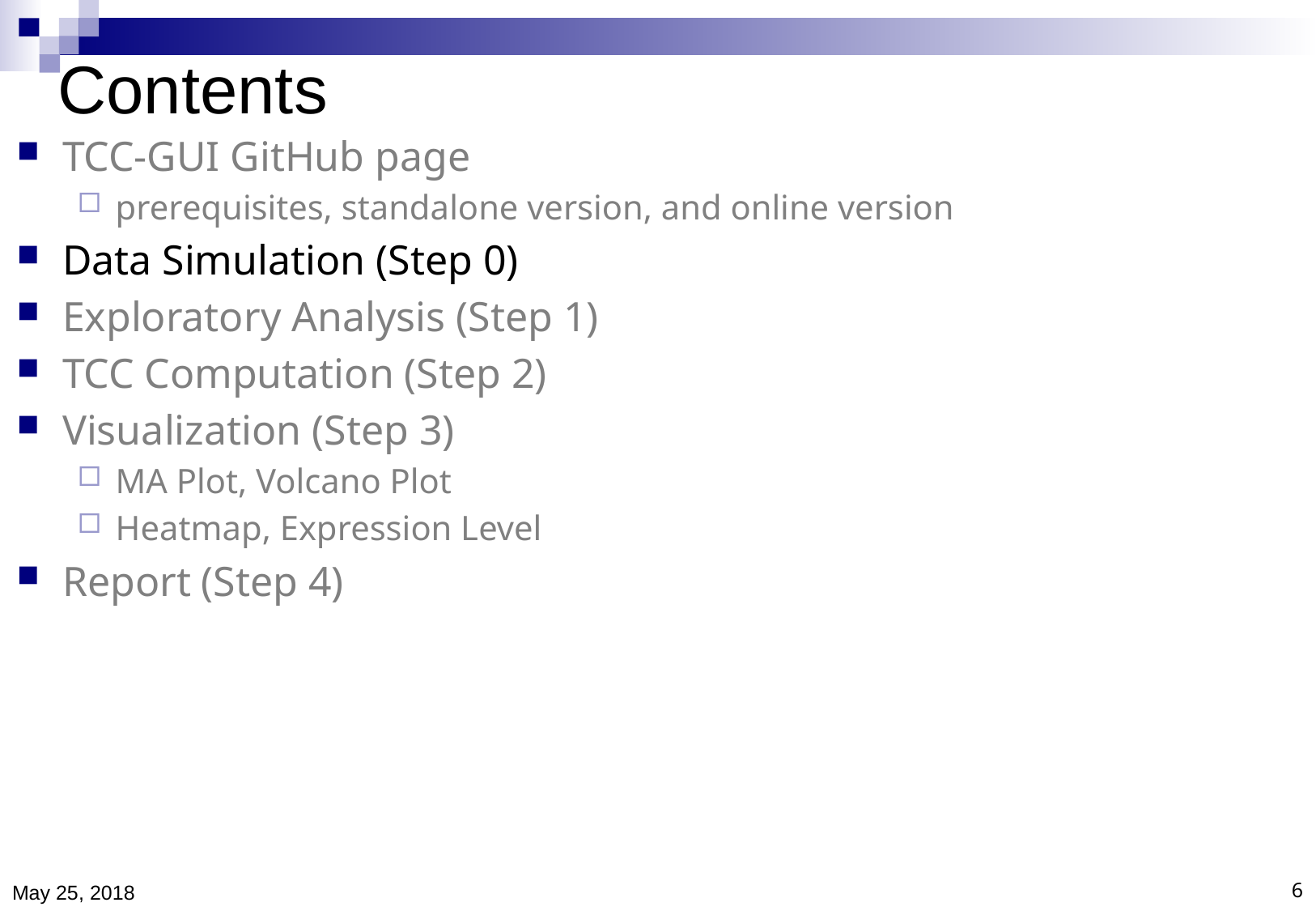

# Contents
TCC-GUI GitHub page
prerequisites, standalone version, and online version
Data Simulation (Step 0)
Exploratory Analysis (Step 1)
TCC Computation (Step 2)
Visualization (Step 3)
MA Plot, Volcano Plot
Heatmap, Expression Level
Report (Step 4)
May 25, 2018
6

## Slide 7
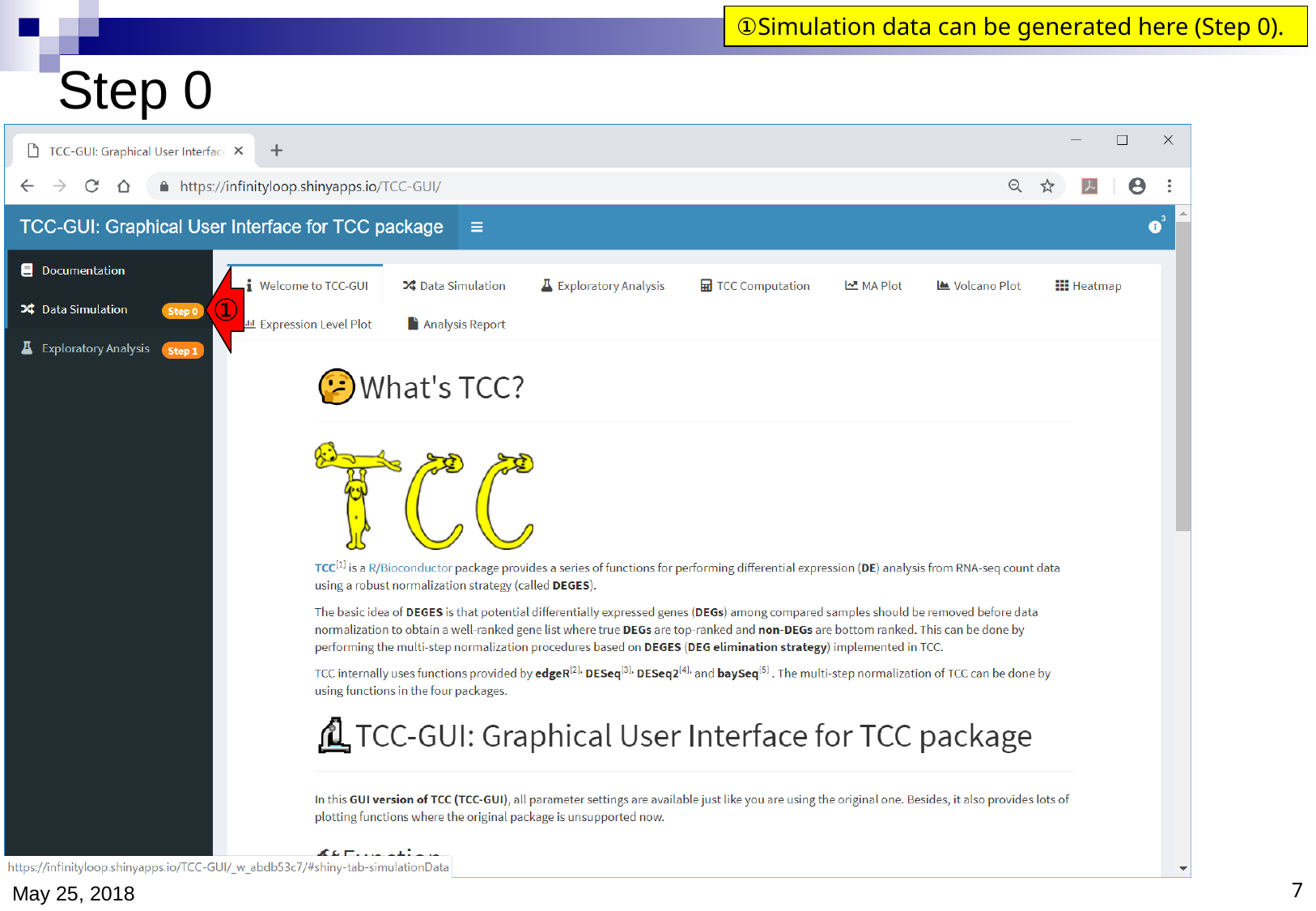

①Simulation data can be generated here (Step 0).
# Step 0
①
May 25, 2018
7

## Slide 8
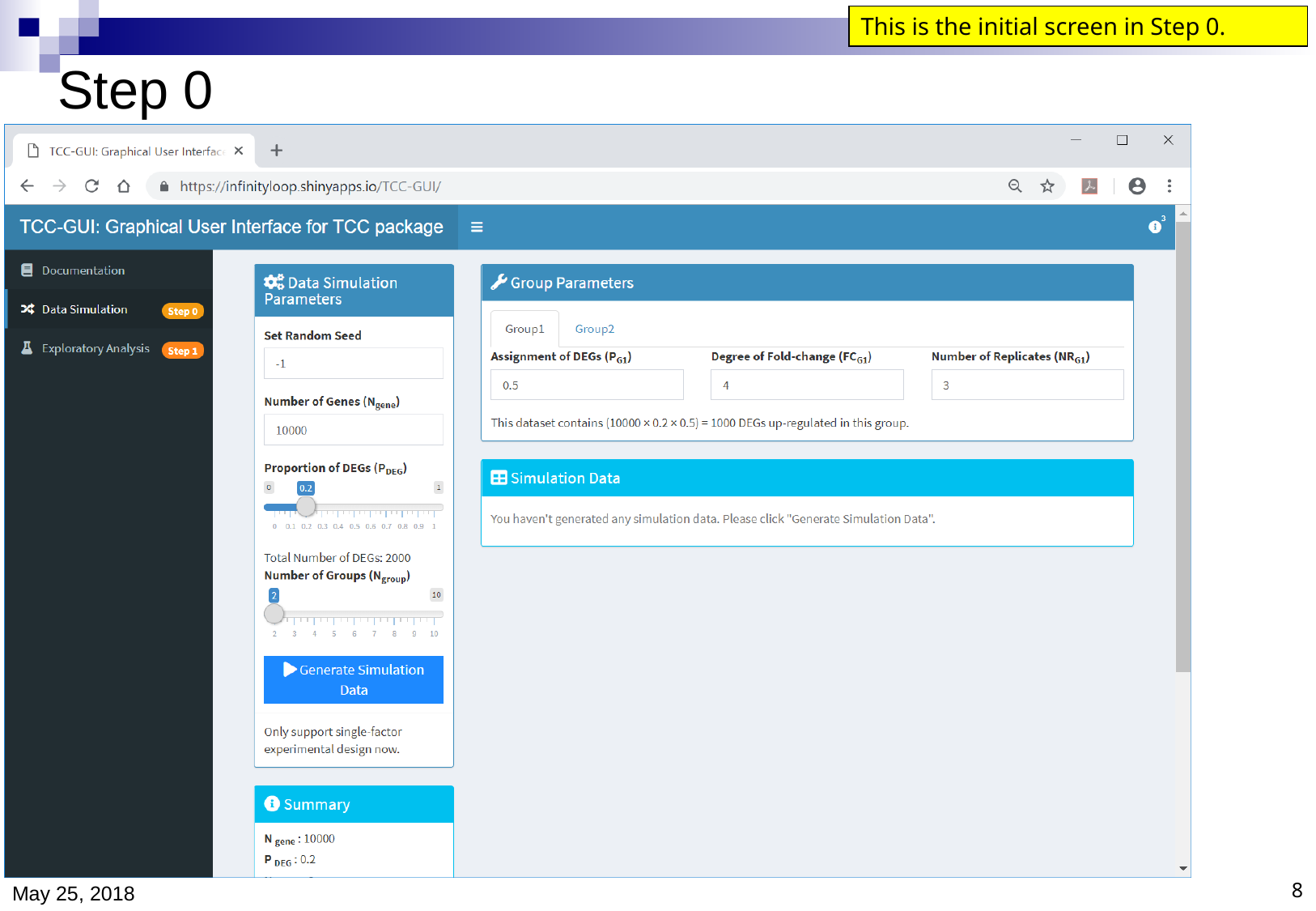

This is the initial screen in Step 0.
# Step 0
May 25, 2018
8

## Slide 9
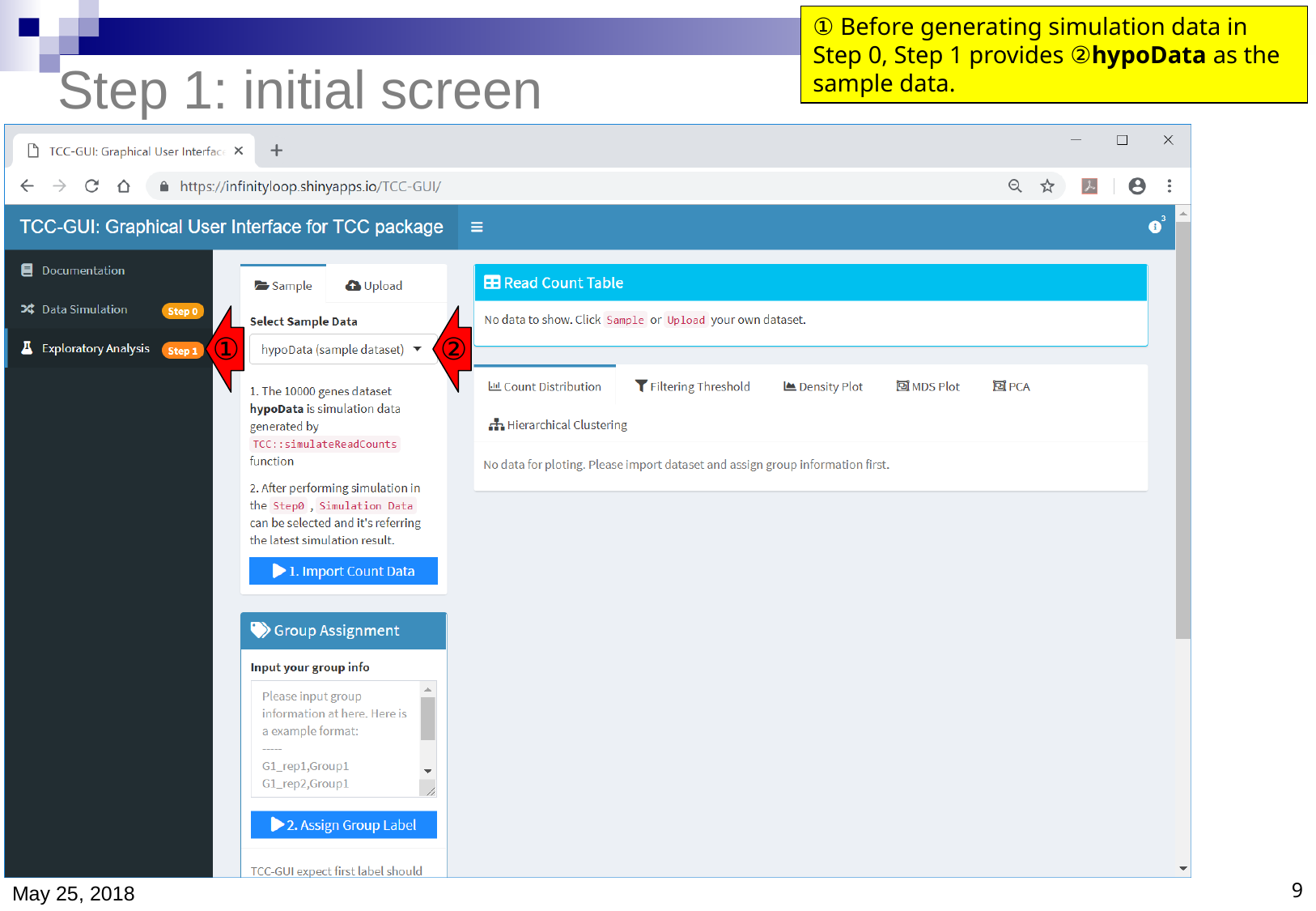

① Before generating simulation data in Step 0, Step 1 provides ②hypoData as the sample data.
# Step 1: initial screen
①
②
May 25, 2018
9

## Slide 10
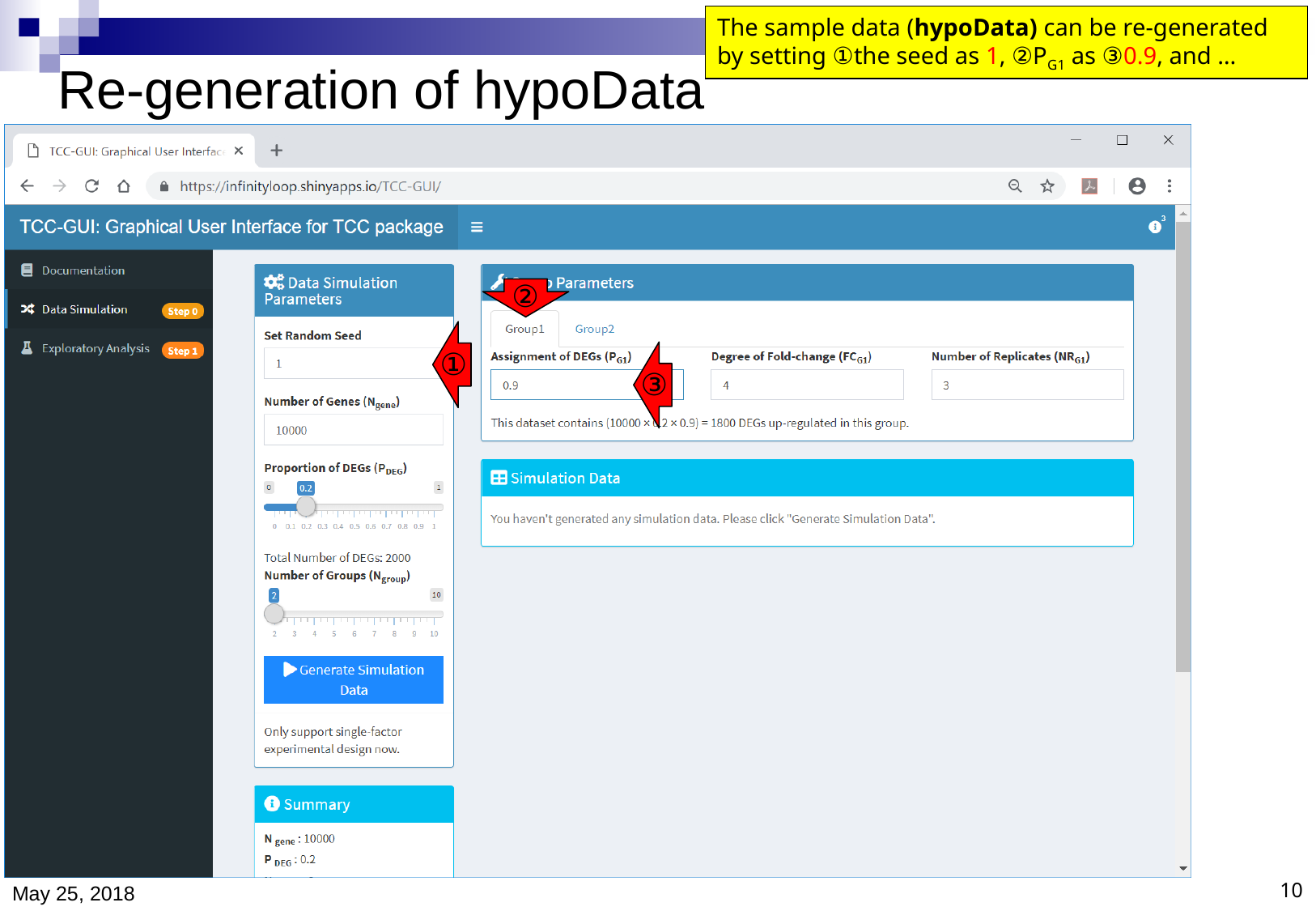

The sample data (hypoData) can be re-generated by setting ①the seed as 1, ②PG1 as ③0.9, and …
# Re-generation of hypoData
②
①
③
May 25, 2018
10

## Slide 11
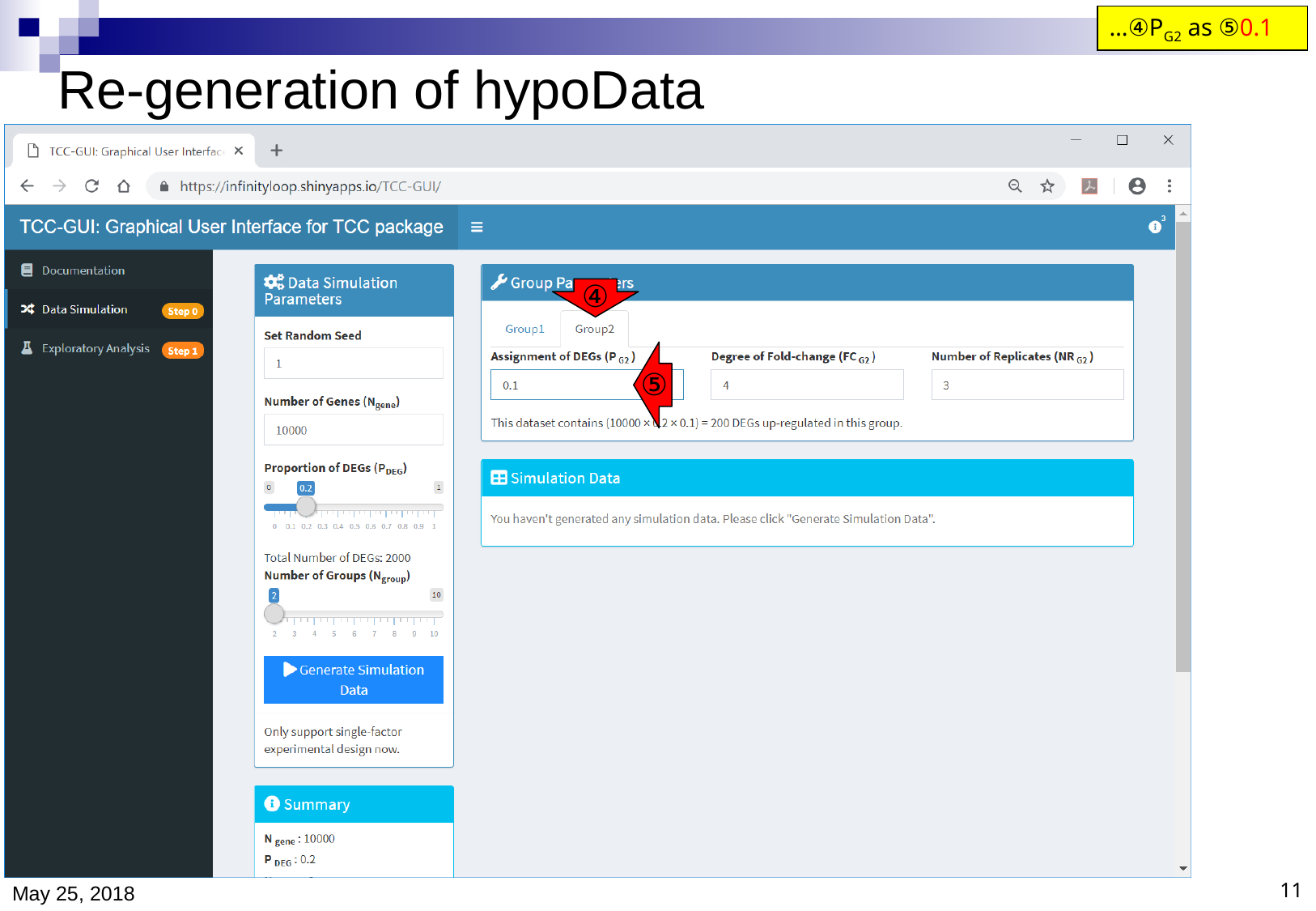

…④PG2 as ⑤0.1
# Re-generation of hypoData
④
⑤
May 25, 2018
11

## Slide 12
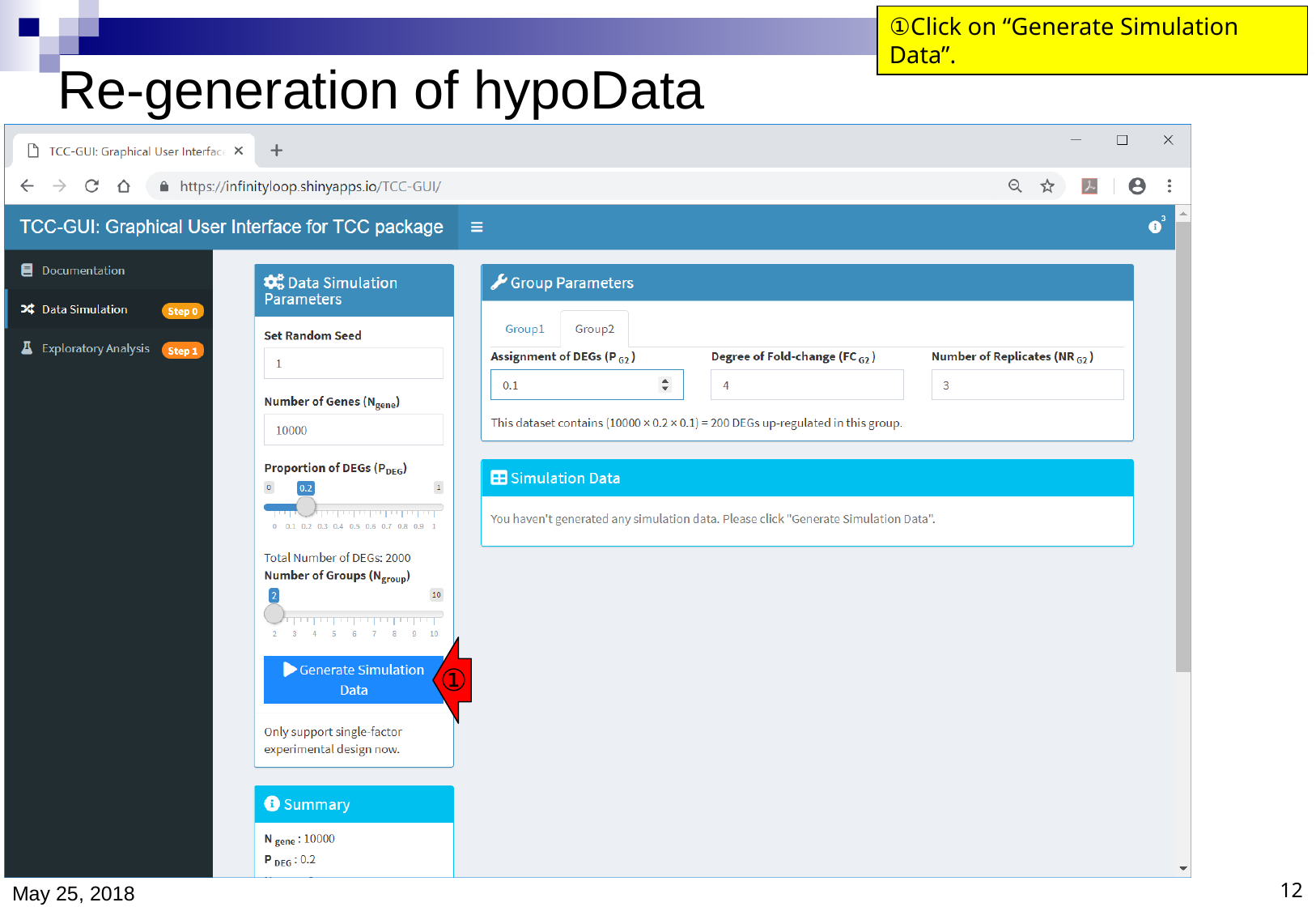

①Click on “Generate Simulation Data”.
# Re-generation of hypoData
①
May 25, 2018
12

## Slide 13
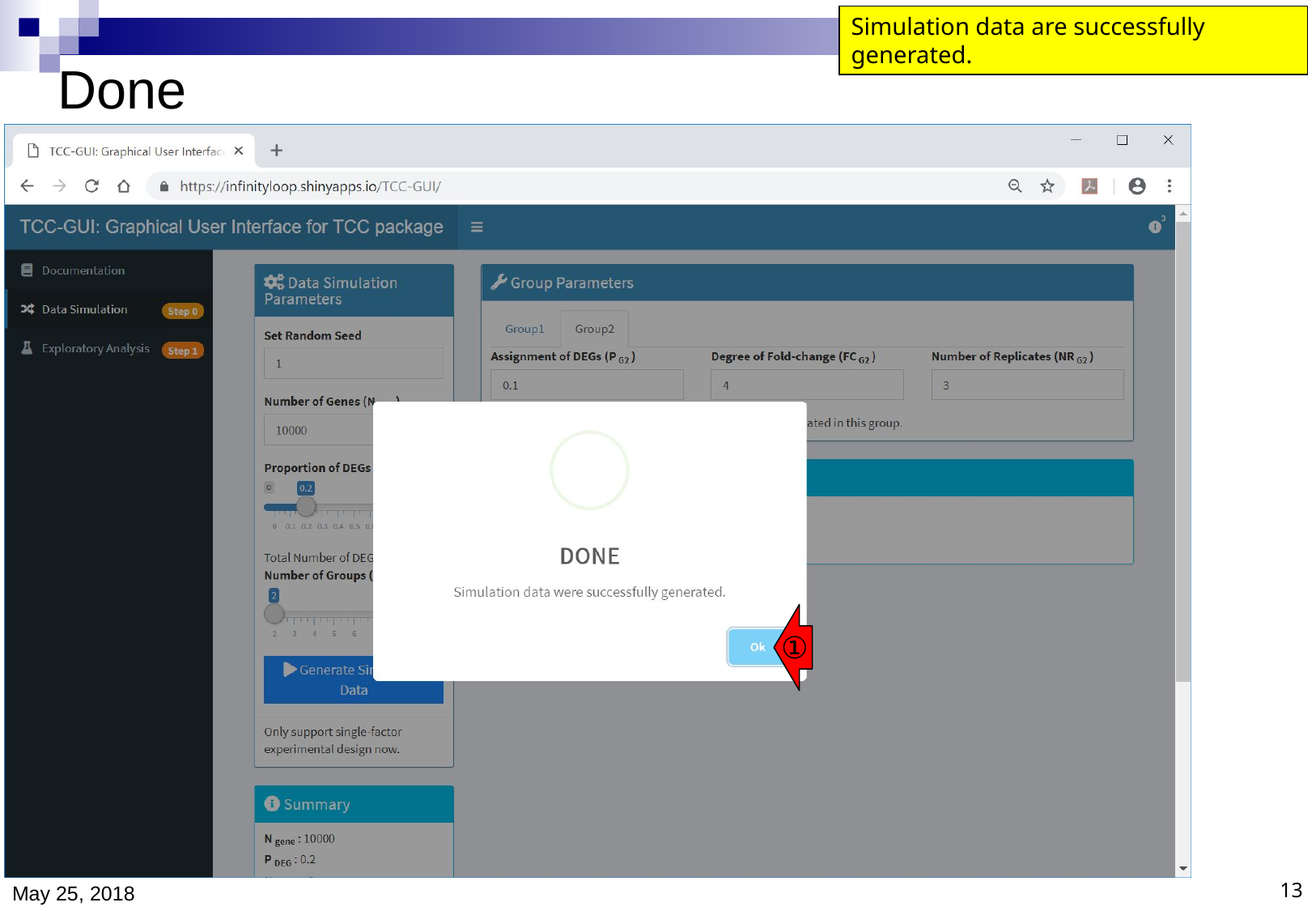

Simulation data are successfully generated.
# Done
①
May 25, 2018
13

## Slide 14
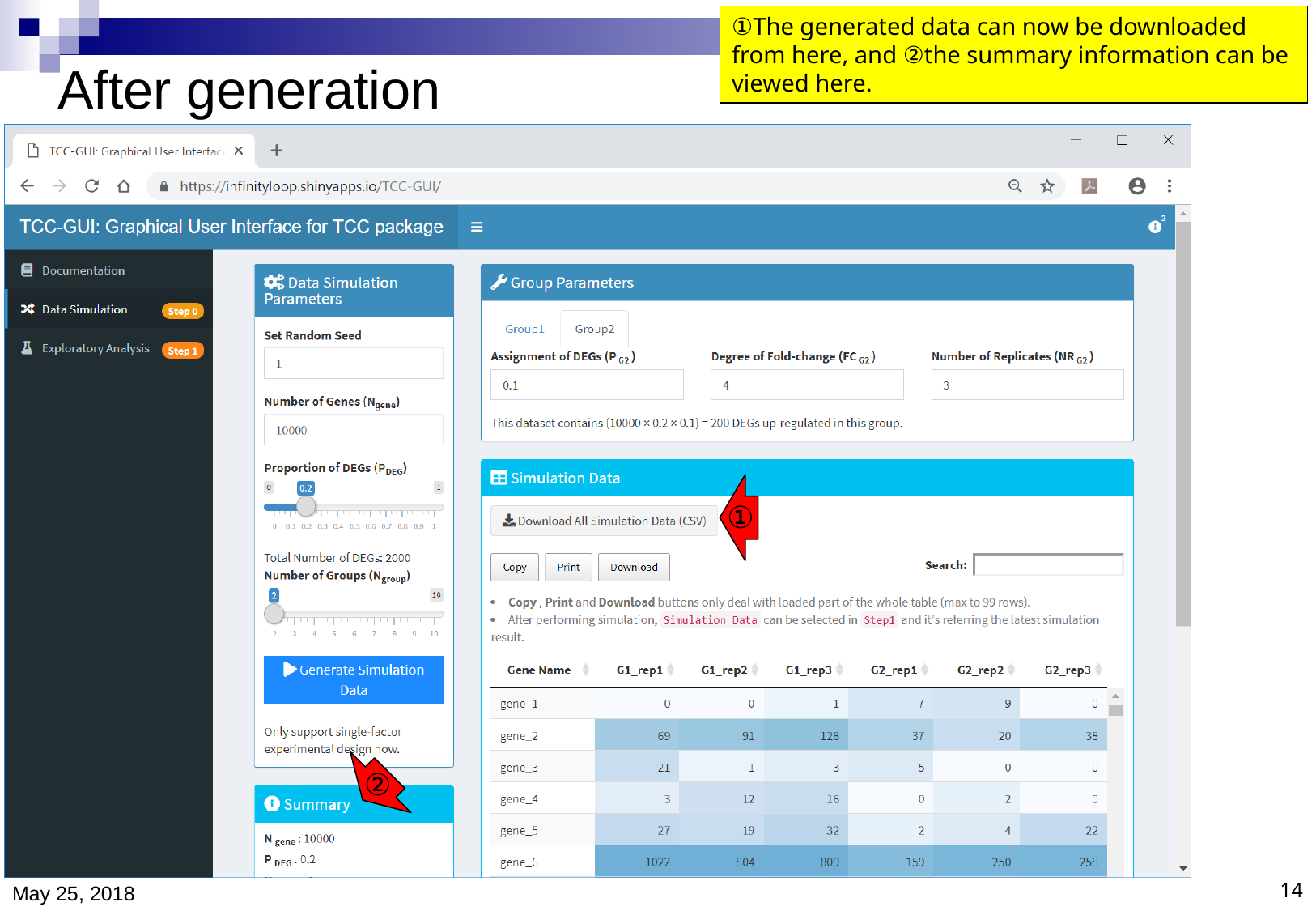

①The generated data can now be downloaded from here, and ②the summary information can be viewed here.
# After generation
①
②
May 25, 2018
14

## Slide 15
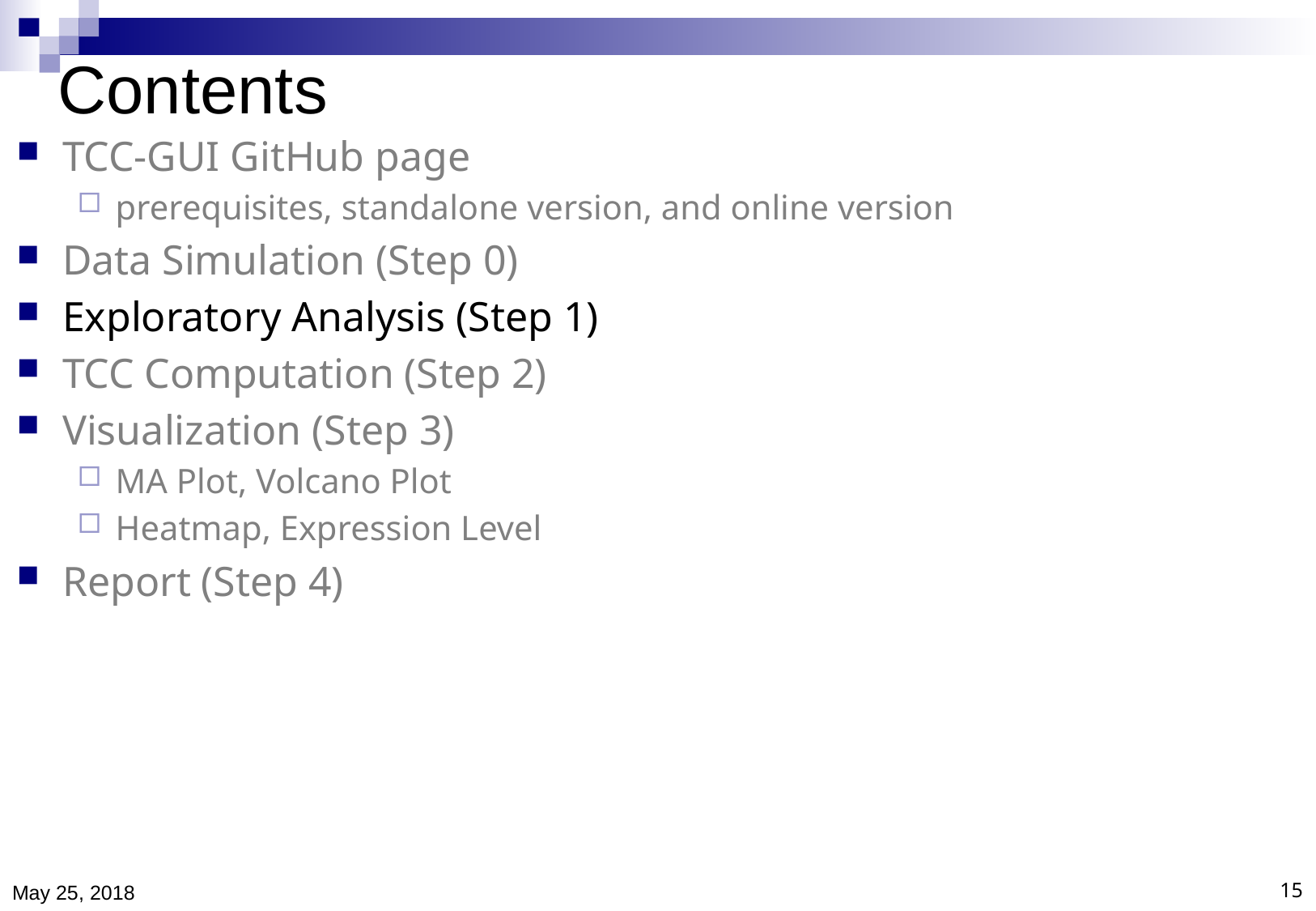

# Contents
TCC-GUI GitHub page
prerequisites, standalone version, and online version
Data Simulation (Step 0)
Exploratory Analysis (Step 1)
TCC Computation (Step 2)
Visualization (Step 3)
MA Plot, Volcano Plot
Heatmap, Expression Level
Report (Step 4)
May 25, 2018
15

## Slide 16
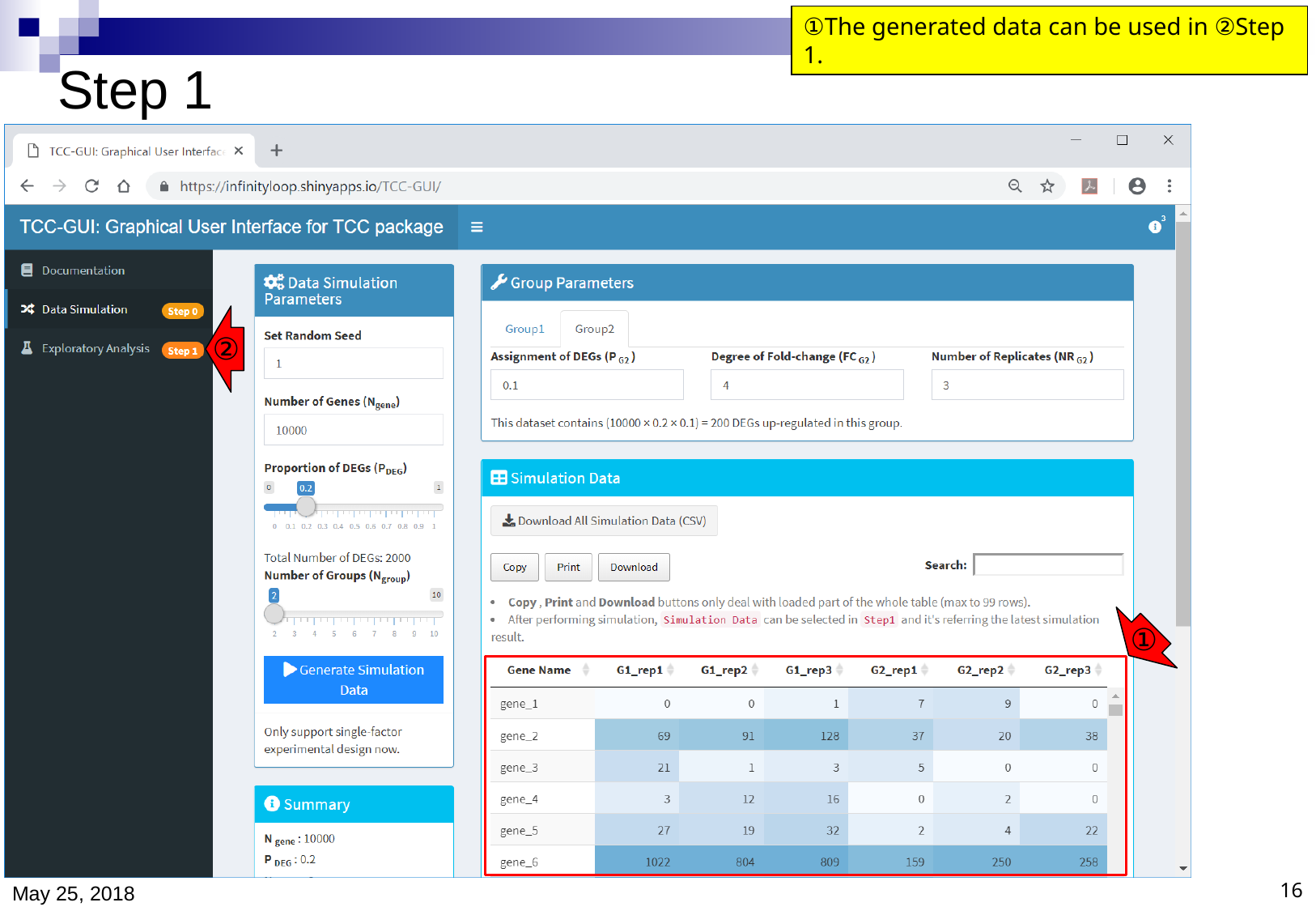

①The generated data can be used in ②Step 1.
# Step 1
②
①
May 25, 2018
16

## Slide 17
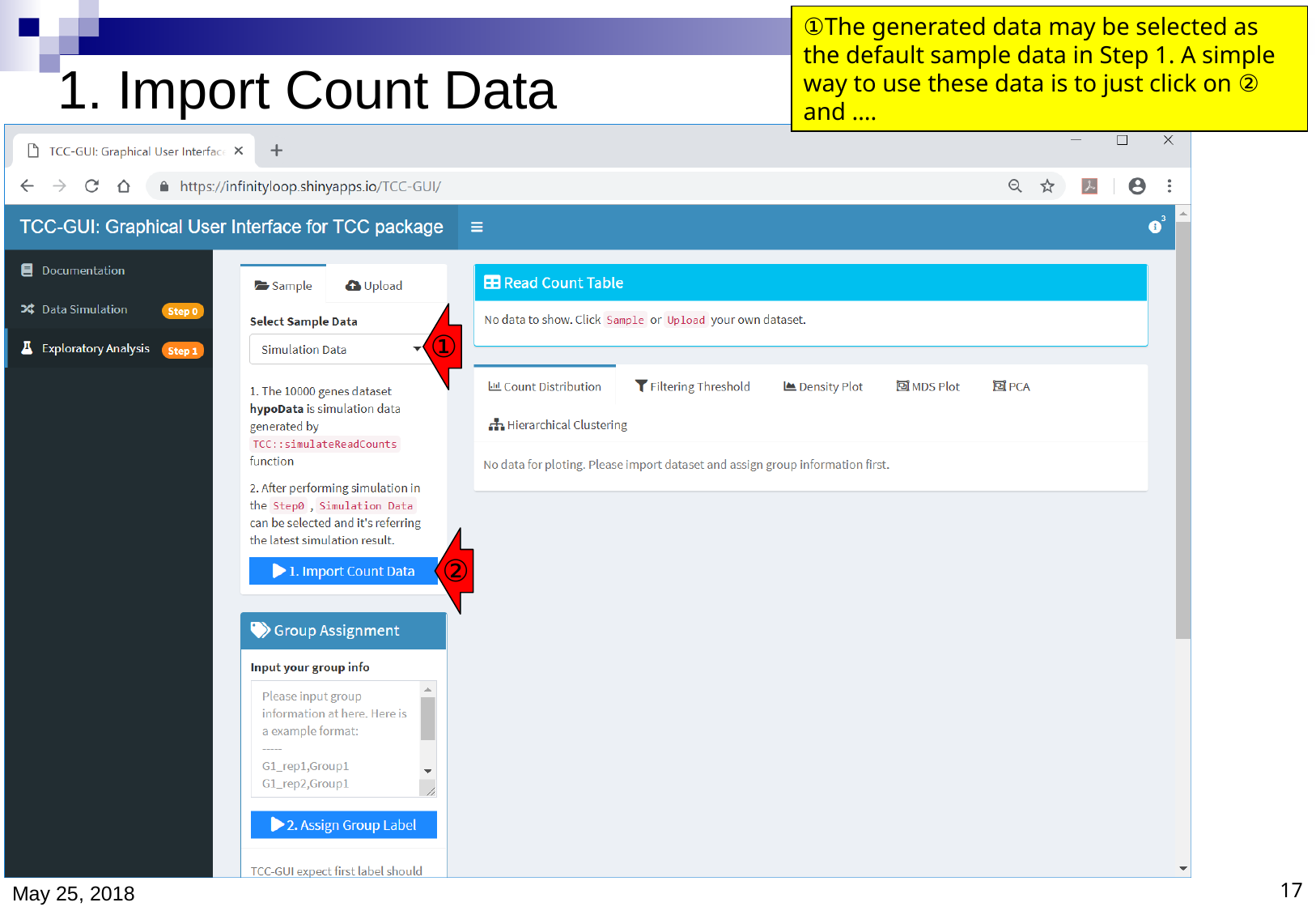

①The generated data may be selected as the default sample data in Step 1. A simple way to use these data is to just click on ② and ….
# 1. Import Count Data
①
②
May 25, 2018
17

## Slide 18
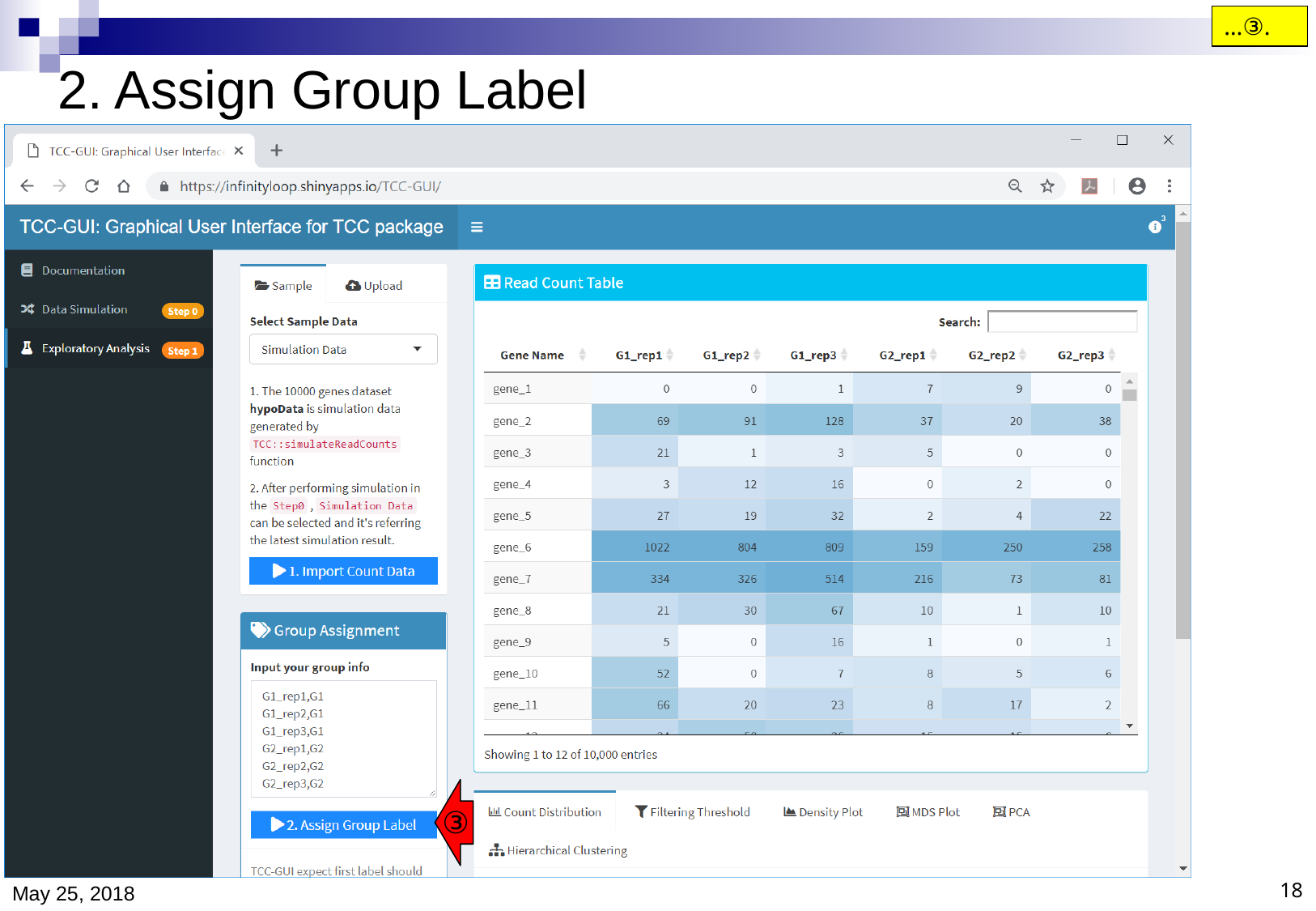

…③.
# 2. Assign Group Label
③
May 25, 2018
18

## Slide 19
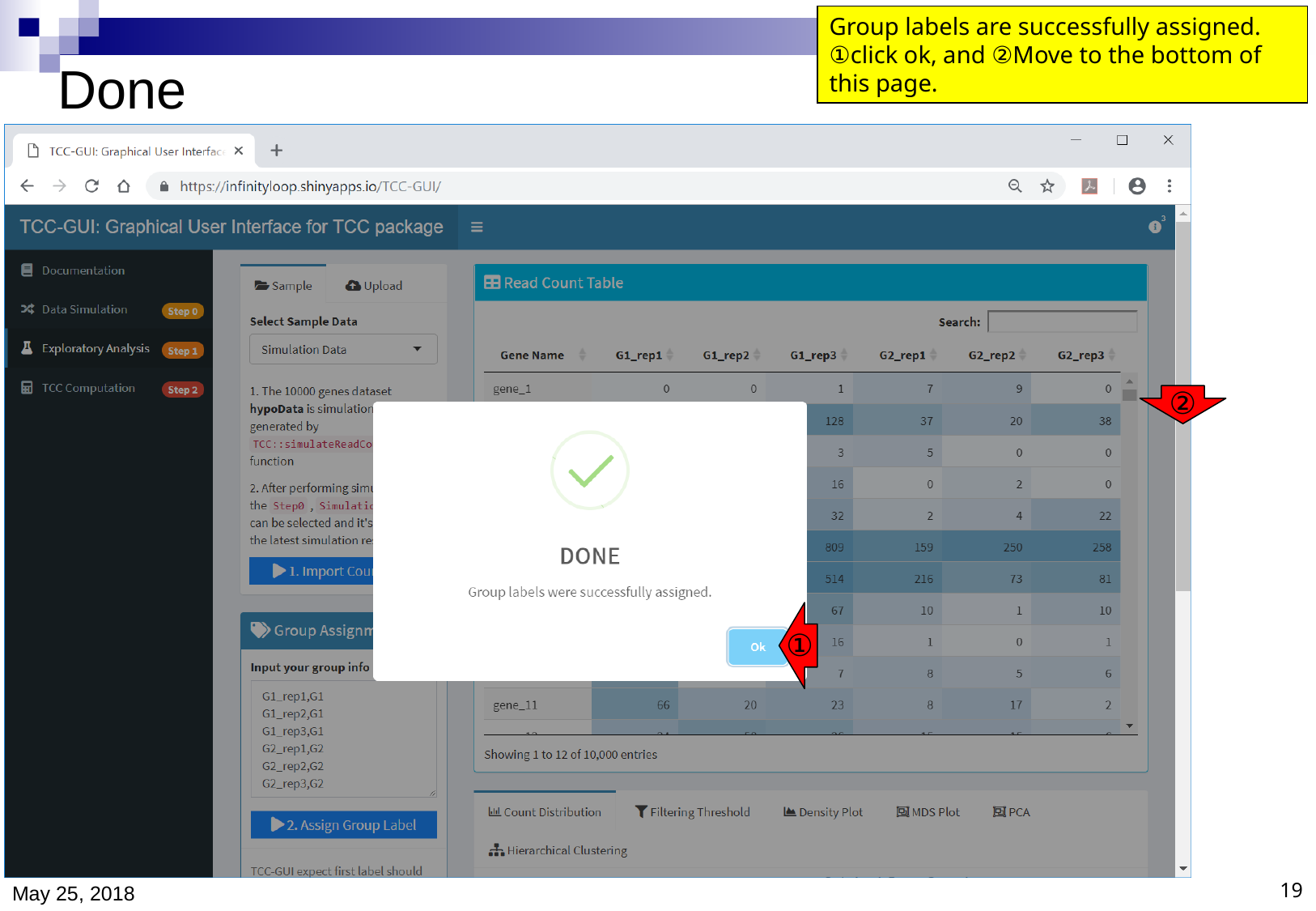

Group labels are successfully assigned. ①click ok, and ②Move to the bottom of this page.
# Done
②
①
May 25, 2018
19

## Slide 20
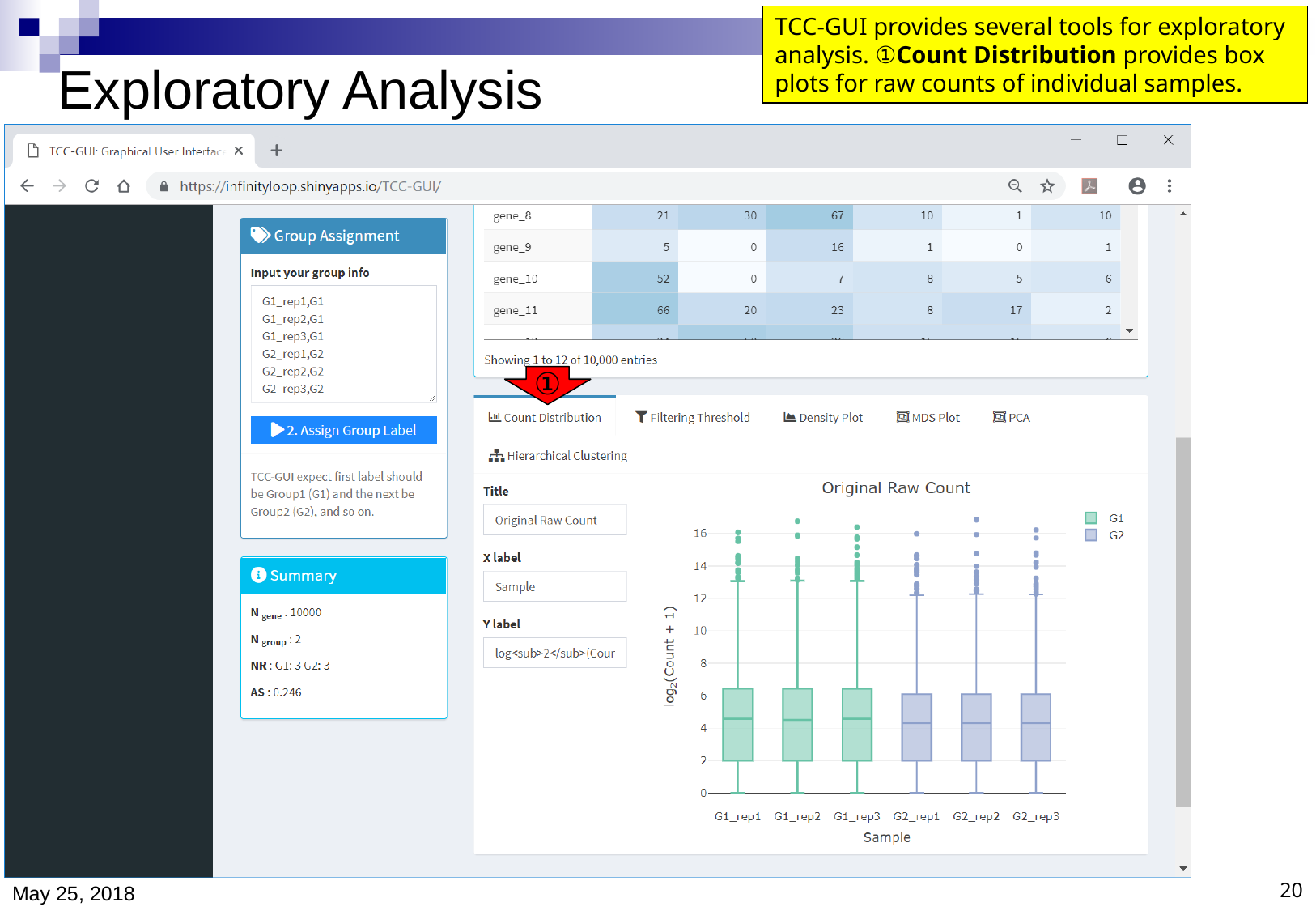

TCC-GUI provides several tools for exploratory analysis. ①Count Distribution provides box plots for raw counts of individual samples.
# Exploratory Analysis
①
May 25, 2018
20

## Slide 21
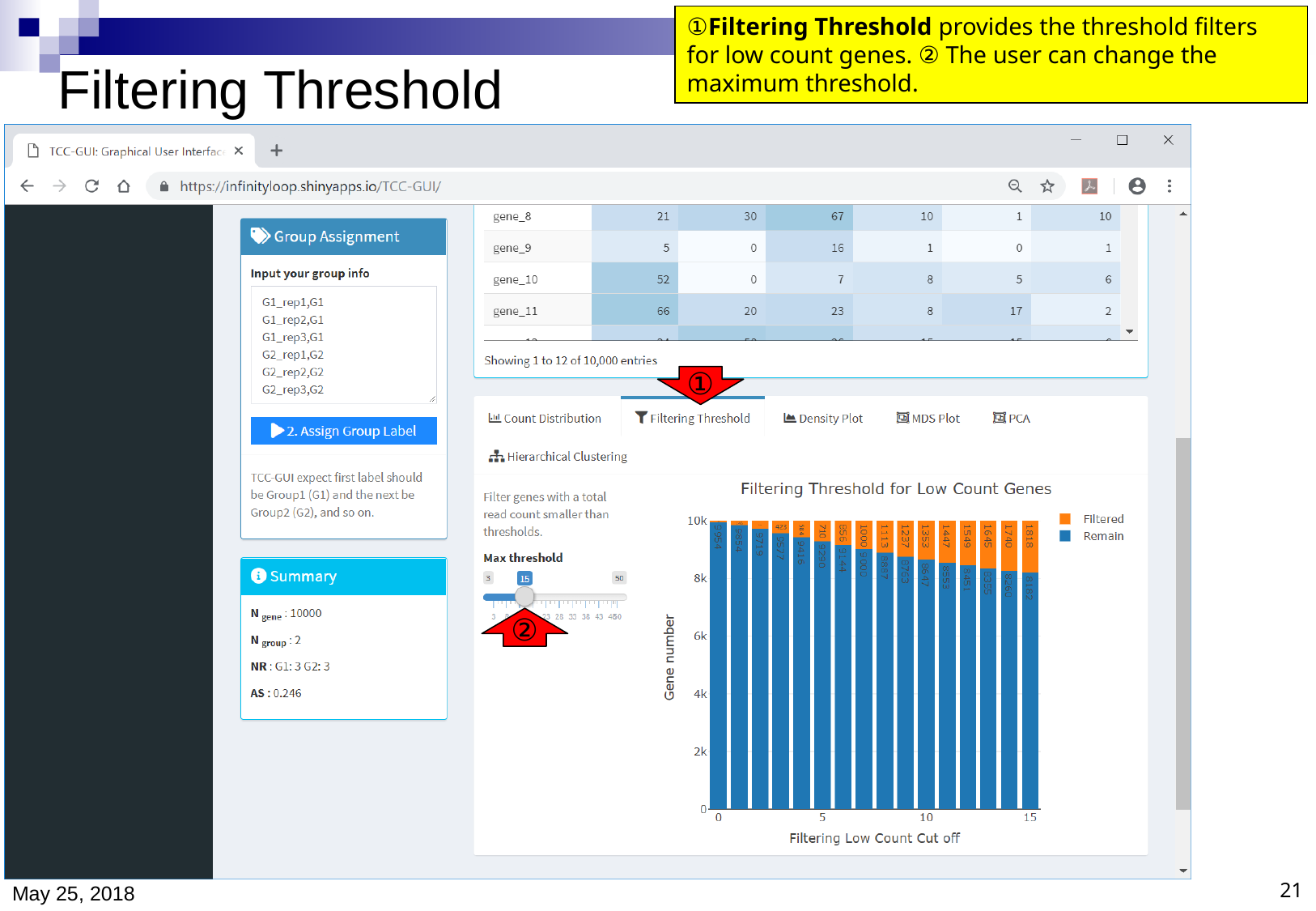

①Filtering Threshold provides the threshold filters for low count genes. ② The user can change the maximum threshold.
# Filtering Threshold
①
②
May 25, 2018
21

## Slide 22
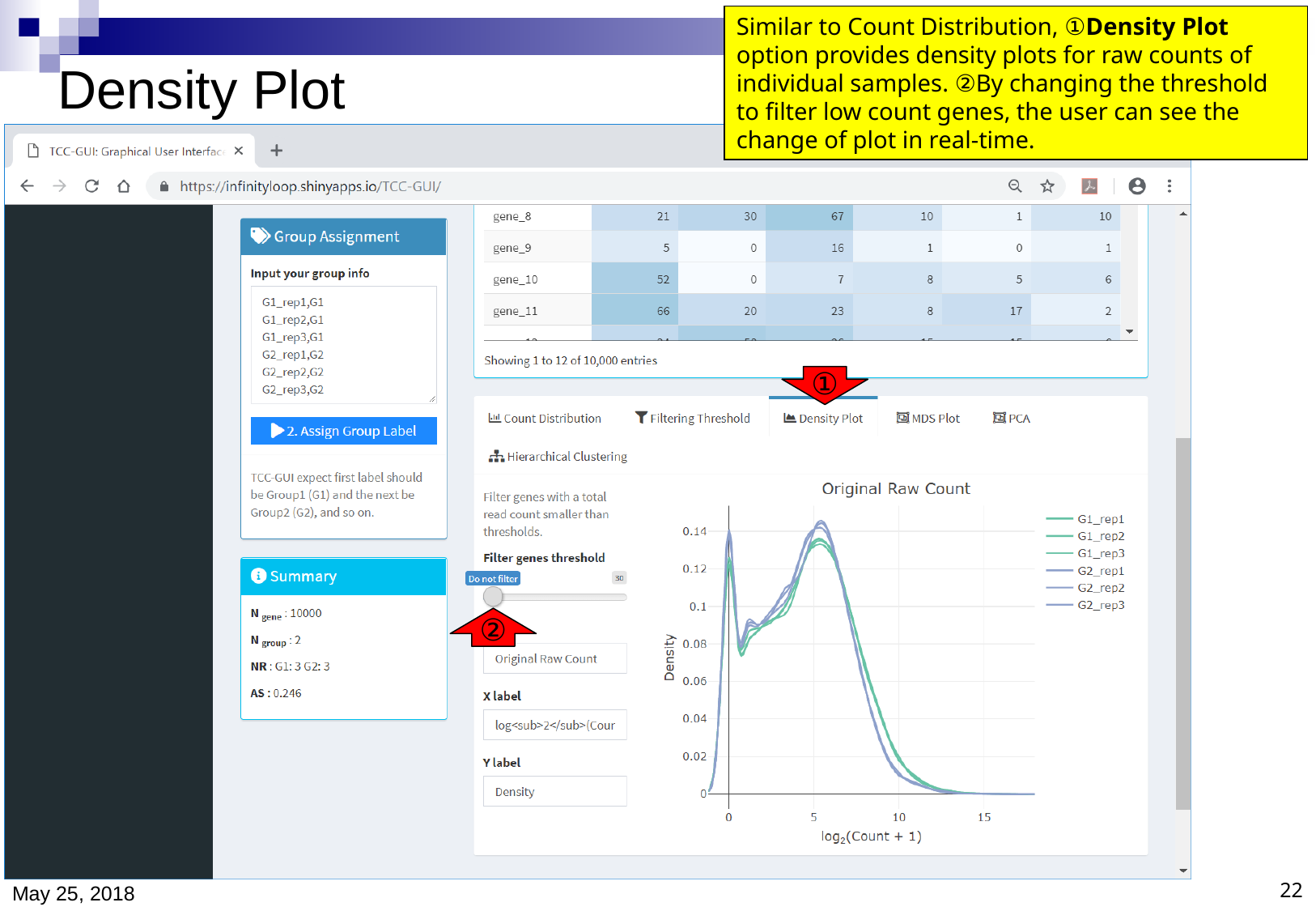

Similar to Count Distribution, ①Density Plot option provides density plots for raw counts of individual samples. ②By changing the threshold to filter low count genes, the user can see the change of plot in real-time.
# Density Plot
①
②
May 25, 2018
22

## Slide 23
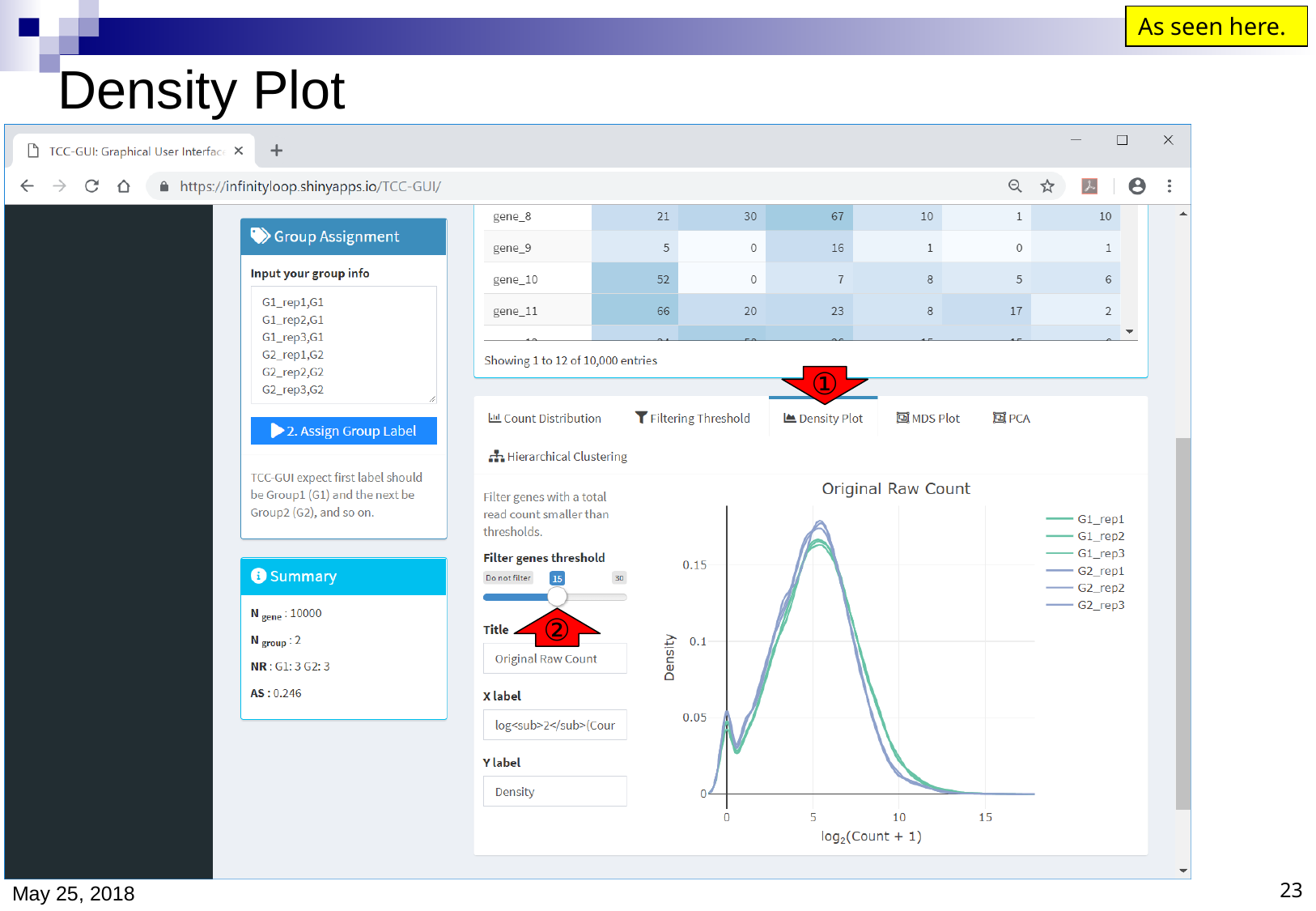

As seen here.
# Density Plot
①
②
May 25, 2018
23

## Slide 24
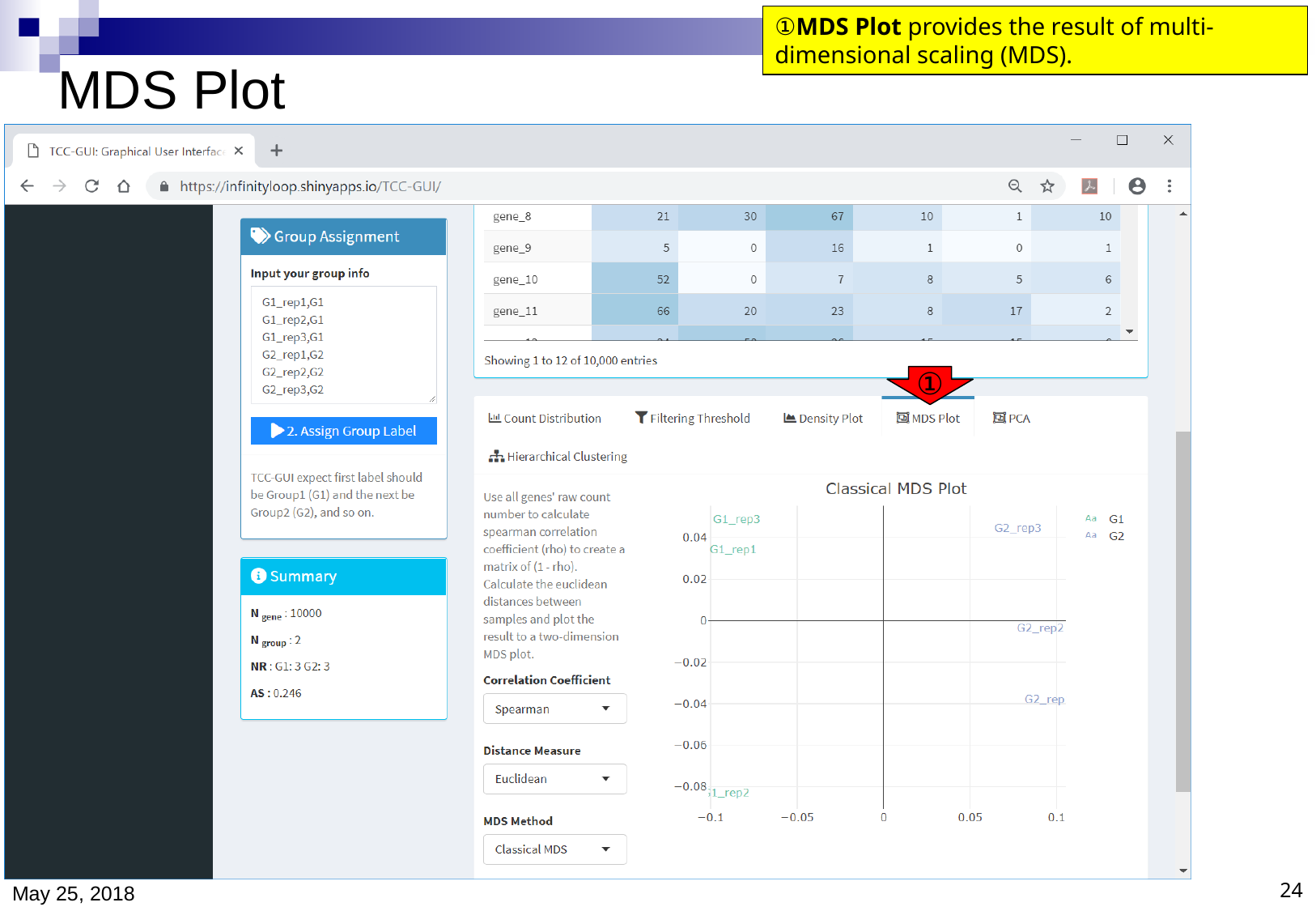

①MDS Plot provides the result of multi-dimensional scaling (MDS).
# MDS Plot
①
May 25, 2018
24

## Slide 25
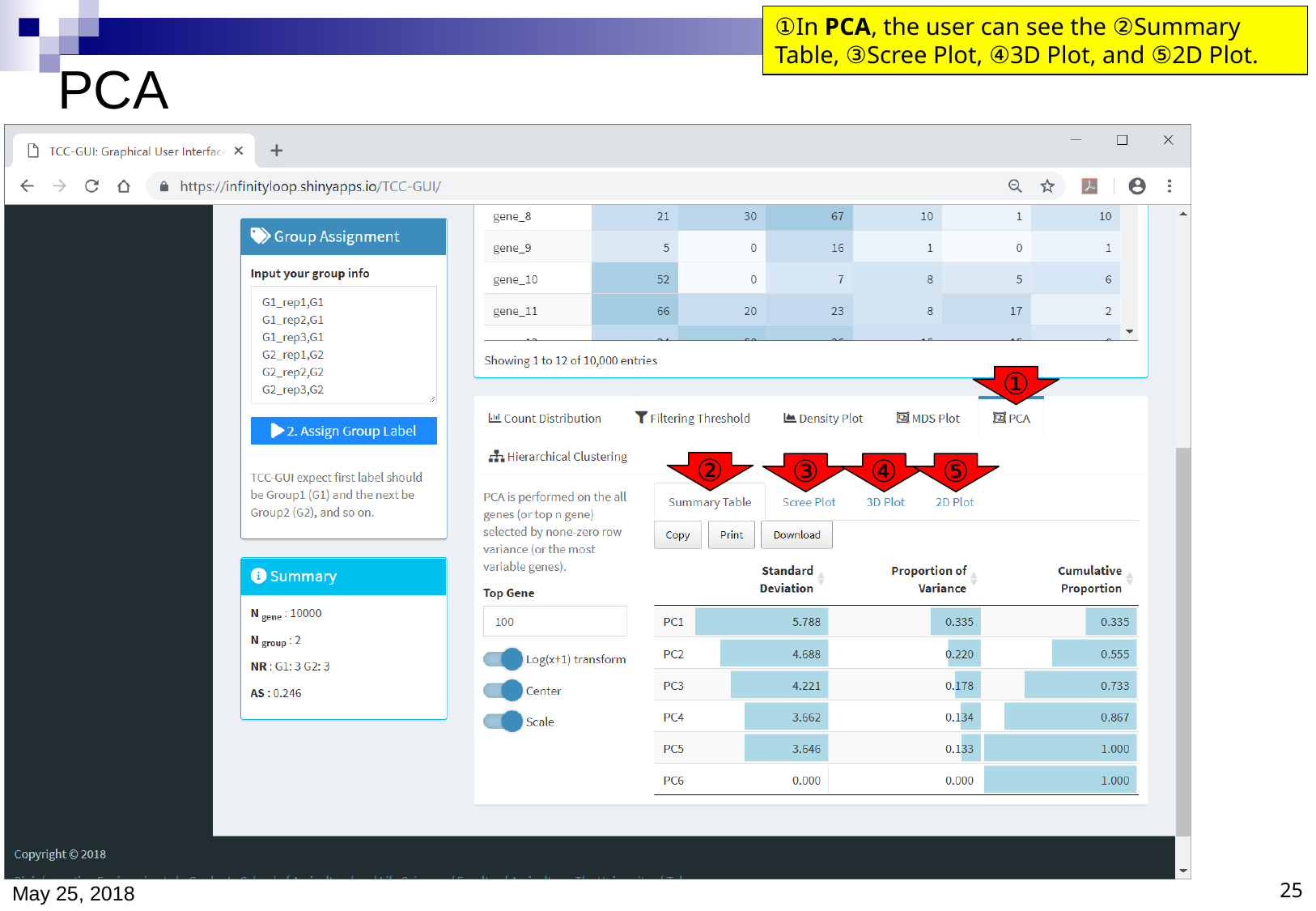

①In PCA, the user can see the ②Summary Table, ③Scree Plot, ④3D Plot, and ⑤2D Plot.
# PCA
①
②
③
④
⑤
May 25, 2018
25

## Slide 26
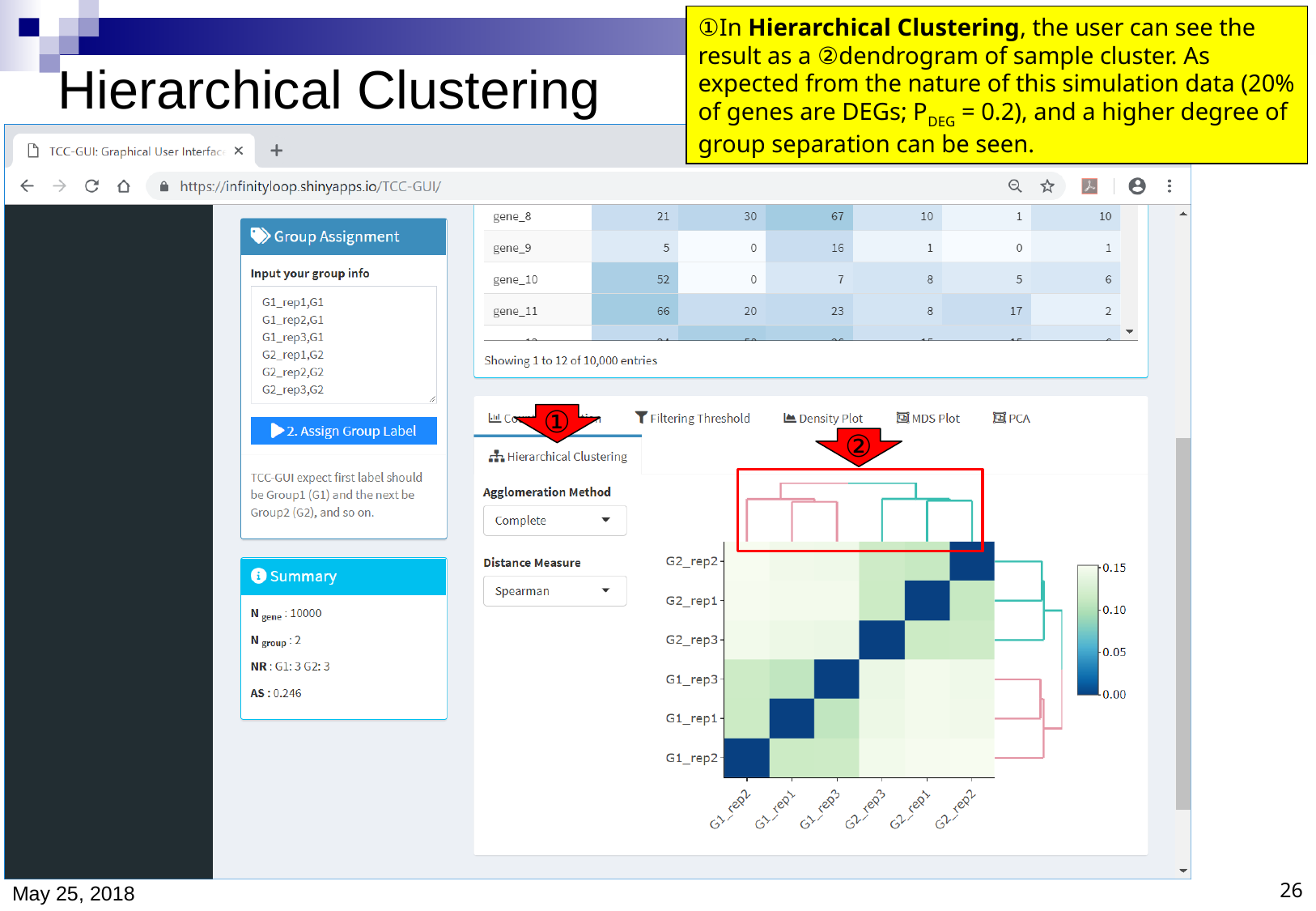

①In Hierarchical Clustering, the user can see the result as a ②dendrogram of sample cluster. As expected from the nature of this simulation data (20% of genes are DEGs; PDEG = 0.2), and a higher degree of group separation can be seen.
# Hierarchical Clustering
①
②
May 25, 2018
26

## Slide 27
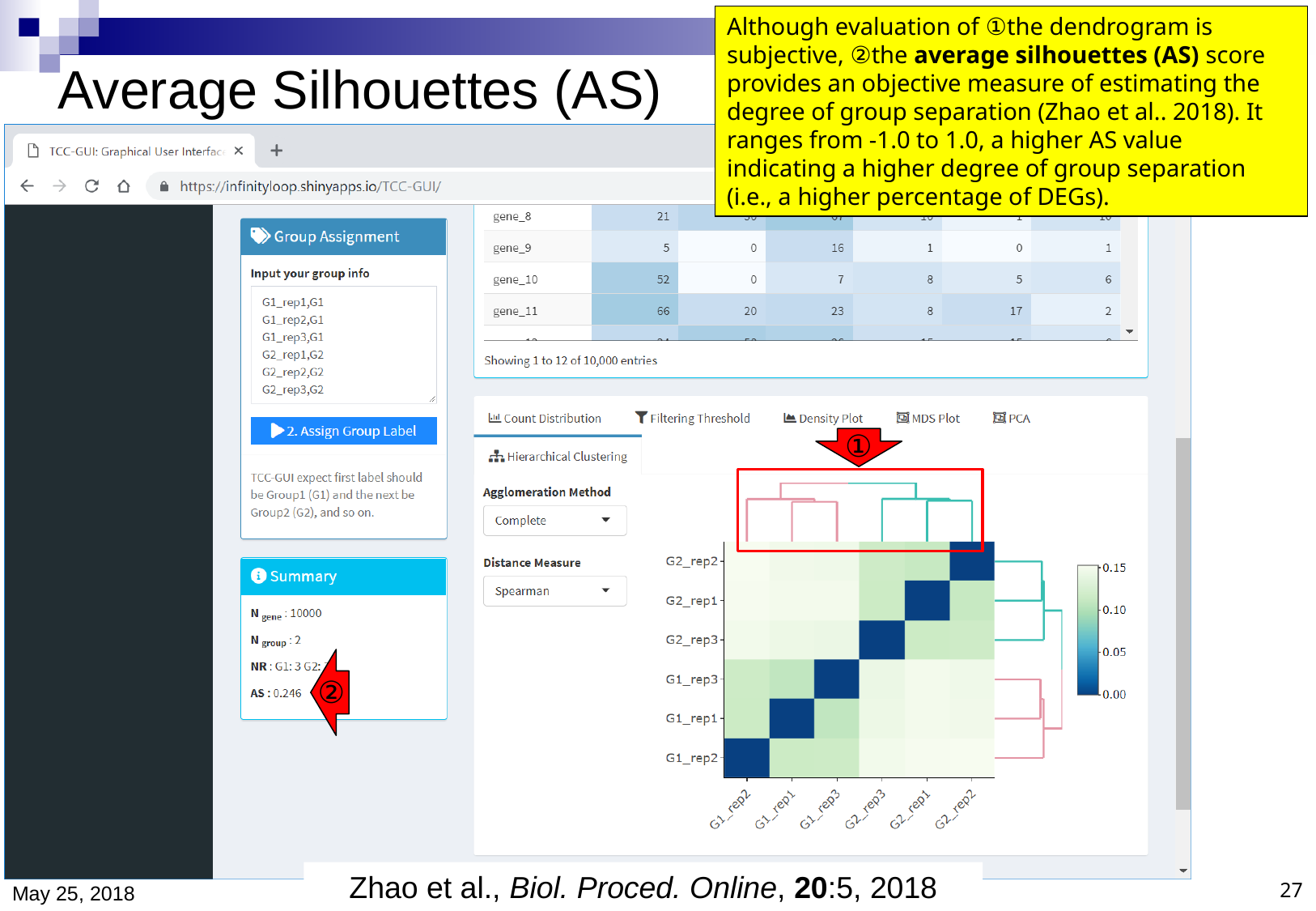

Although evaluation of ①the dendrogram is subjective, ②the average silhouettes (AS) score provides an objective measure of estimating the degree of group separation (Zhao et al.. 2018). It ranges from -1.0 to 1.0, a higher AS value indicating a higher degree of group separation (i.e., a higher percentage of DEGs).
# Average Silhouettes (AS)
①
②
May 25, 2018
27
Zhao et al., Biol. Proced. Online, 20:5, 2018

## Slide 28
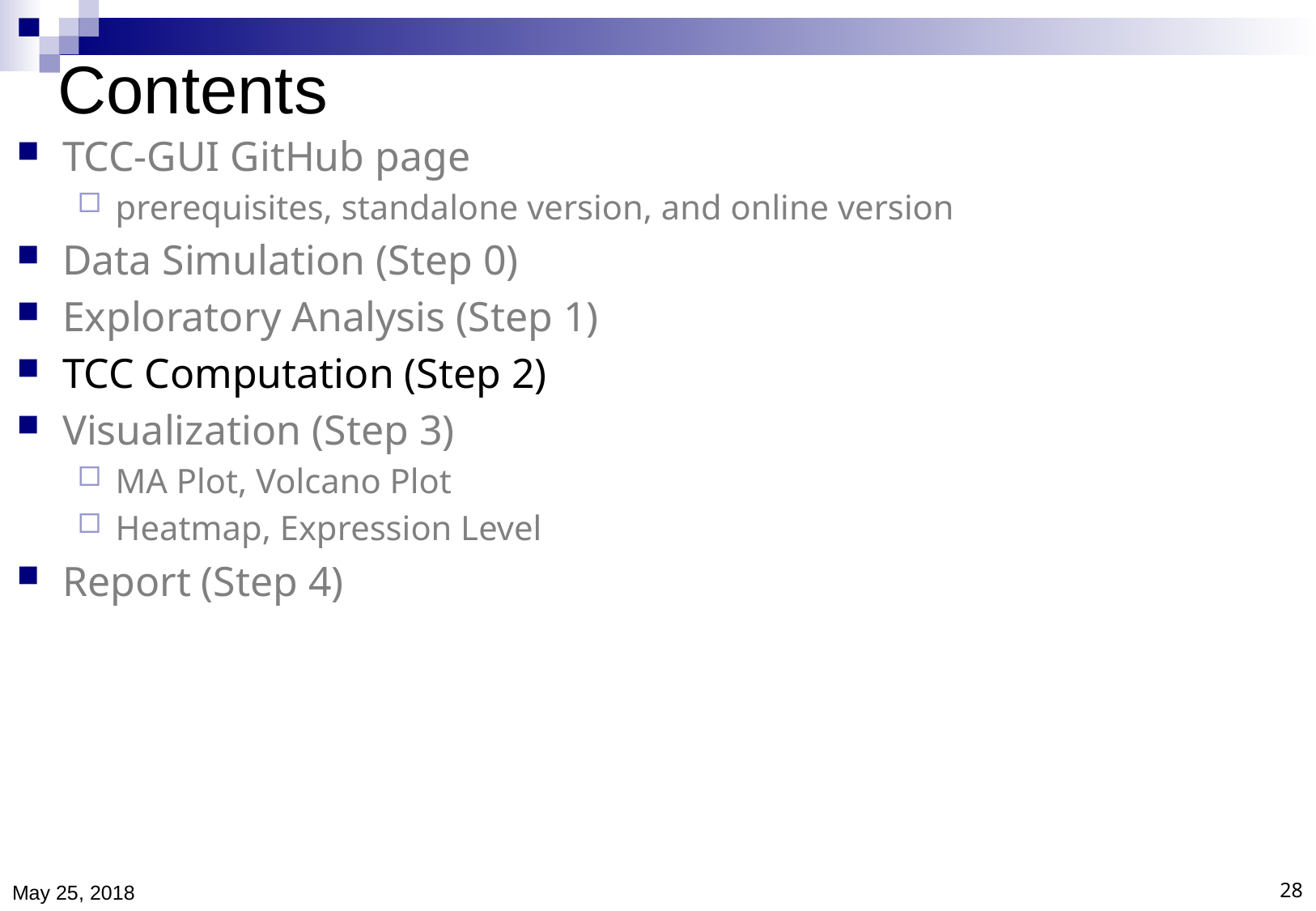

# Contents
TCC-GUI GitHub page
prerequisites, standalone version, and online version
Data Simulation (Step 0)
Exploratory Analysis (Step 1)
TCC Computation (Step 2)
Visualization (Step 3)
MA Plot, Volcano Plot
Heatmap, Expression Level
Report (Step 4)
May 25, 2018
28

## Slide 29
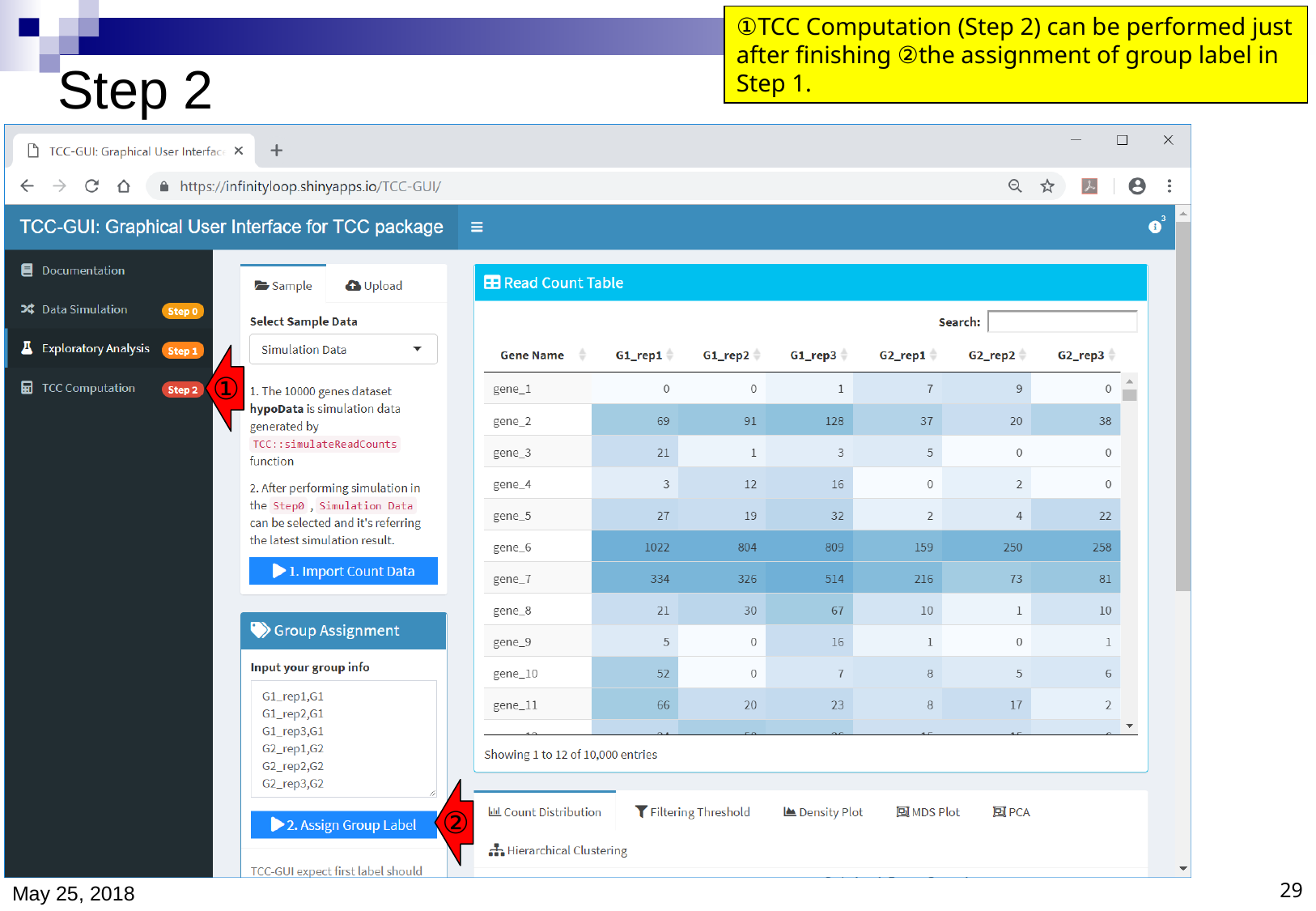

①TCC Computation (Step 2) can be performed just after finishing ②the assignment of group label in Step 1.
# Step 2
①
②
May 25, 2018
29

## Slide 30
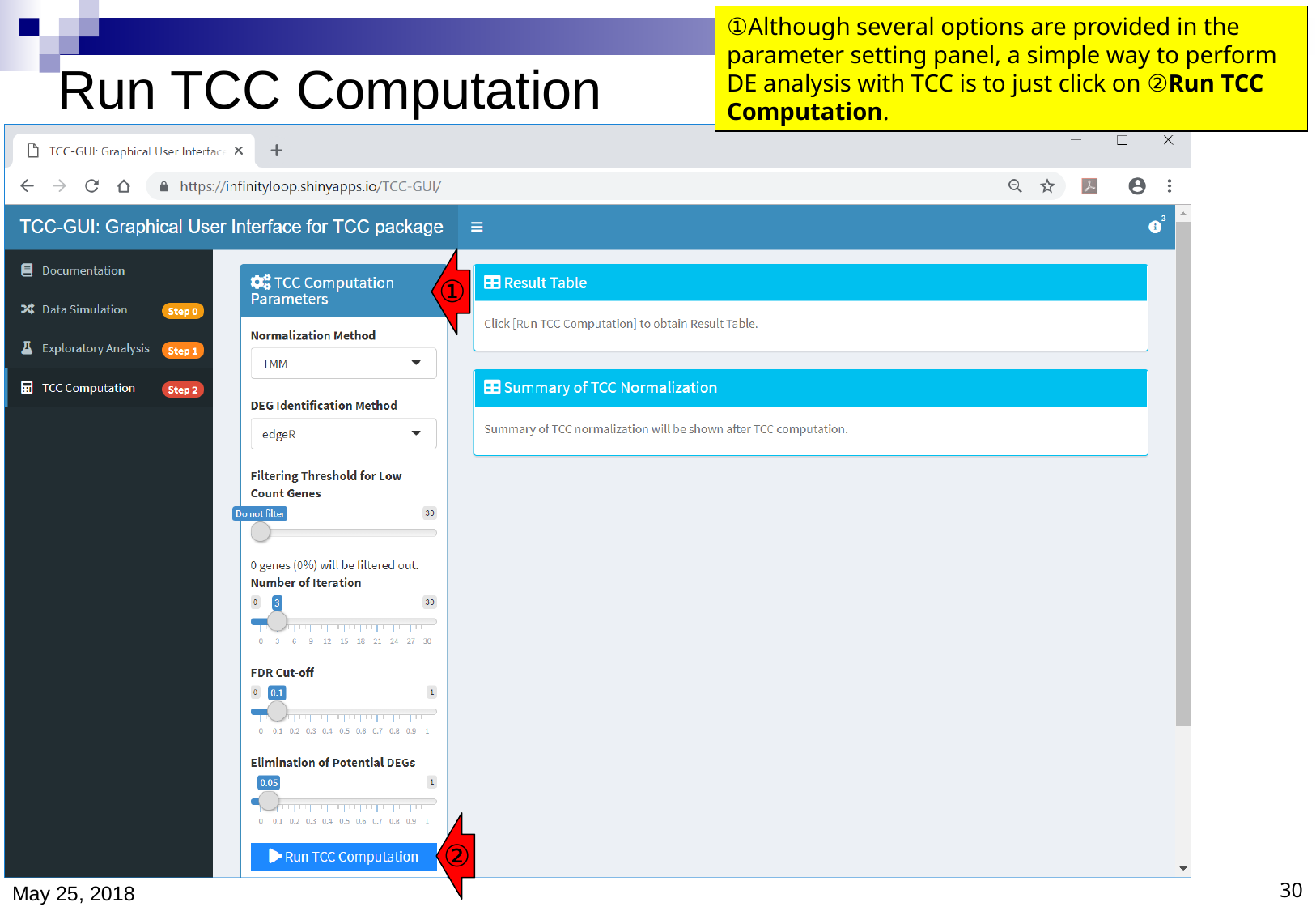

①Although several options are provided in the parameter setting panel, a simple way to perform DE analysis with TCC is to just click on ②Run TCC Computation.
# Run TCC Computation
①
②
May 25, 2018
30

## Slide 31
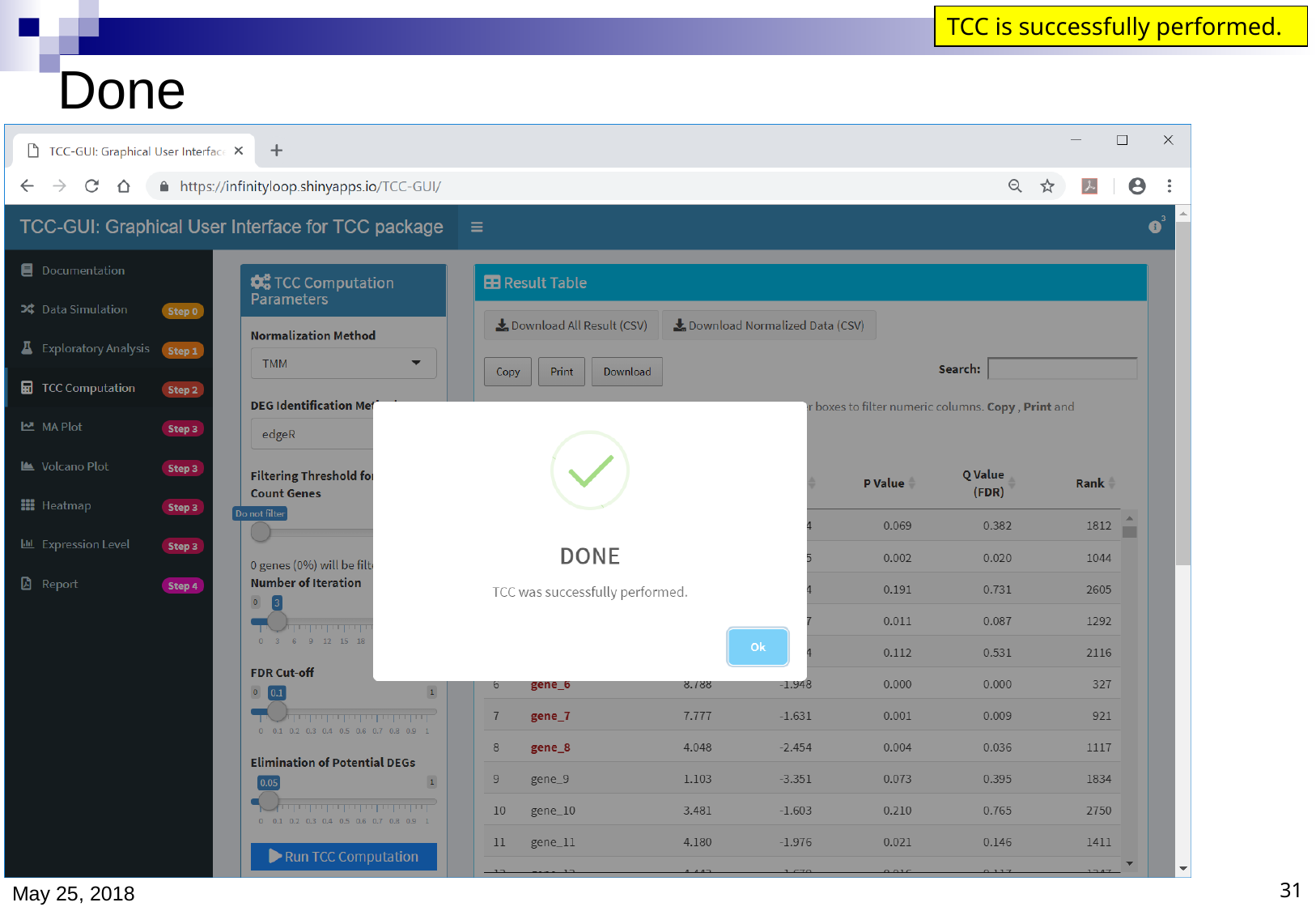

TCC is successfully performed.
# Done
May 25, 2018
31

## Slide 32
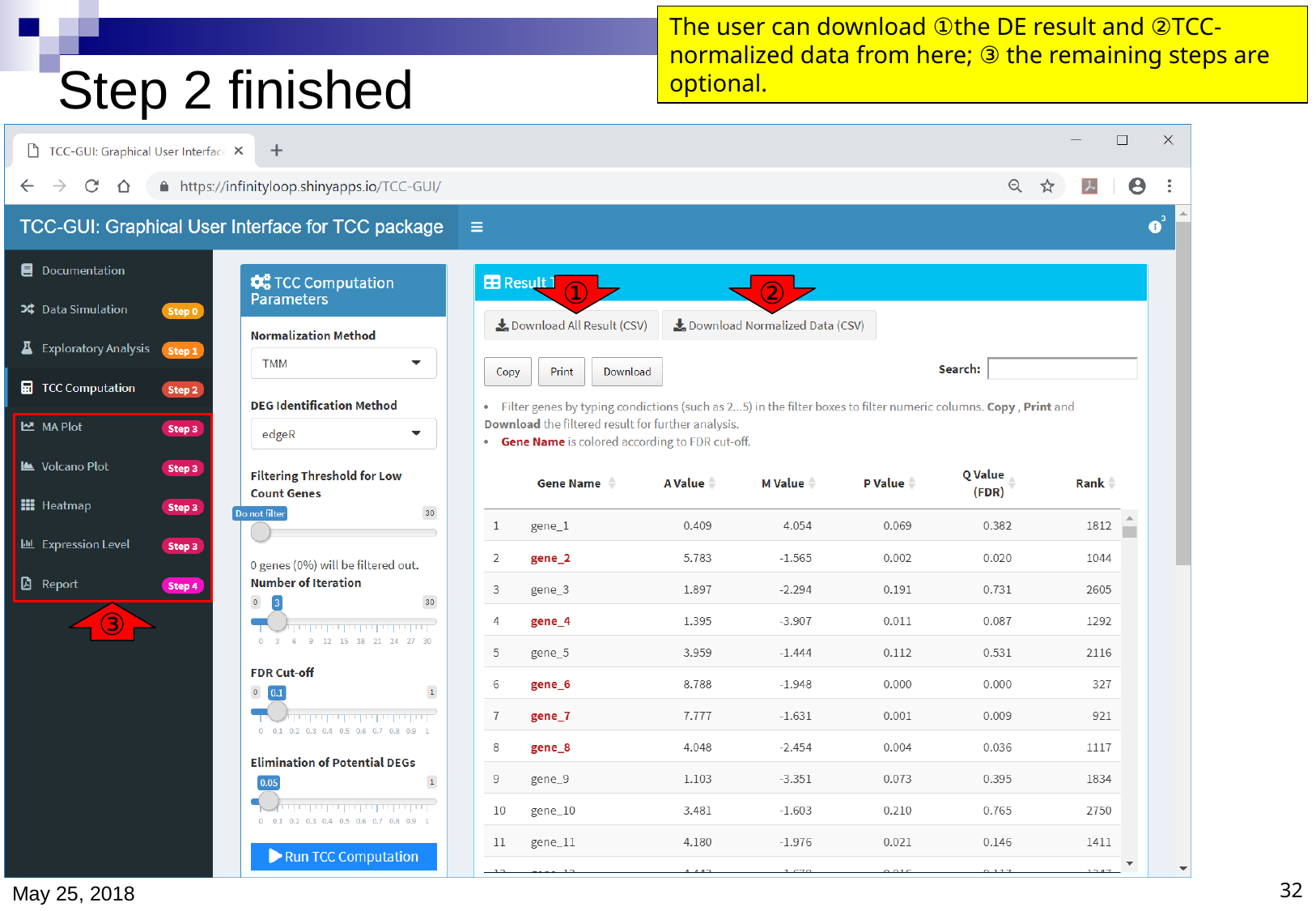

The user can download ①the DE result and ②TCC-normalized data from here; ③ the remaining steps are optional.
# Step 2 finished
①
②
③
May 25, 2018
32

## Slide 33
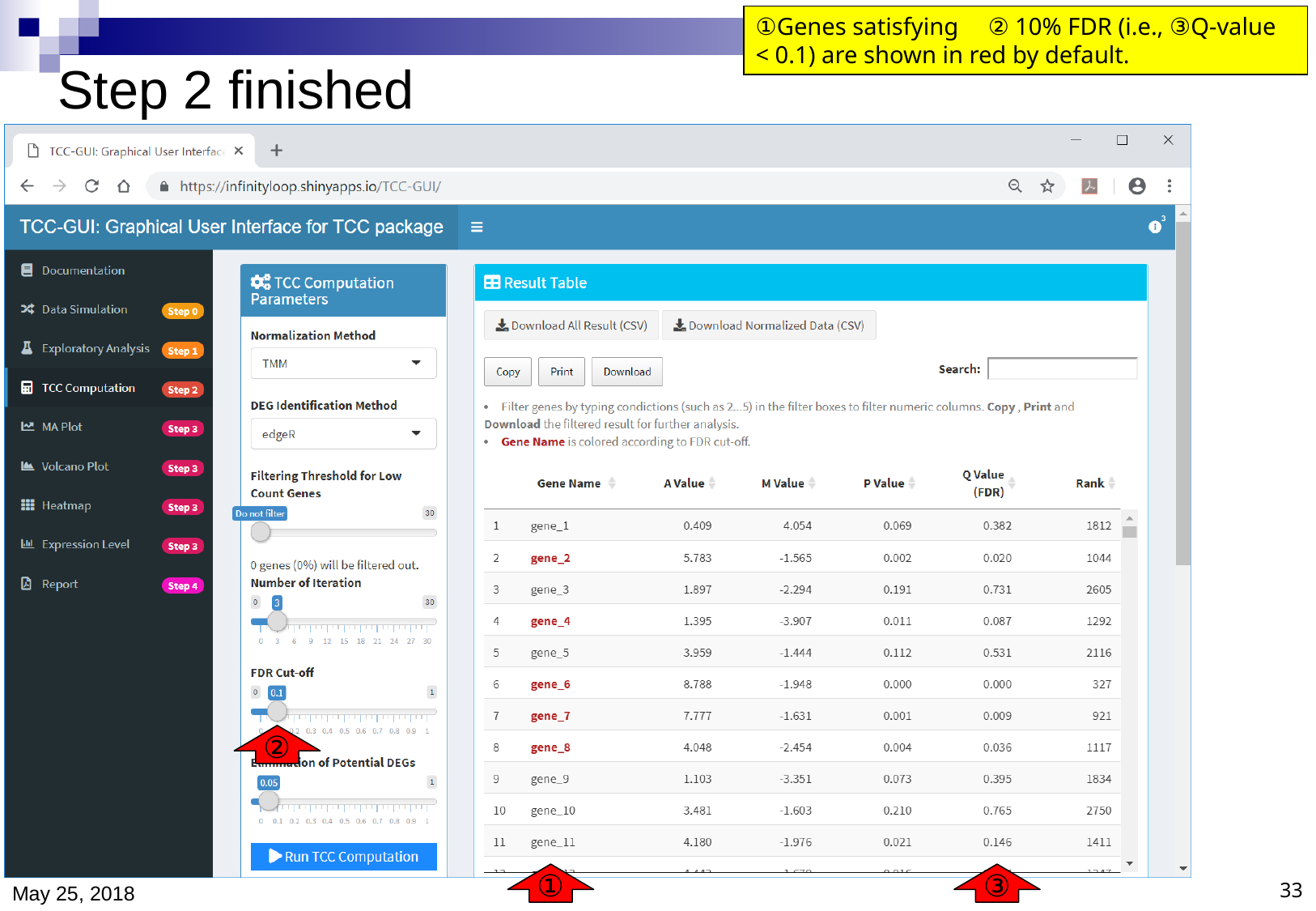

①Genes satisfying　②10% FDR (i.e., ③Q-value < 0.1) are shown in red by default.
# Step 2 finished
②
May 25, 2018
33
①
③

## Slide 34
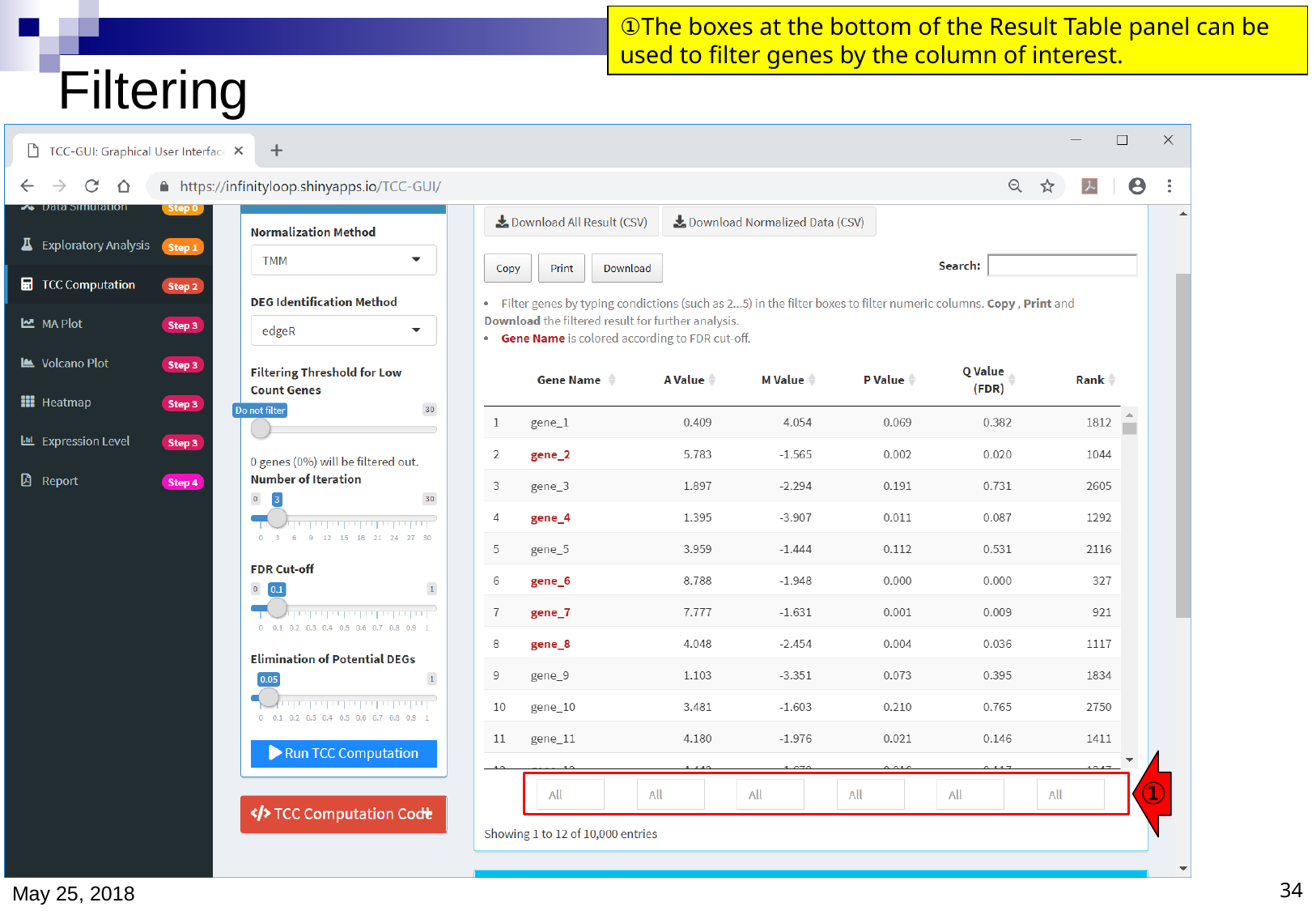

①The boxes at the bottom of the Result Table panel can be used to filter genes by the column of interest.
# Filtering
①
May 25, 2018
34

## Slide 35
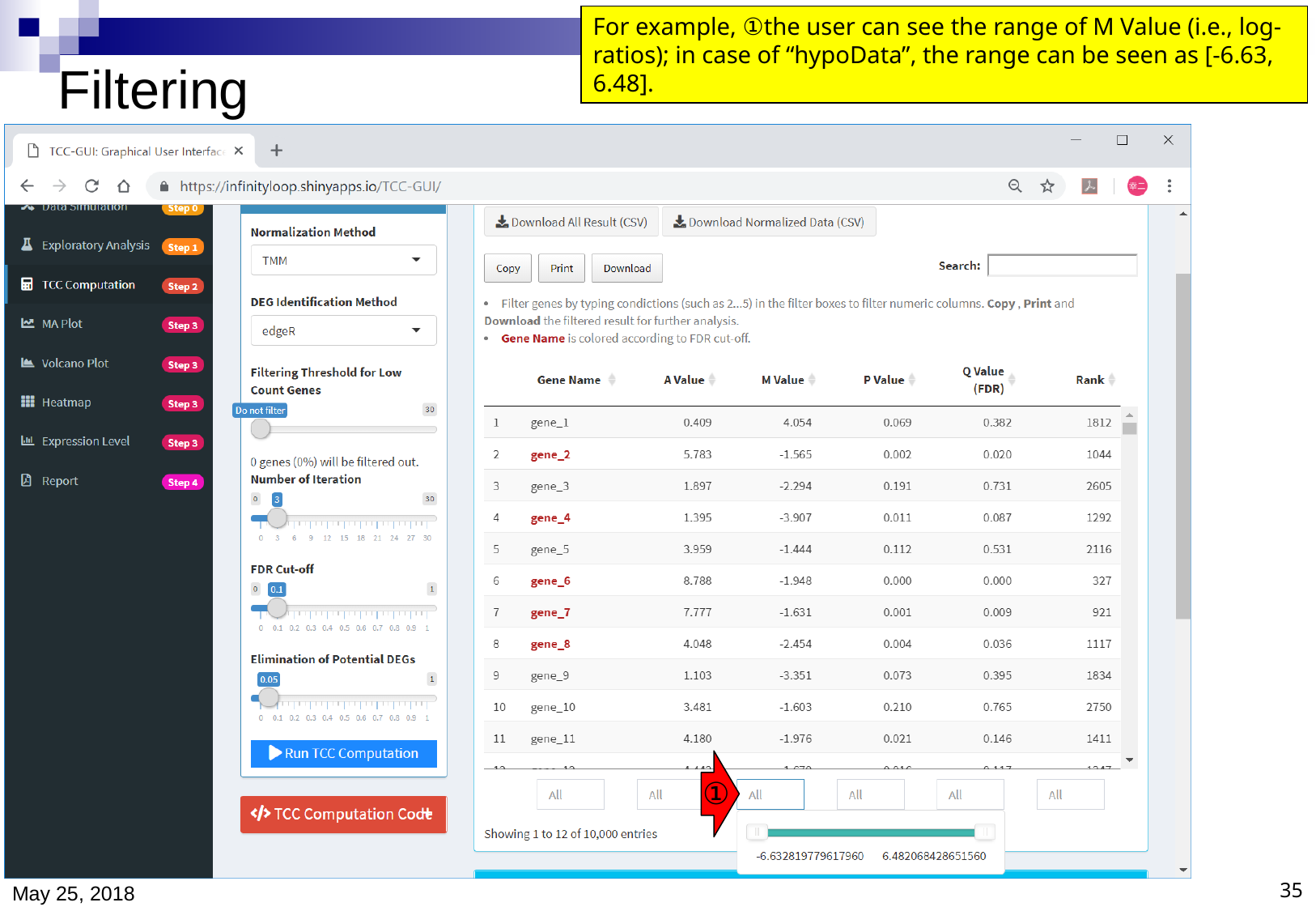

For example, ①the user can see the range of M Value (i.e., log-ratios); in case of “hypoData”, the range can be seen as [-6.63, 6.48].
# Filtering
①
May 25, 2018
35

## Slide 36
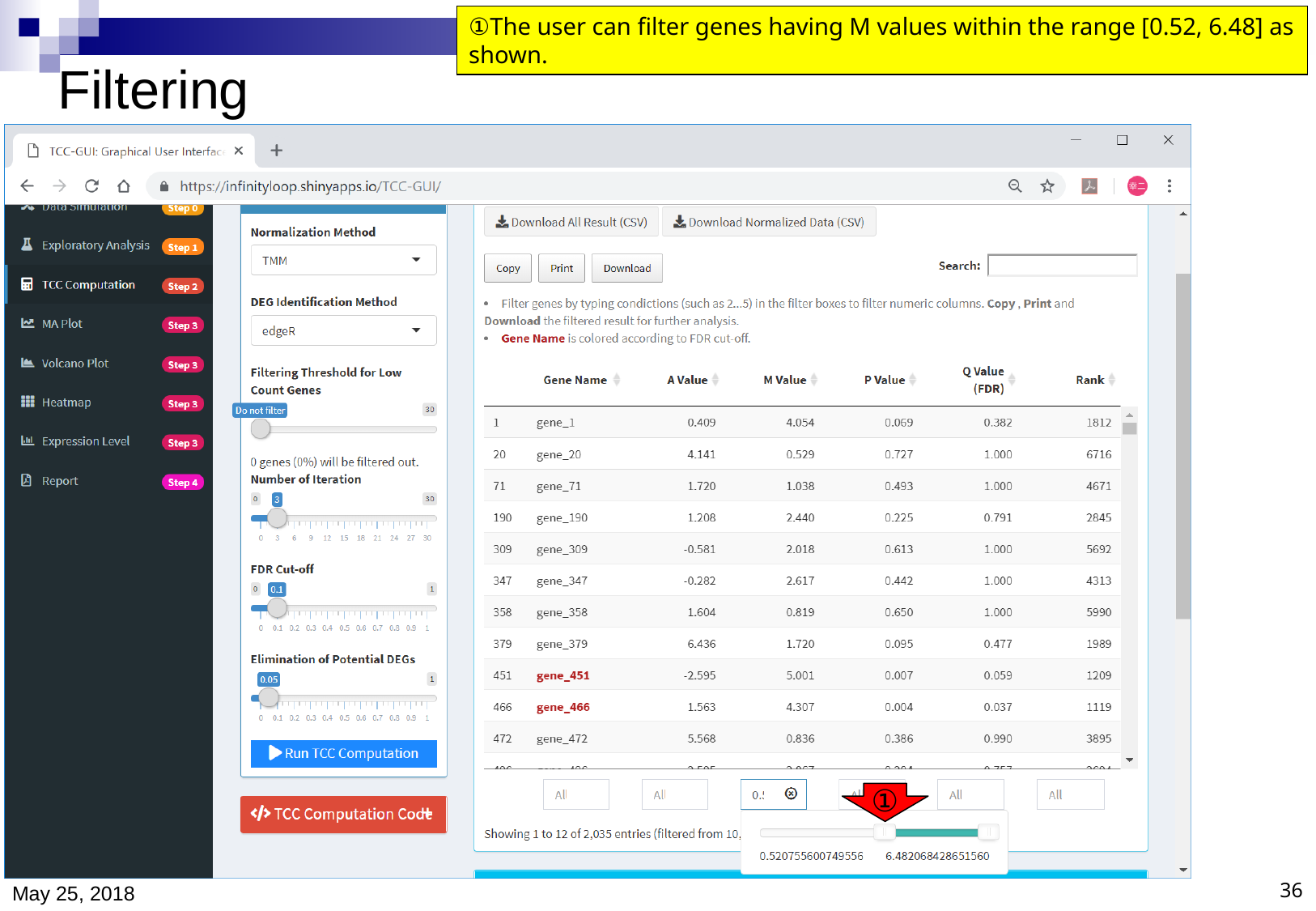

①The user can filter genes having M values within the range [0.52, 6.48] as shown.
# Filtering
①
May 25, 2018
36

## Slide 37
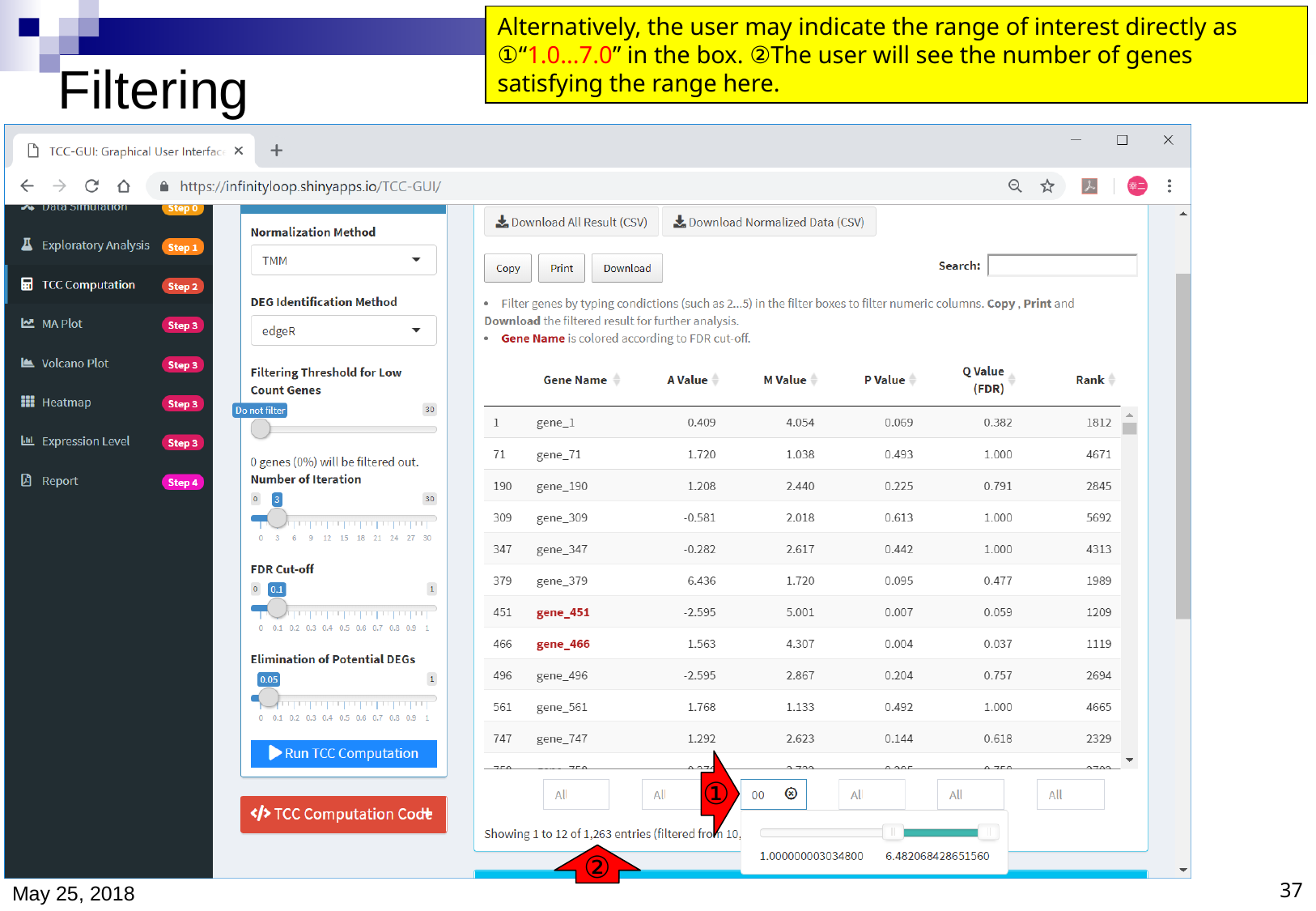

Alternatively, the user may indicate the range of interest directly as ①“1.0...7.0” in the box. ②The user will see the number of genes satisfying the range here.
# Filtering
①
②
May 25, 2018
37

## Slide 38
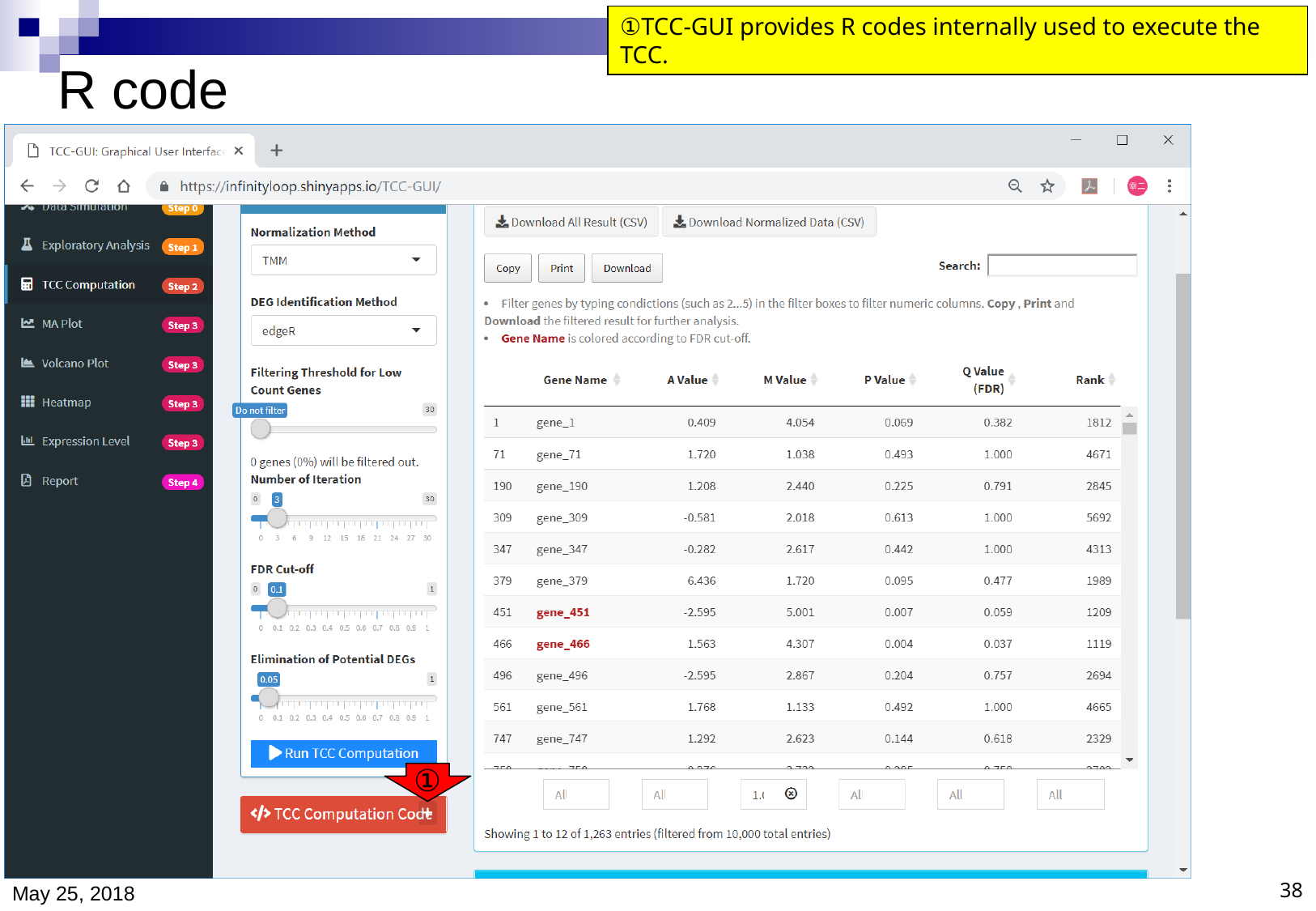

①TCC-GUI provides R codes internally used to execute the TCC.
# R code
①
May 25, 2018
38

## Slide 39
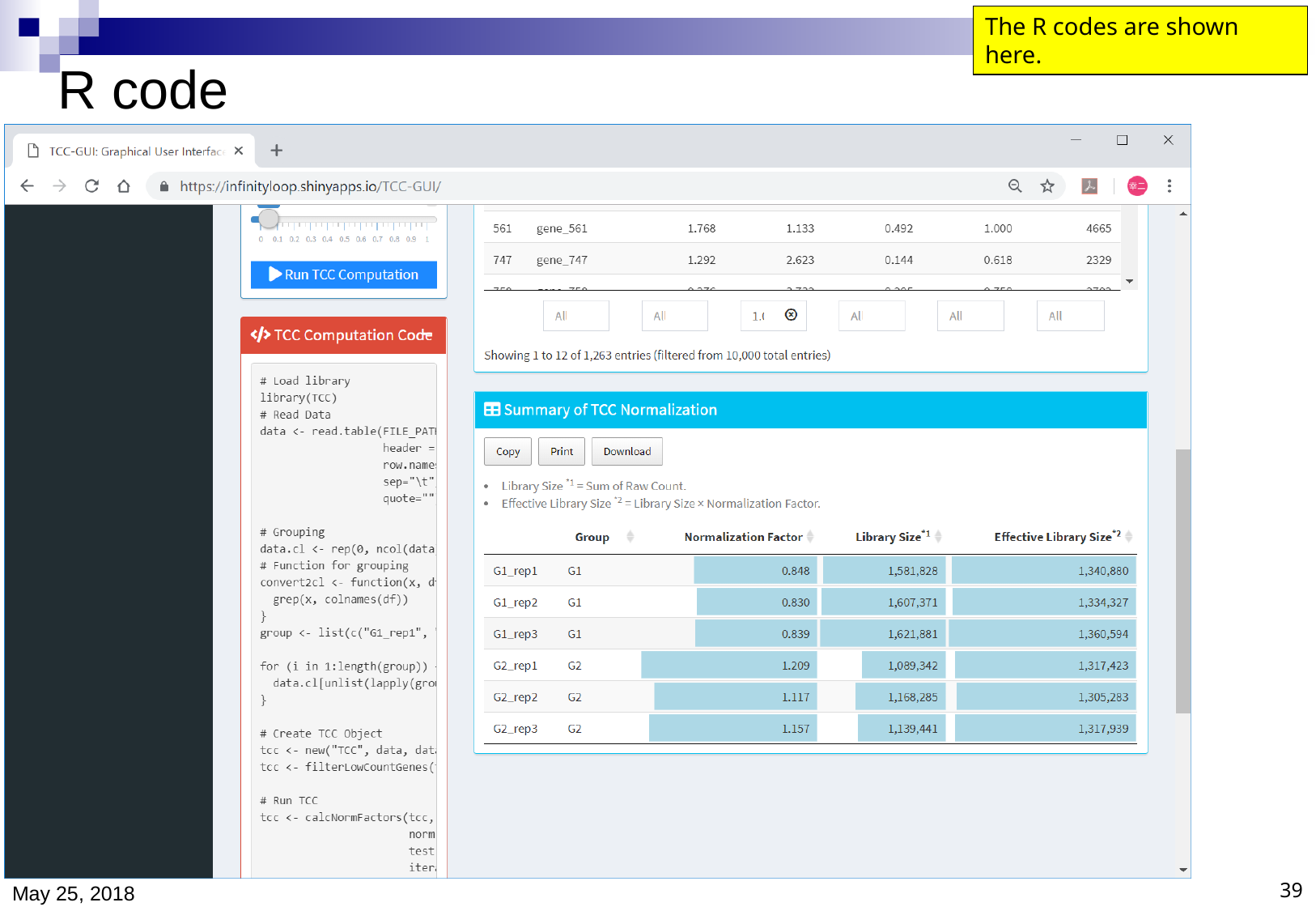

The R codes are shown here.
# R code
May 25, 2018
39

## Slide 40
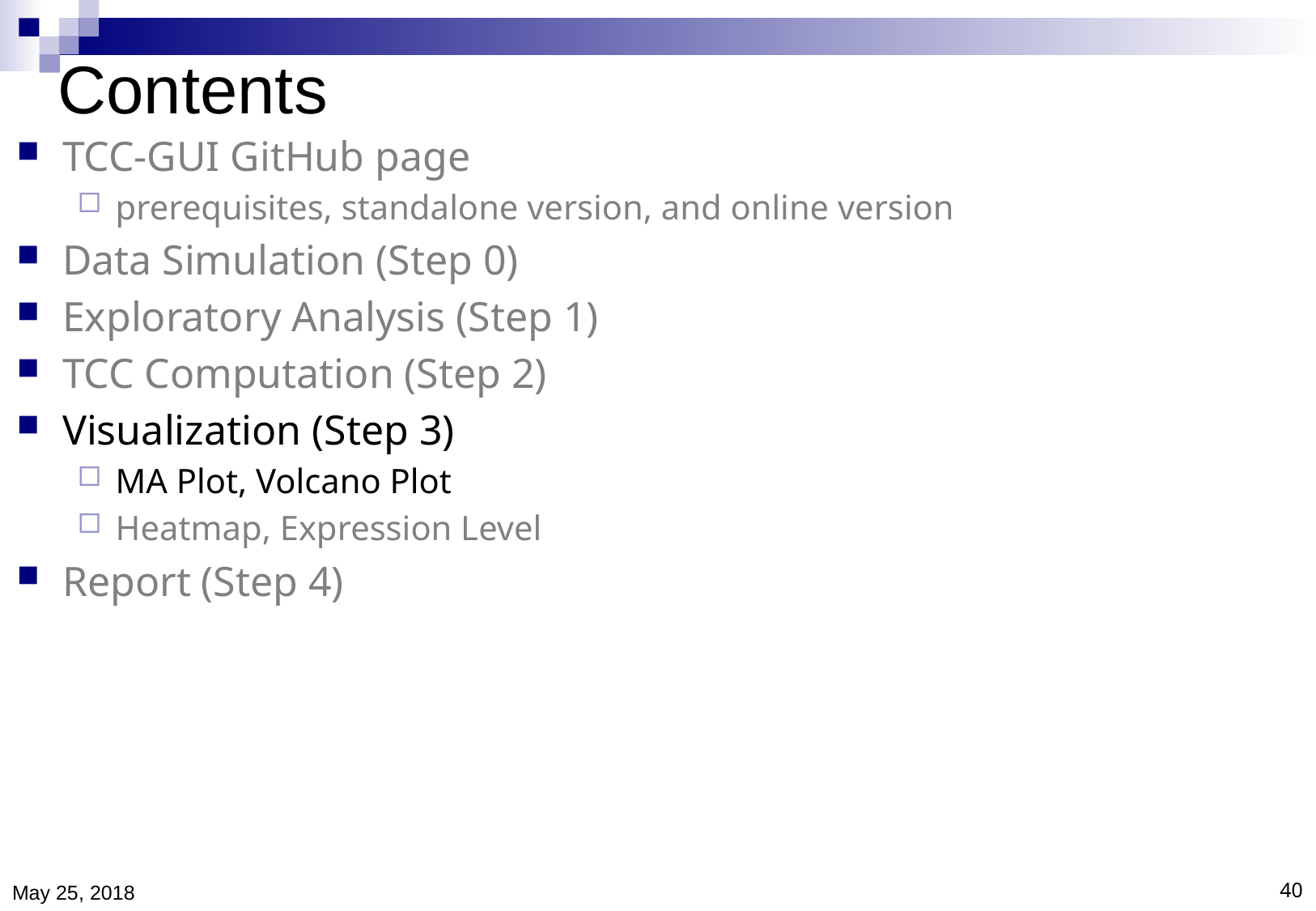

# Contents
TCC-GUI GitHub page
prerequisites, standalone version, and online version
Data Simulation (Step 0)
Exploratory Analysis (Step 1)
TCC Computation (Step 2)
Visualization (Step 3)
MA Plot, Volcano Plot
Heatmap, Expression Level
Report (Step 4)
May 25, 2018
40

## Slide 41
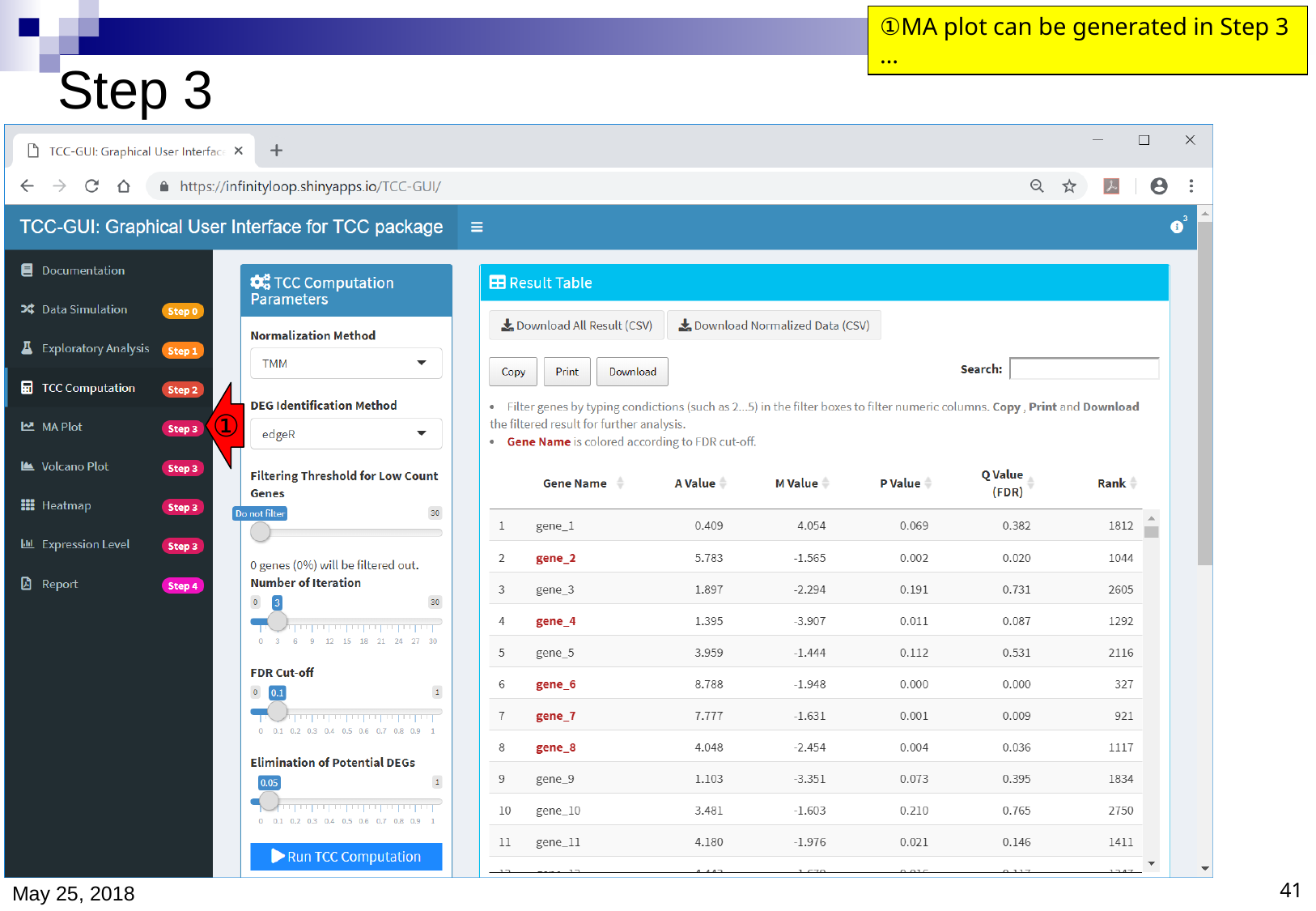

①MA plot can be generated in Step 3 …
# Step 3
①
May 25, 2018
41

## Slide 42
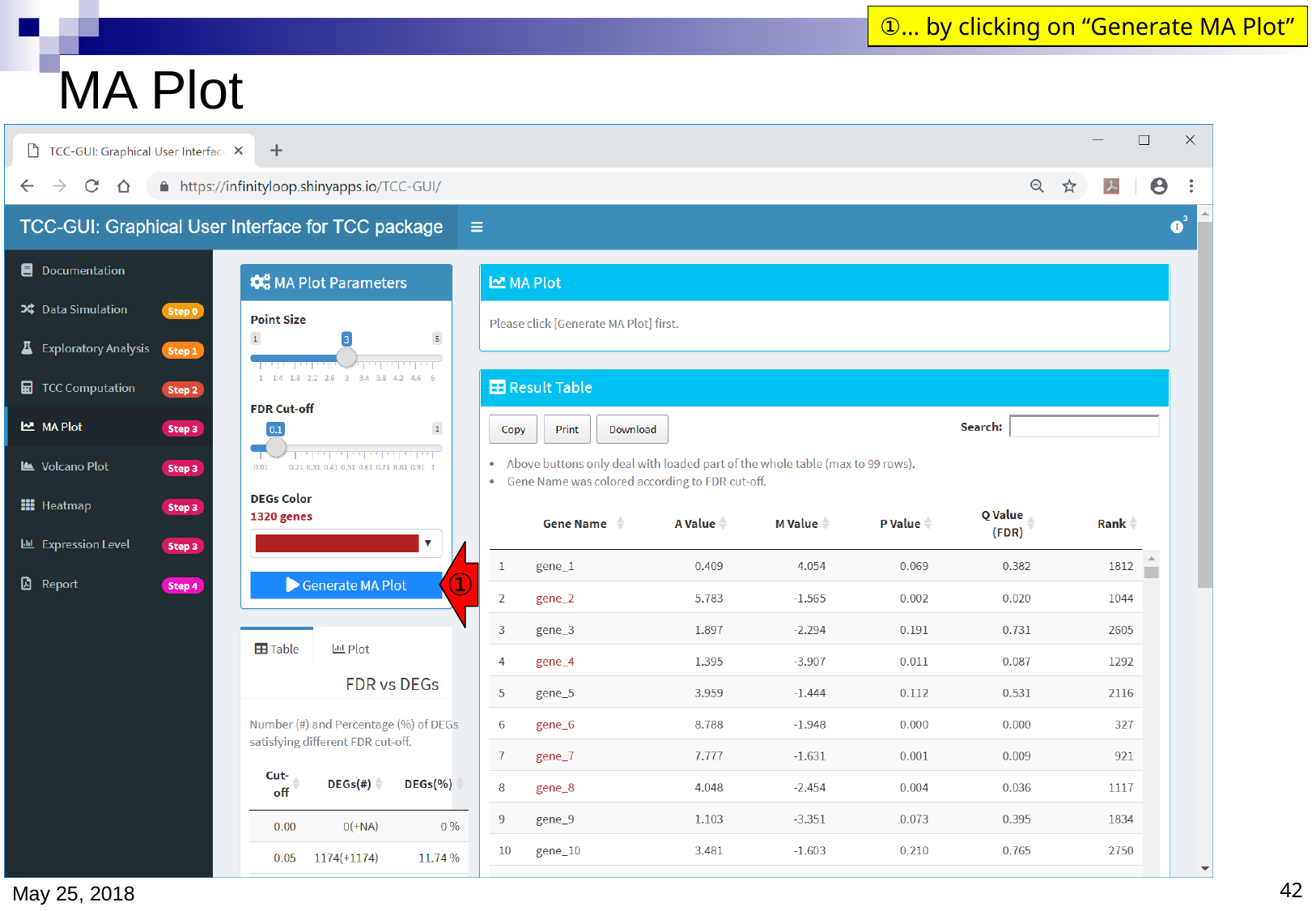

①… by clicking on “Generate MA Plot”
# MA Plot
①
May 25, 2018
42

## Slide 43
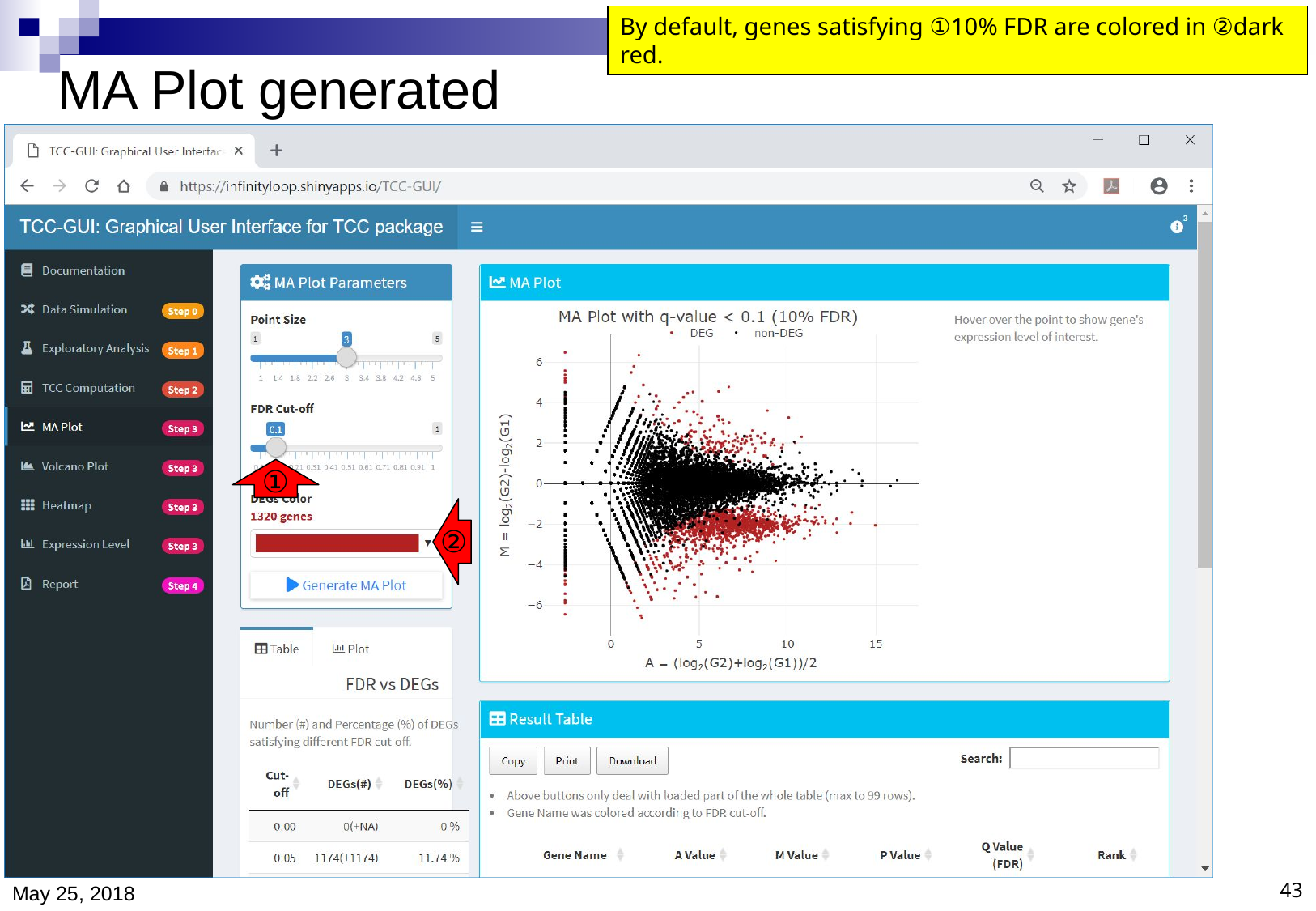

By default, genes satisfying ①10% FDR are colored in ②dark red.
# MA Plot generated
①
②
May 25, 2018
43

## Slide 44
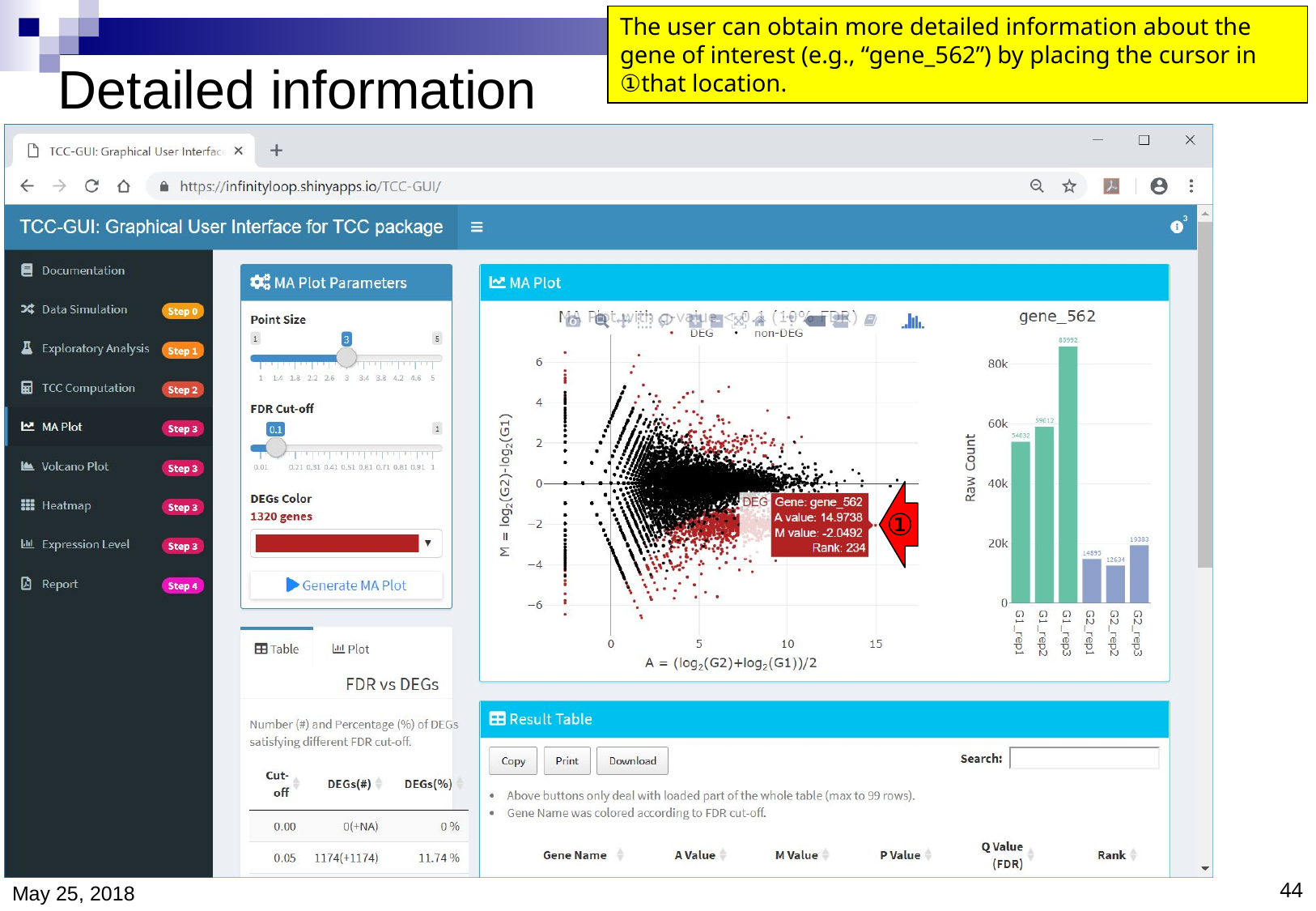

The user can obtain more detailed information about the gene of interest (e.g., “gene_562”) by placing the cursor in ①that location.
# Detailed information
①
May 25, 2018
44

## Slide 45
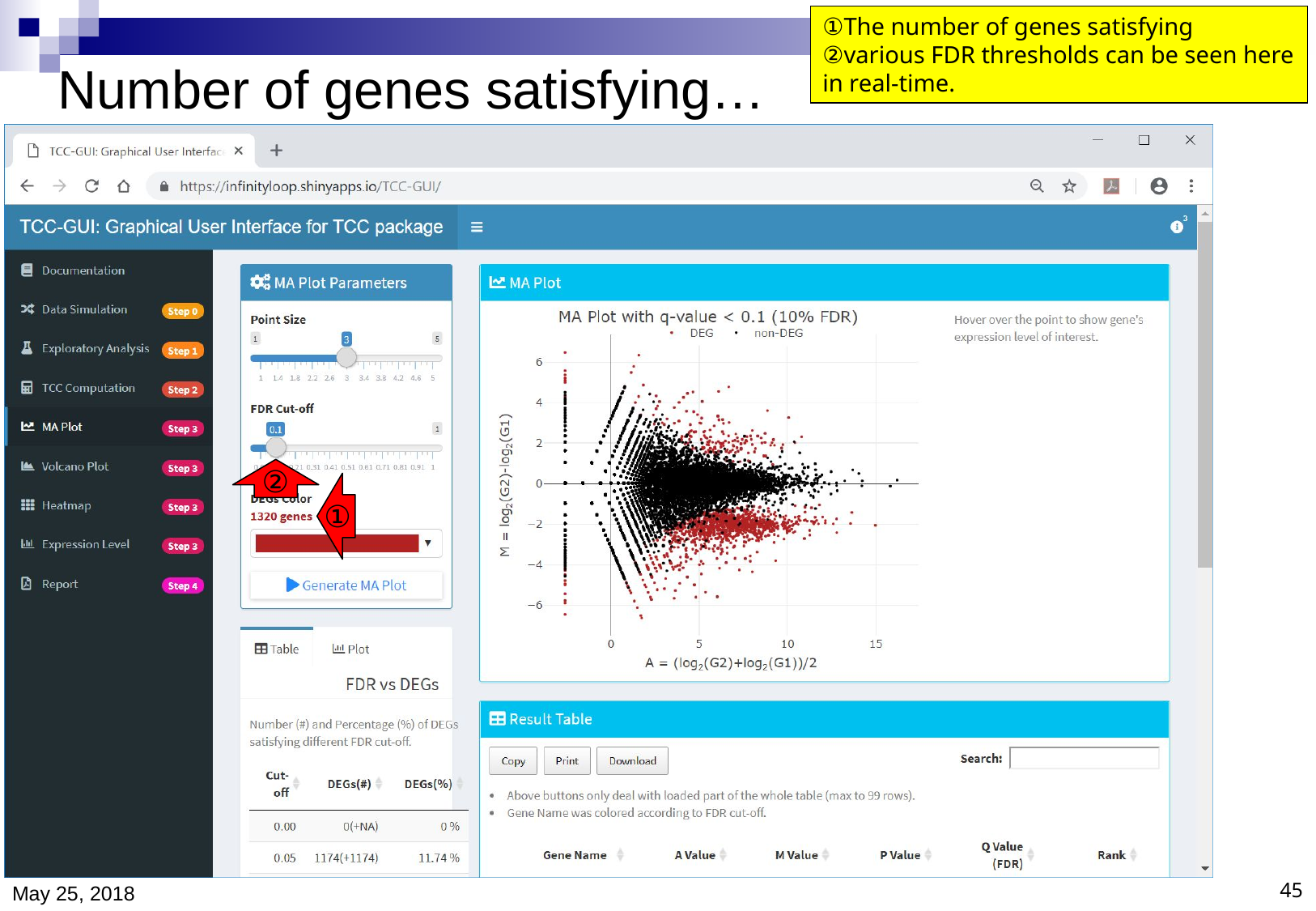

①The number of genes satisfying ②various FDR thresholds can be seen here in real-time.
# Number of genes satisfying…
②
①
May 25, 2018
45

## Slide 46
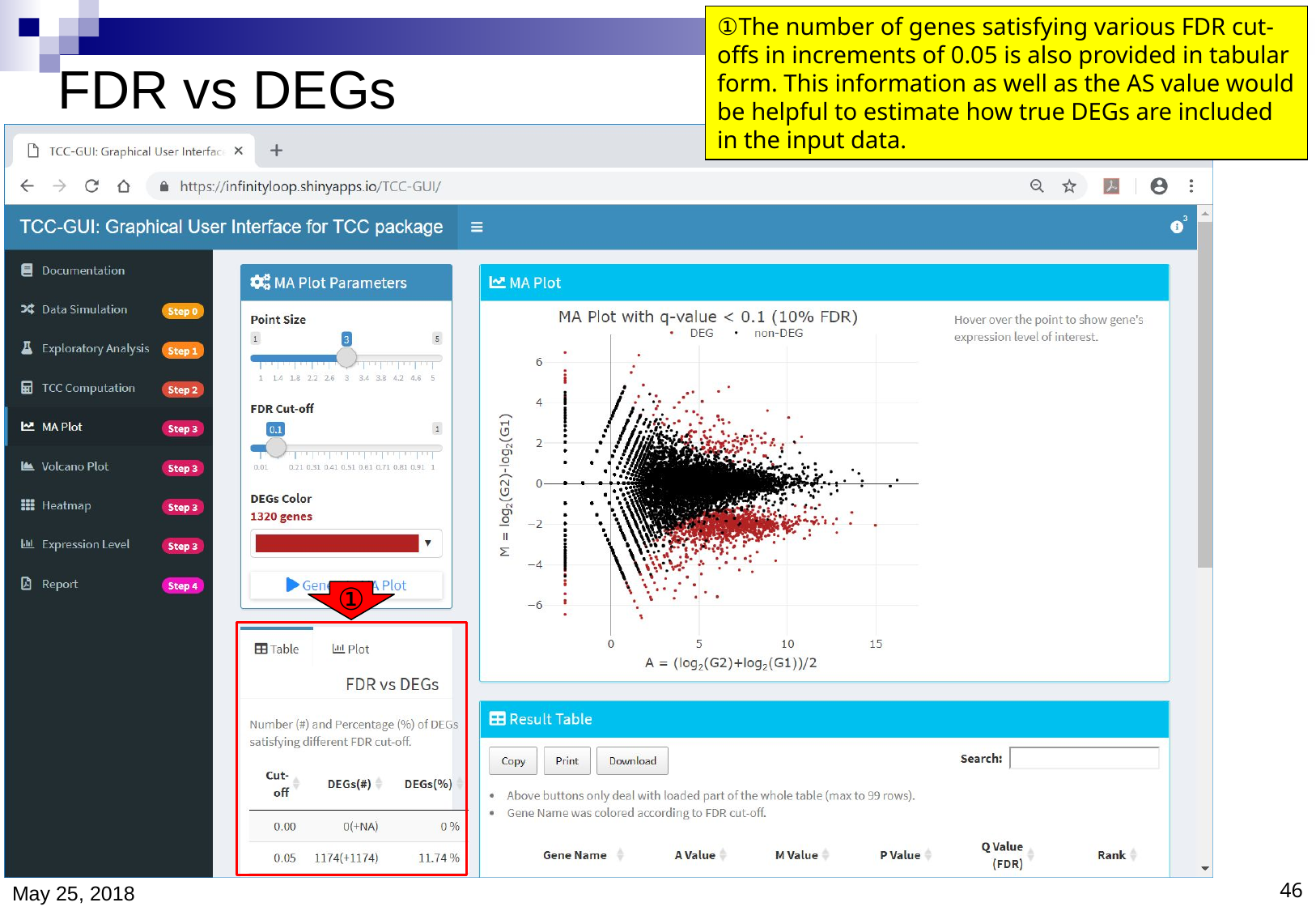

①The number of genes satisfying various FDR cut-offs in increments of 0.05 is also provided in tabular form. This information as well as the AS value would be helpful to estimate how true DEGs are included in the input data.
# FDR vs DEGs
①
May 25, 2018
46

## Slide 47
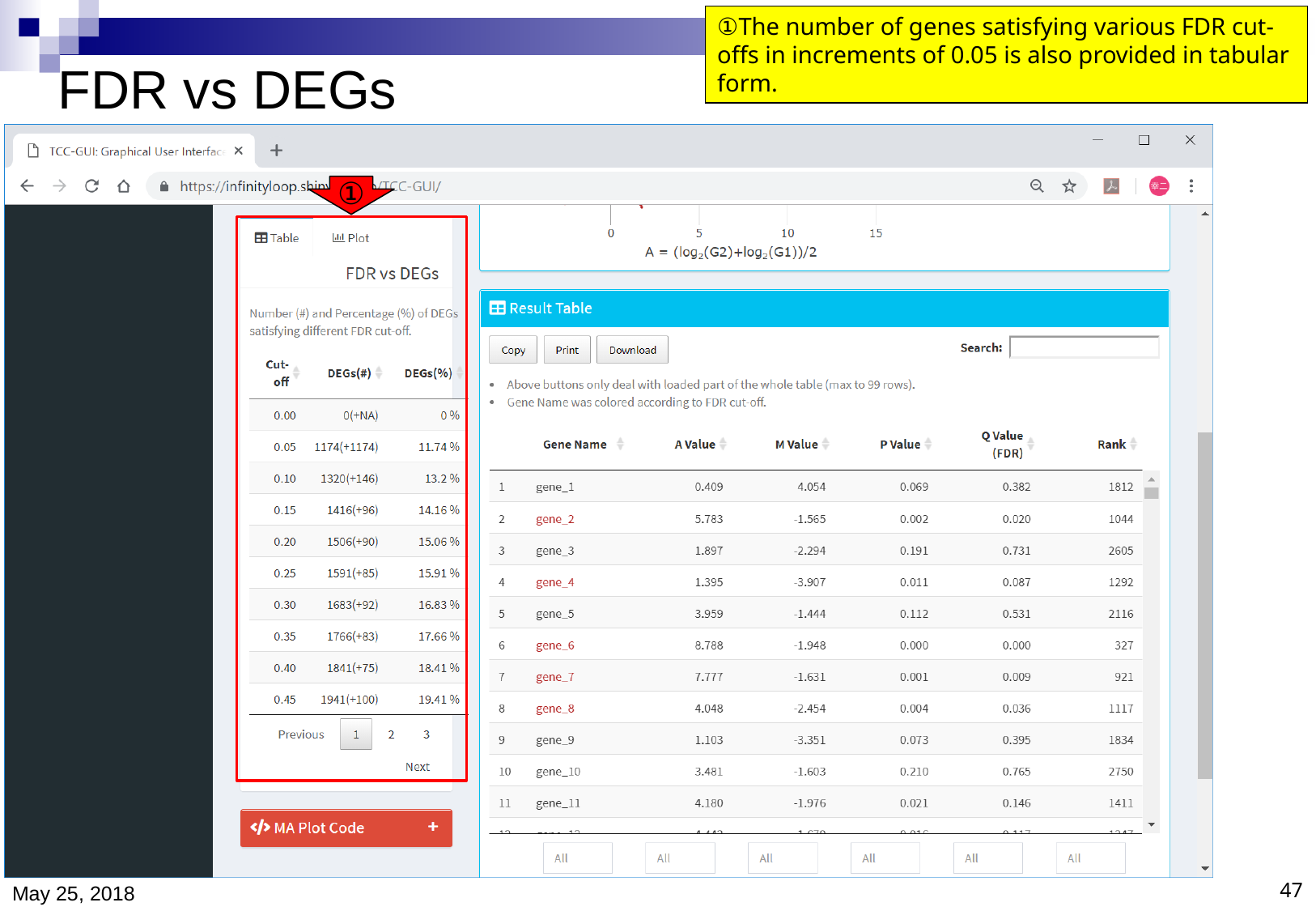

①The number of genes satisfying various FDR cut-offs in increments of 0.05 is also provided in tabular form.
# FDR vs DEGs
①
May 25, 2018
47

## Slide 48
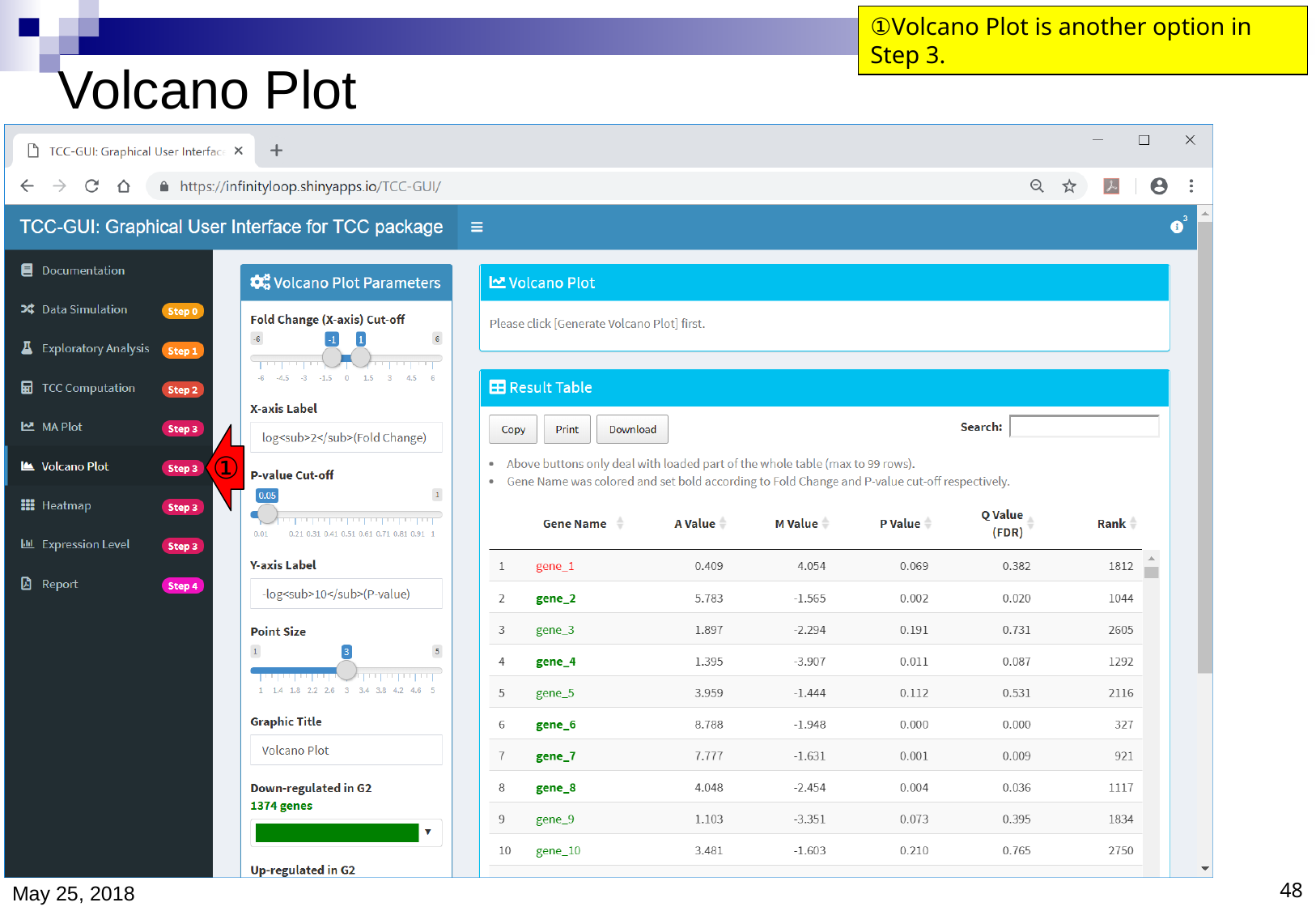

①Volcano Plot is another option in Step 3.
# Volcano Plot
①
May 25, 2018
48

## Slide 49
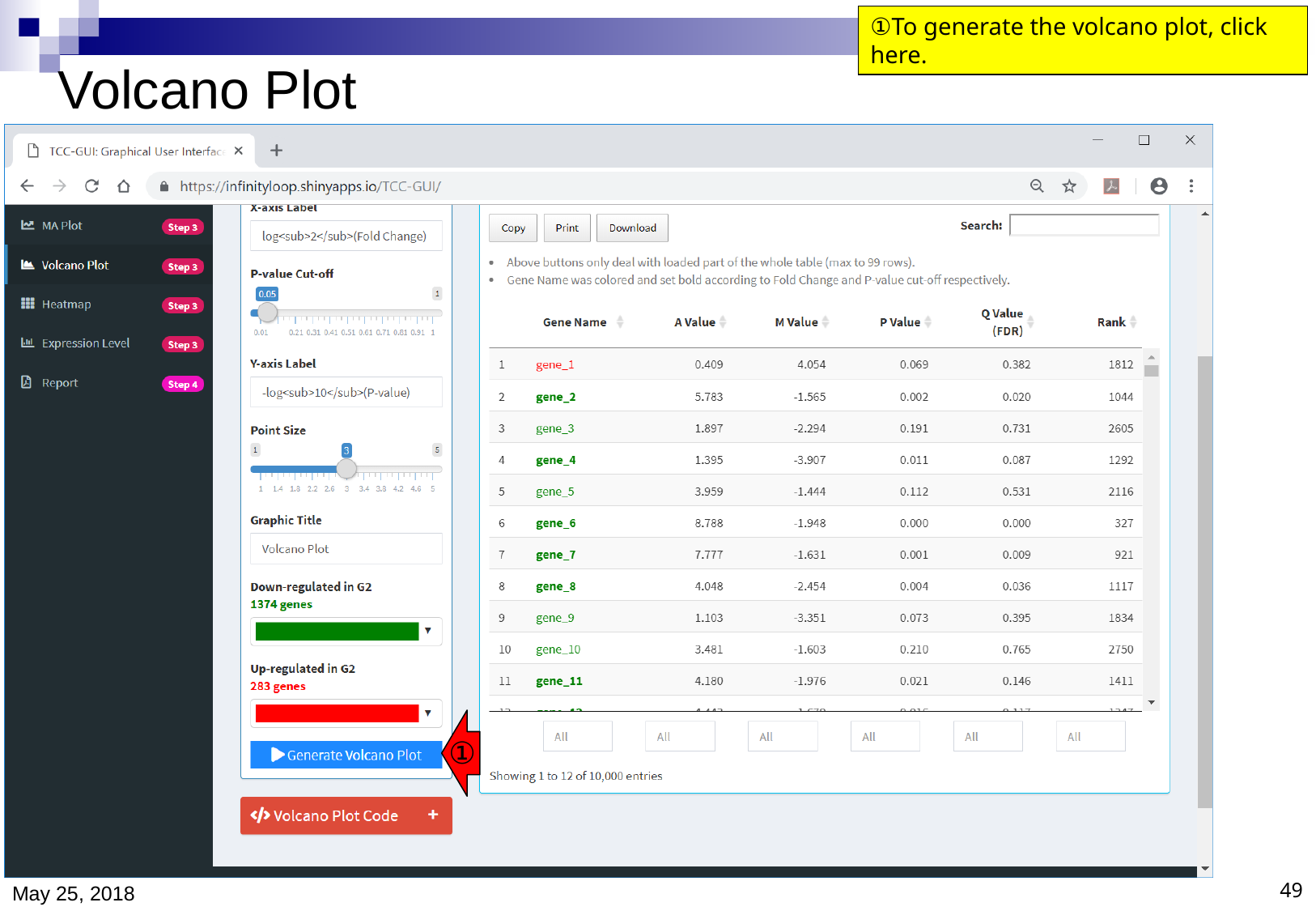

①To generate the volcano plot, click here.
# Volcano Plot
①
May 25, 2018
49

## Slide 50
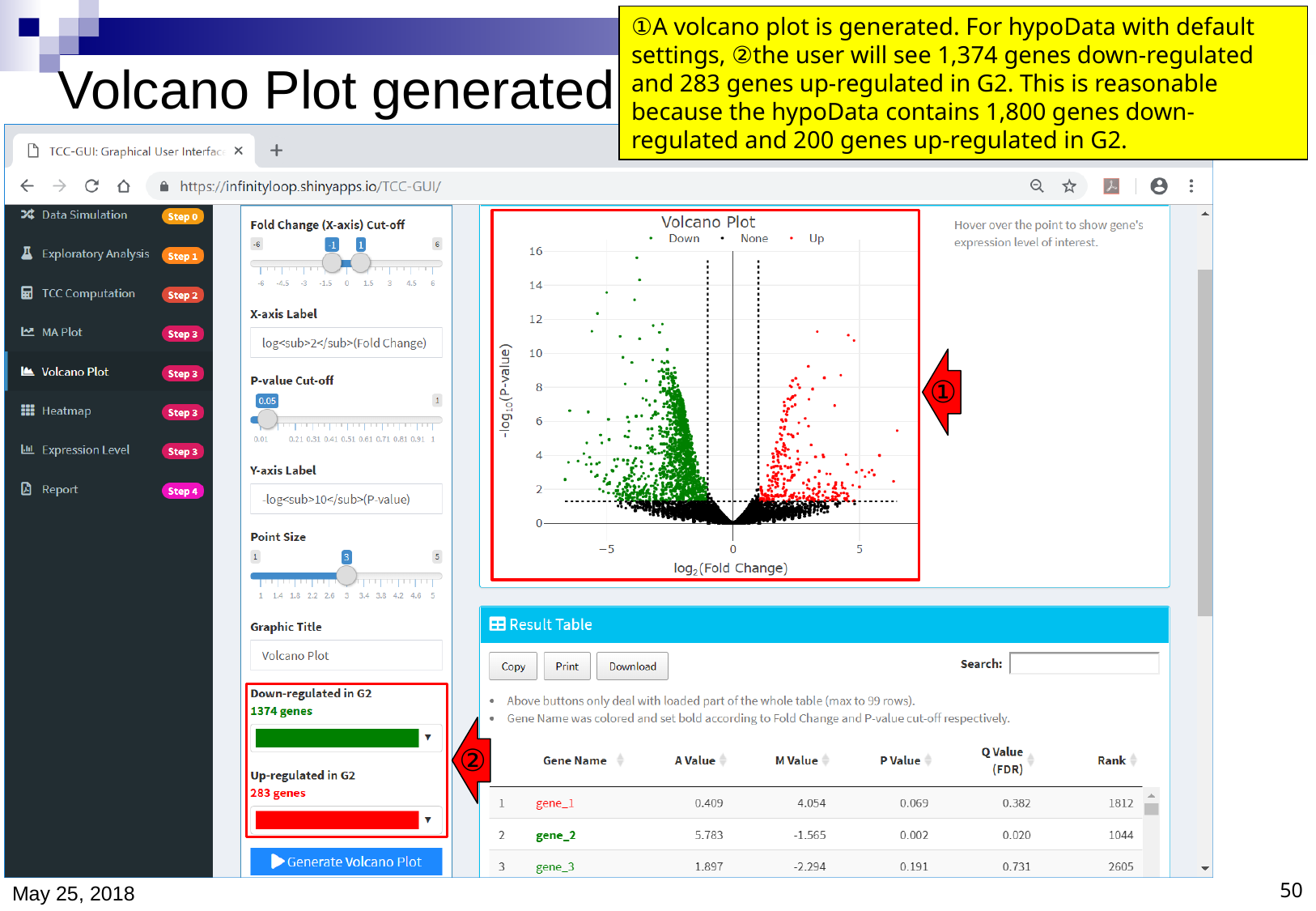

①A volcano plot is generated. For hypoData with default settings, ②the user will see 1,374 genes down-regulated and 283 genes up-regulated in G2. This is reasonable because the hypoData contains 1,800 genes down-regulated and 200 genes up-regulated in G2.
# Volcano Plot generated
①
②
May 25, 2018
50

## Slide 51
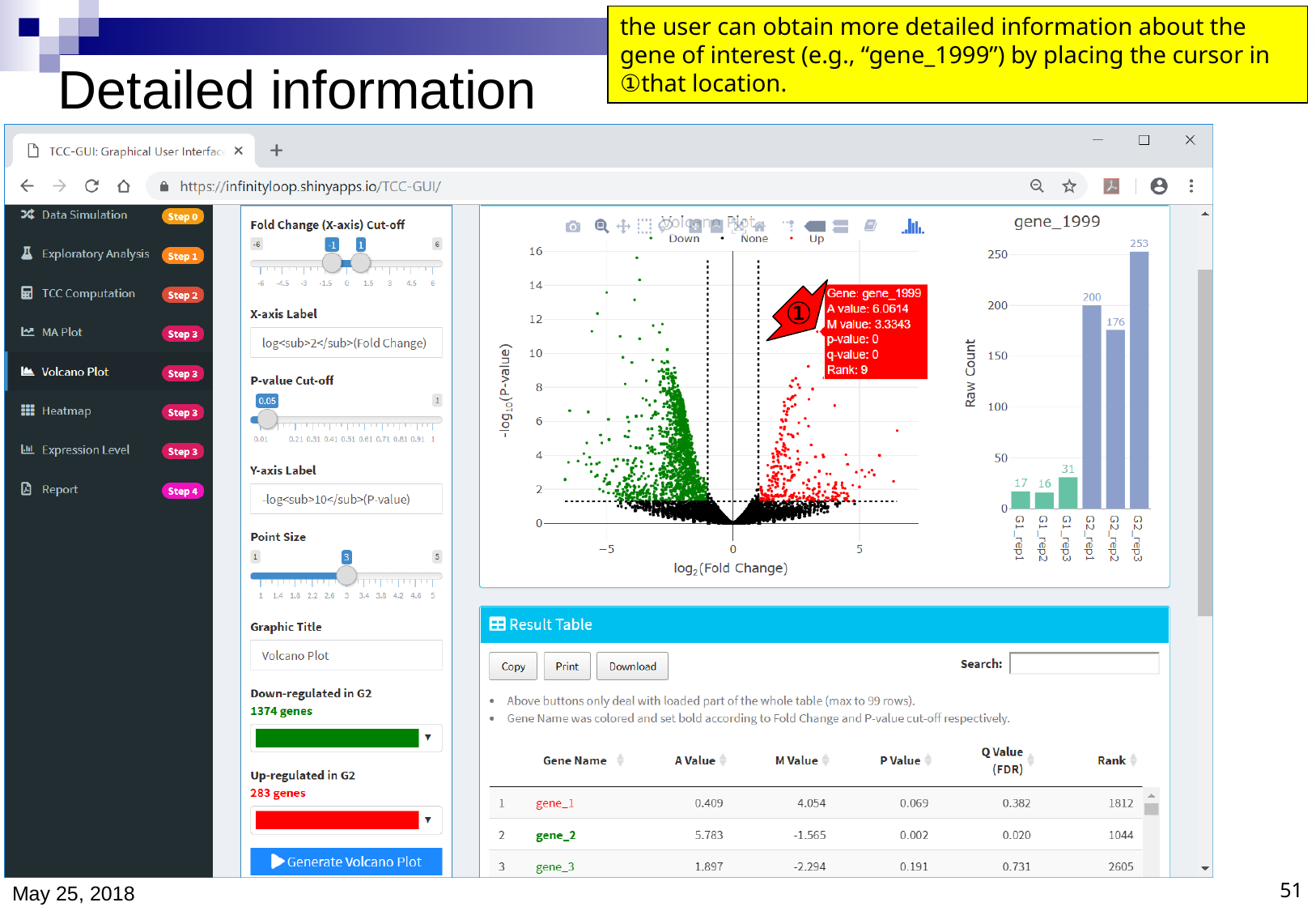

the user can obtain more detailed information about the gene of interest (e.g., “gene_1999”) by placing the cursor in ①that location.
# Detailed information
①
May 25, 2018
51

## Slide 52
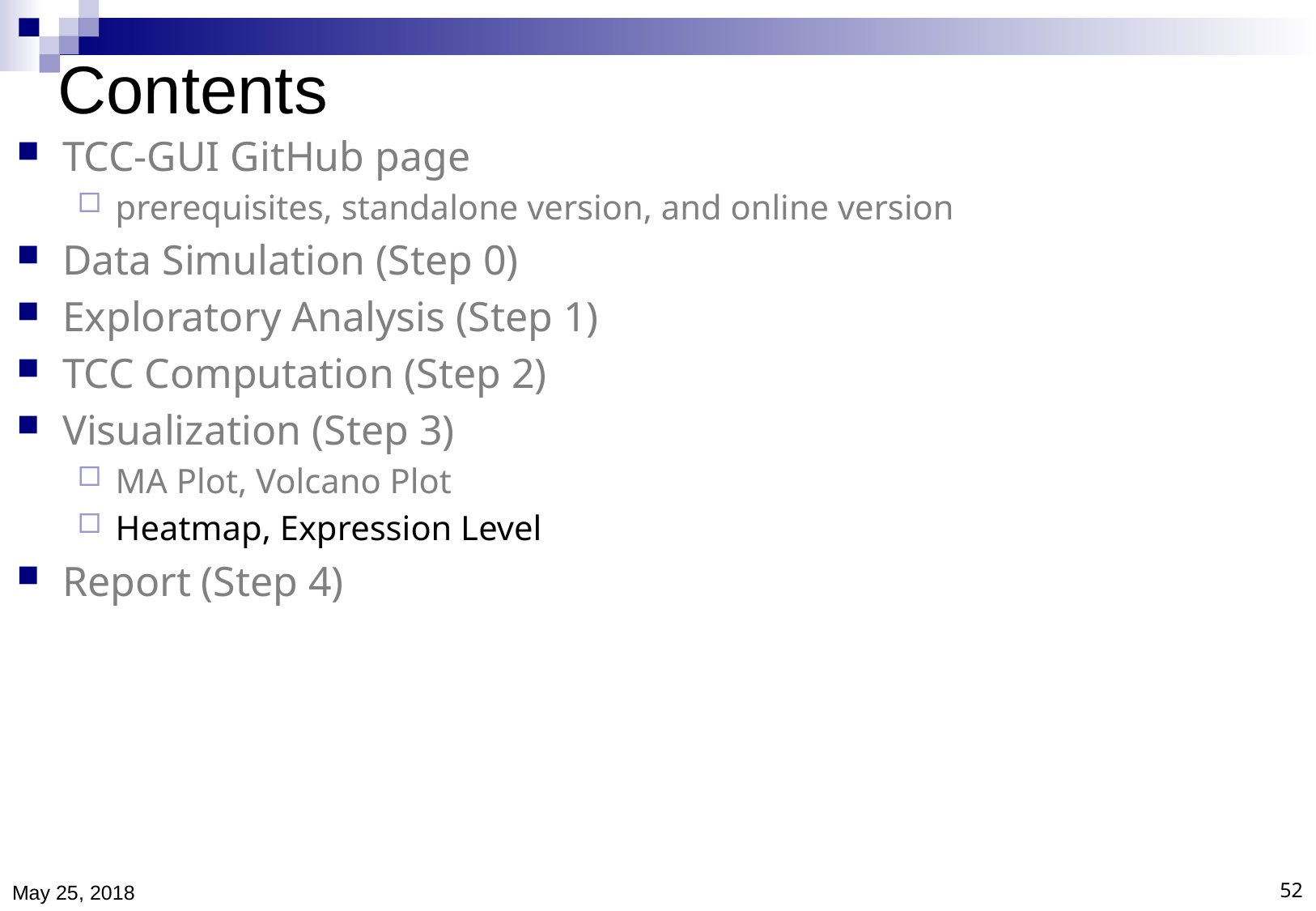

# Contents
TCC-GUI GitHub page
prerequisites, standalone version, and online version
Data Simulation (Step 0)
Exploratory Analysis (Step 1)
TCC Computation (Step 2)
Visualization (Step 3)
MA Plot, Volcano Plot
Heatmap, Expression Level
Report (Step 4)
May 25, 2018
52

## Slide 53
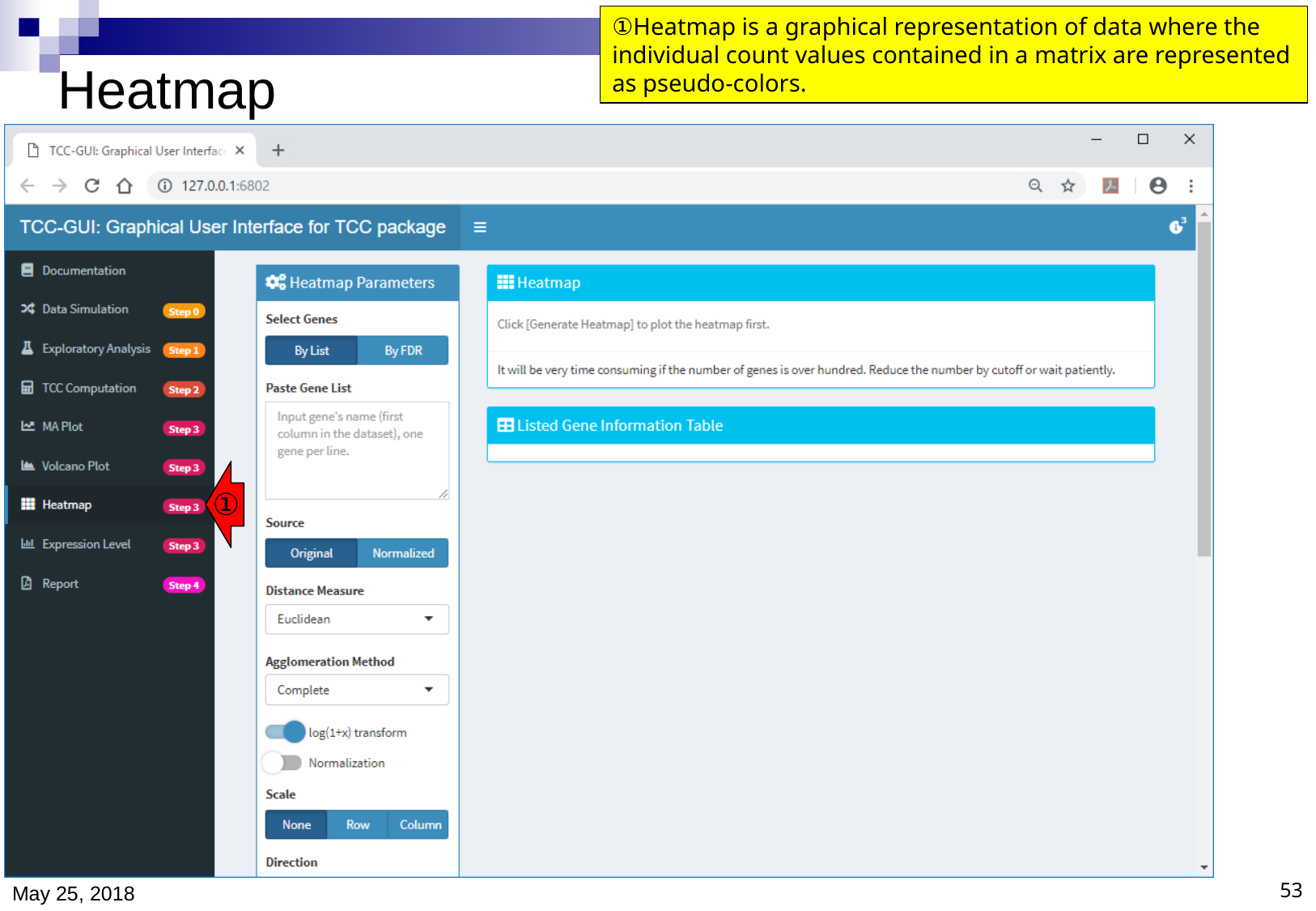

①Heatmap is a graphical representation of data where the individual count values contained in a matrix are represented as pseudo-colors.
# Heatmap
①
May 25, 2018
53

## Slide 54
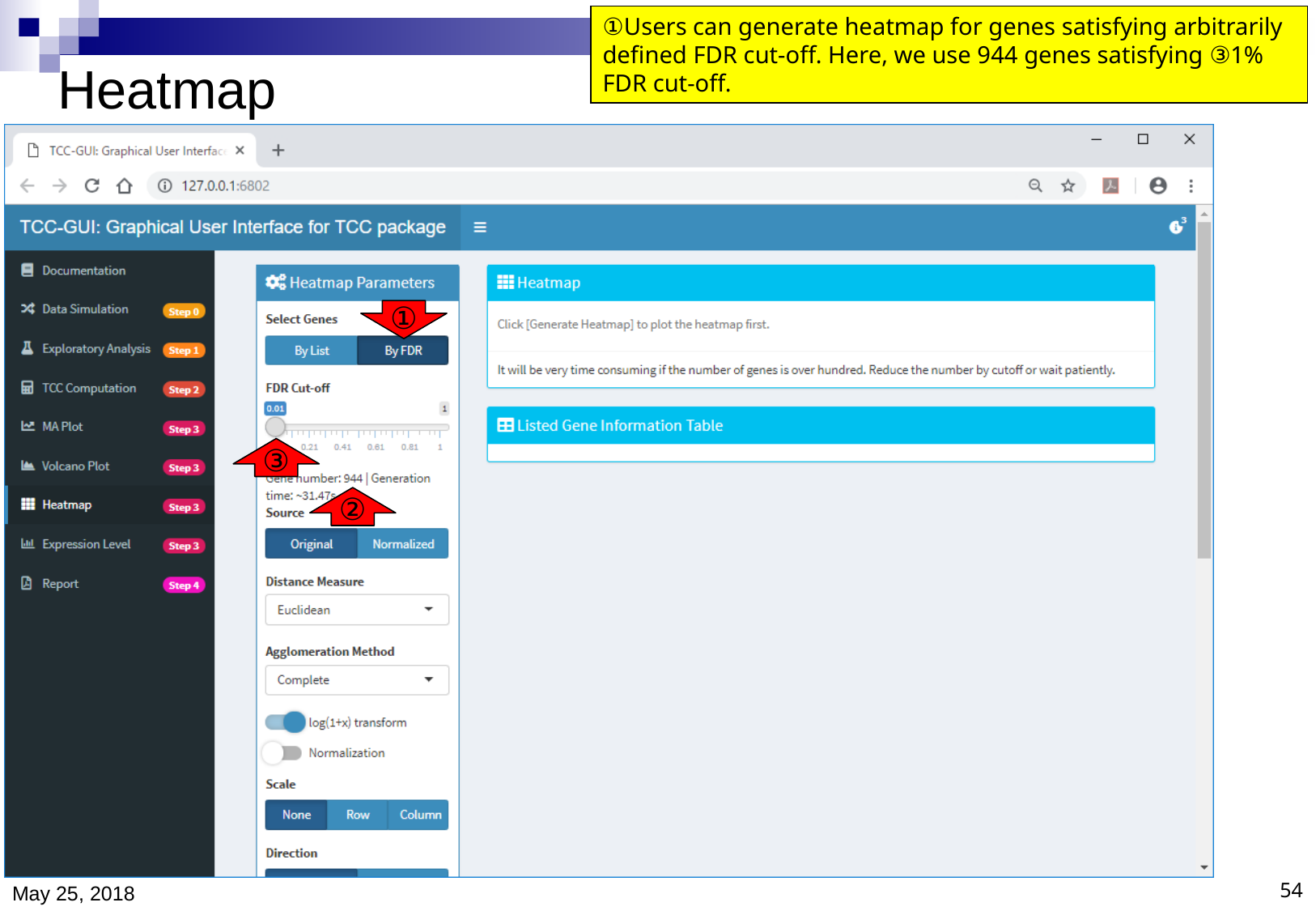

①Users can generate heatmap for genes satisfying arbitrarily defined FDR cut-off. Here, we use 944 genes satisfying ③1% FDR cut-off.
# Heatmap
①
③
②
May 25, 2018
54

## Slide 55
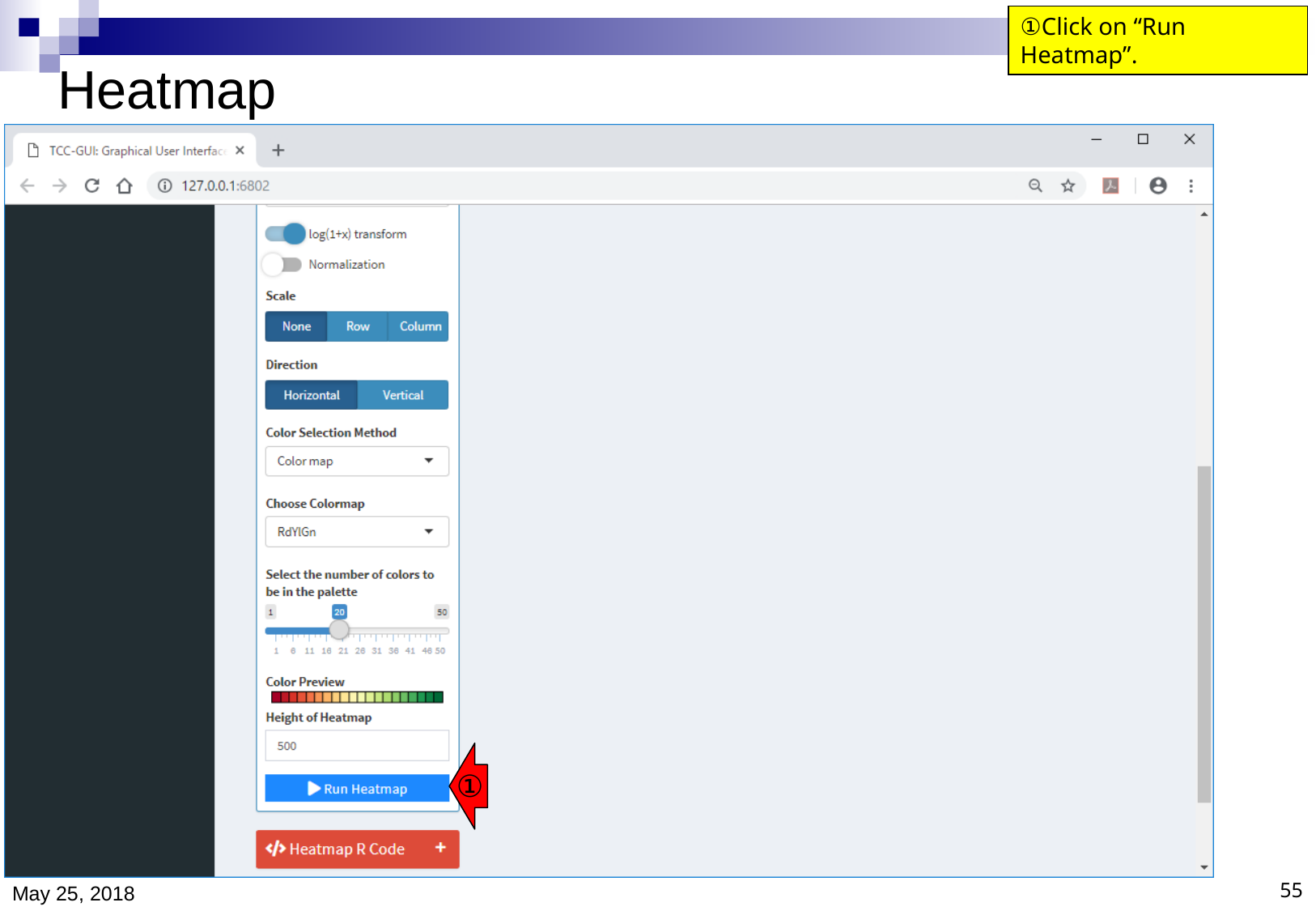

①Click on “Run Heatmap”.
# Heatmap
①
May 25, 2018
55

## Slide 56
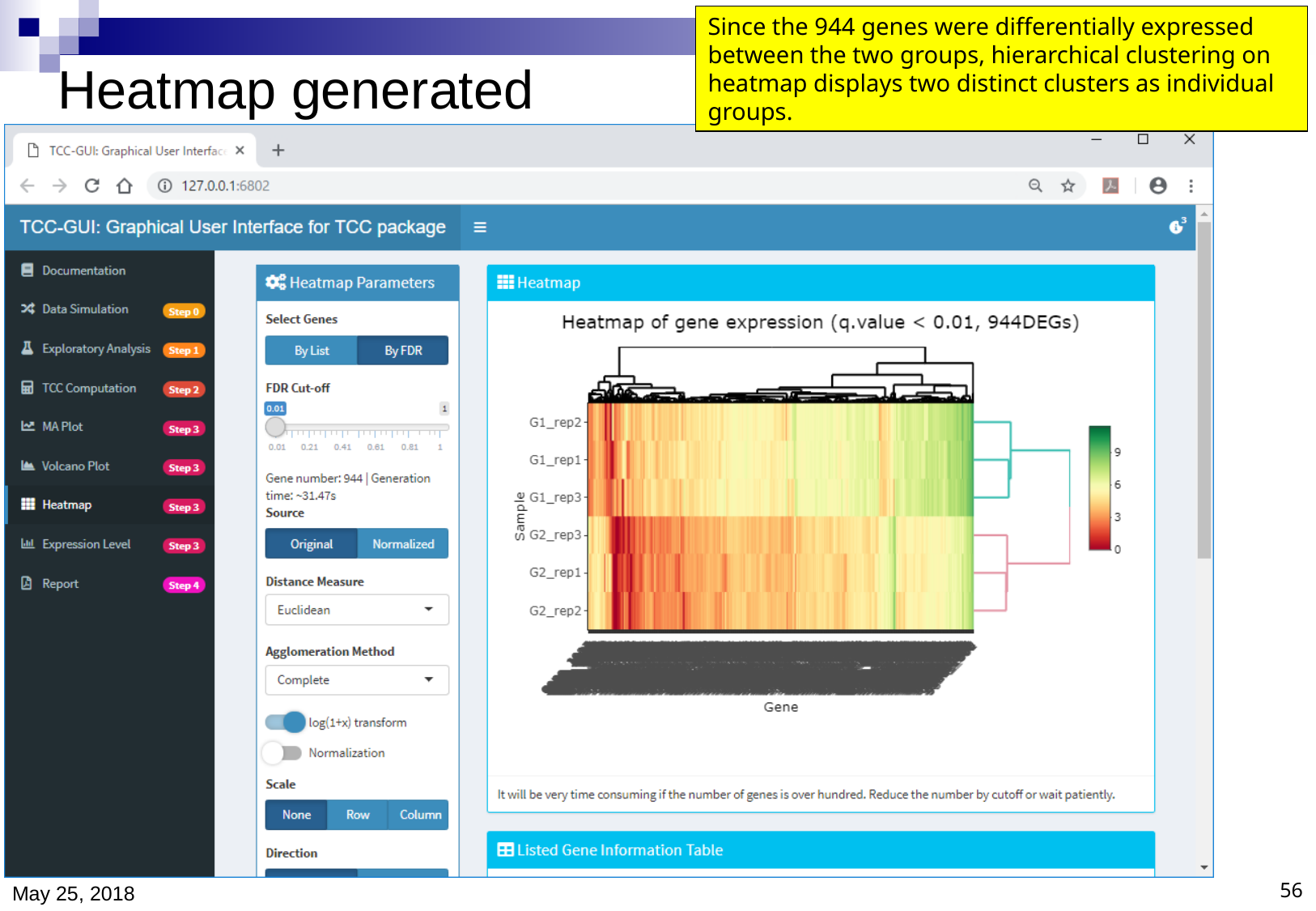

Since the 944 genes were differentially expressed between the two groups, hierarchical clustering on heatmap displays two distinct clusters as individual groups.
# Heatmap generated
May 25, 2018
56

## Slide 57
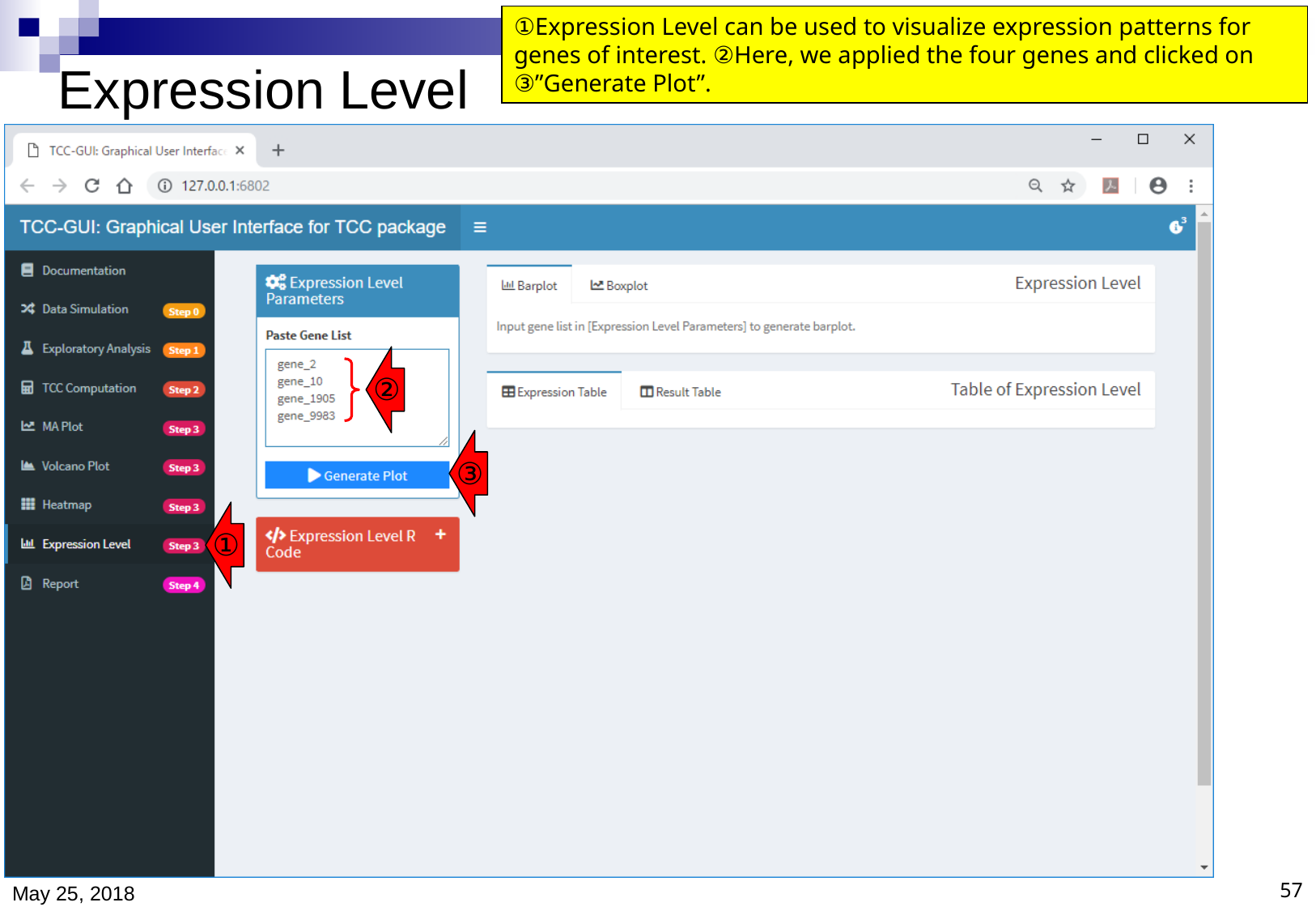

①Expression Level can be used to visualize expression patterns for genes of interest. ②Here, we applied the four genes and clicked on ③”Generate Plot”.
# Expression Level
②
③
①
May 25, 2018
57

## Slide 58
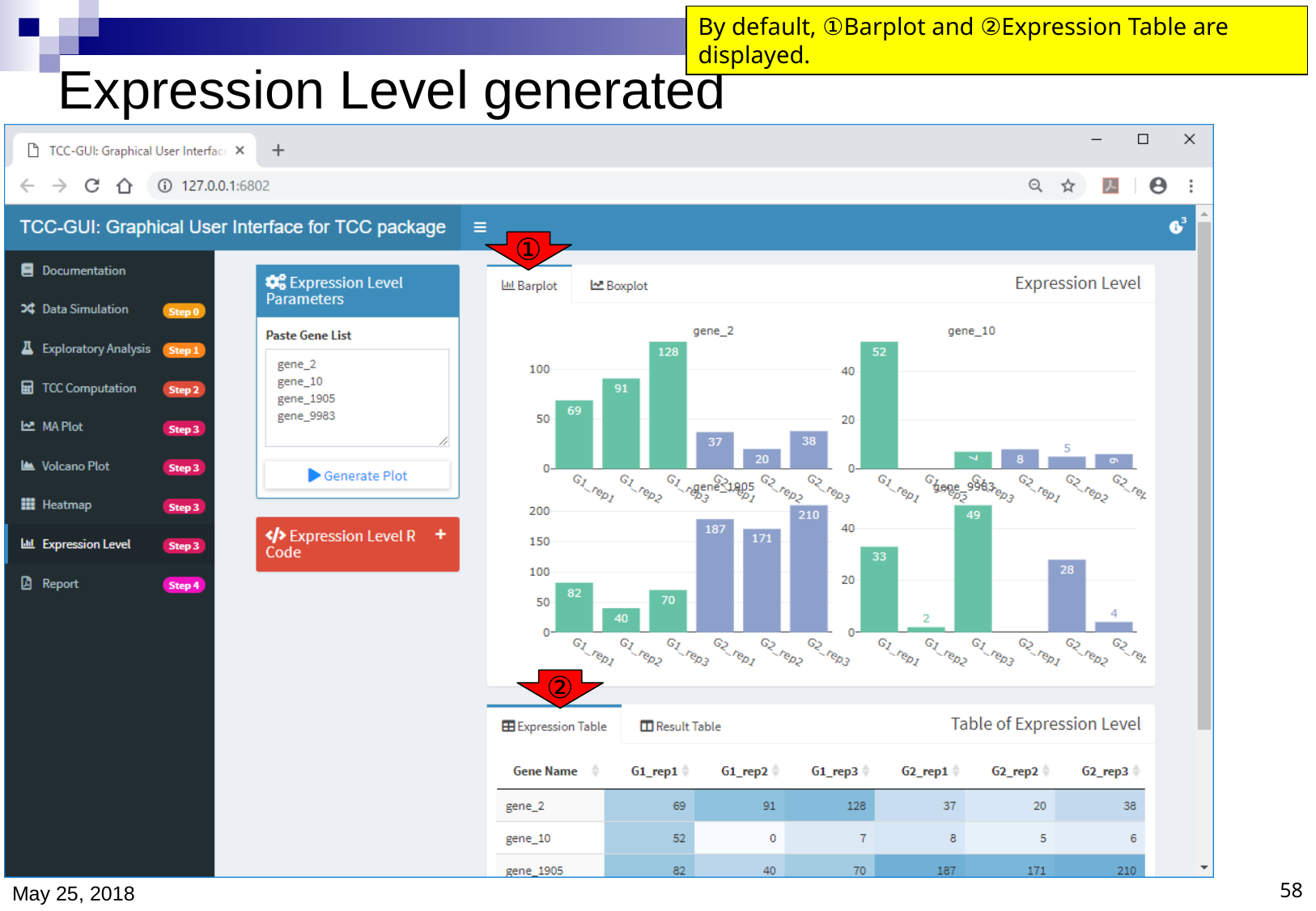

By default, ①Barplot and ②Expression Table are displayed.
# Expression Level generated
①
②
May 25, 2018
58

## Slide 59
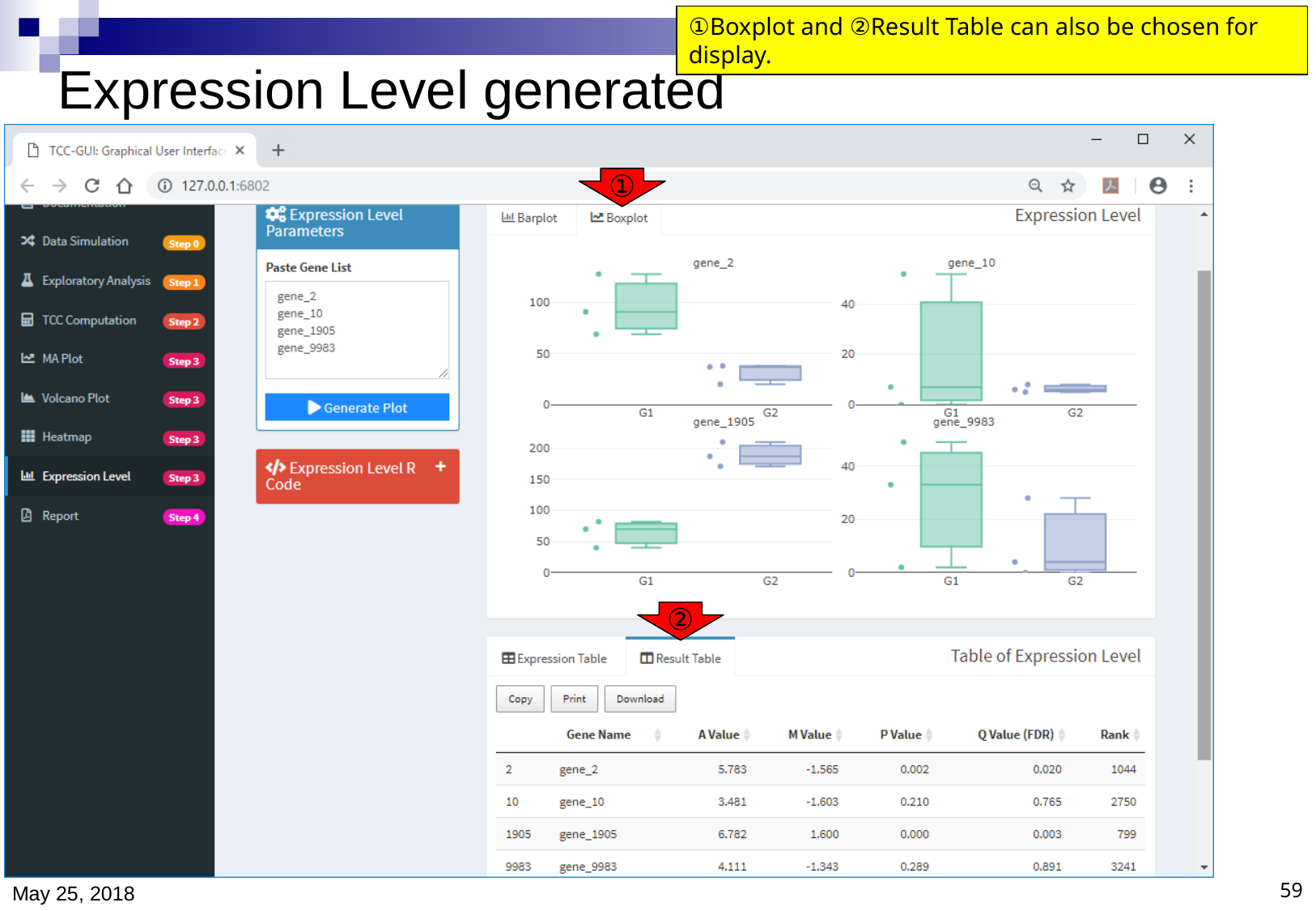

①Boxplot and ②Result Table can also be chosen for display.
# Expression Level generated
①
②
May 25, 2018
59

## Slide 60
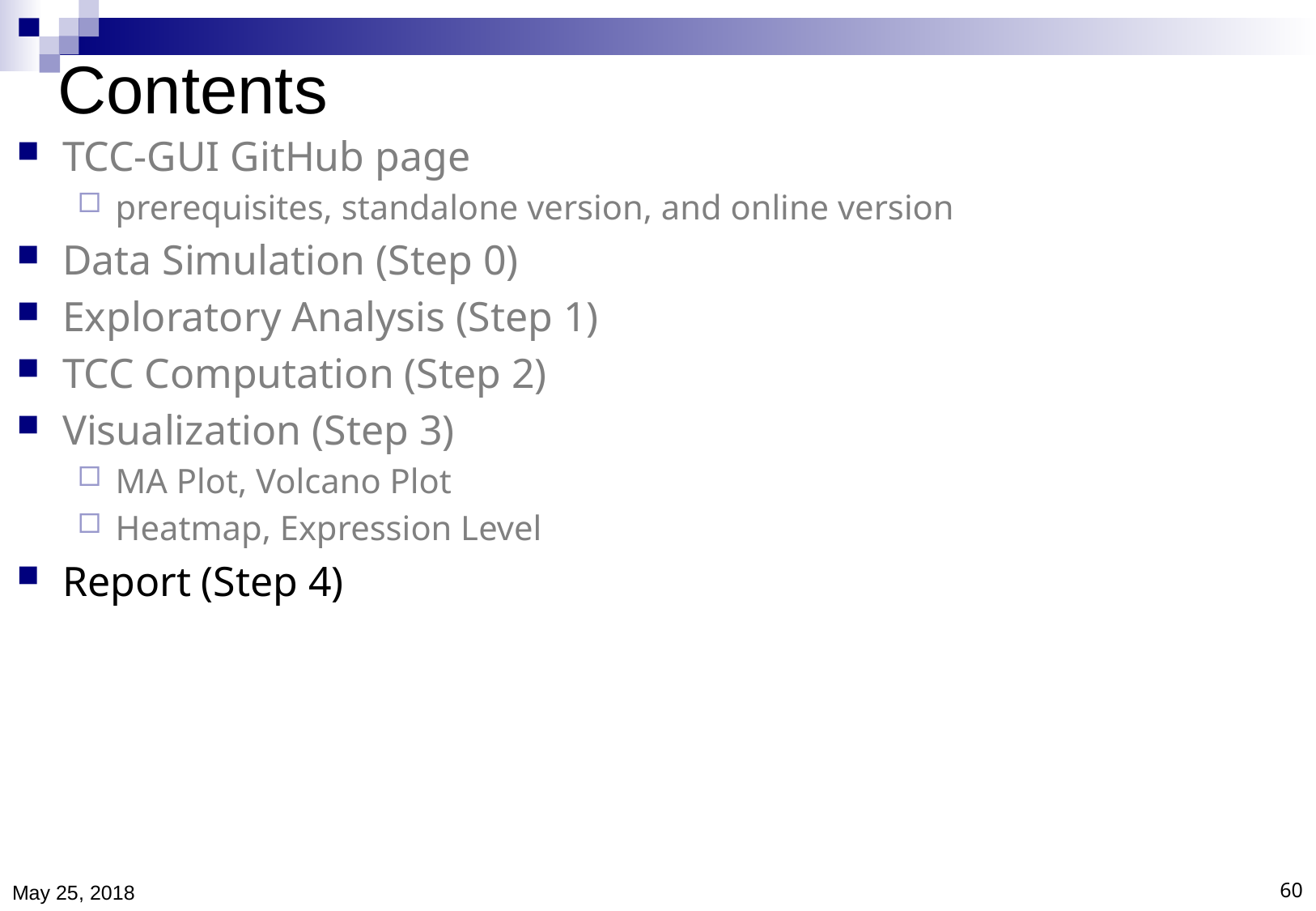

# Contents
TCC-GUI GitHub page
prerequisites, standalone version, and online version
Data Simulation (Step 0)
Exploratory Analysis (Step 1)
TCC Computation (Step 2)
Visualization (Step 3)
MA Plot, Volcano Plot
Heatmap, Expression Level
Report (Step 4)
May 25, 2018
60

## Slide 61
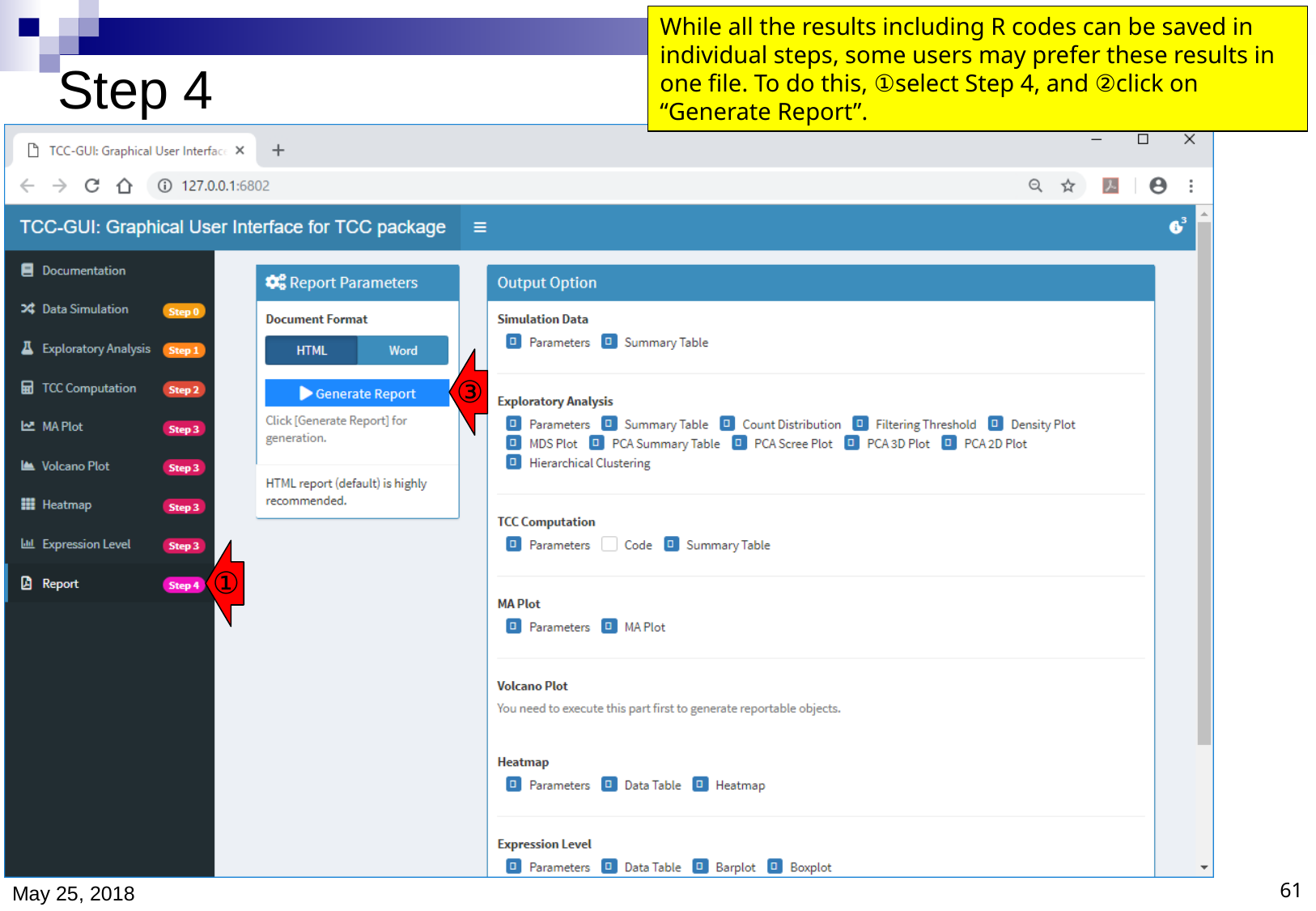

While all the results including R codes can be saved in individual steps, some users may prefer these results in one file. To do this, ①select Step 4, and ②click on “Generate Report”.
# Step 4
③
①
May 25, 2018
61

## Slide 62
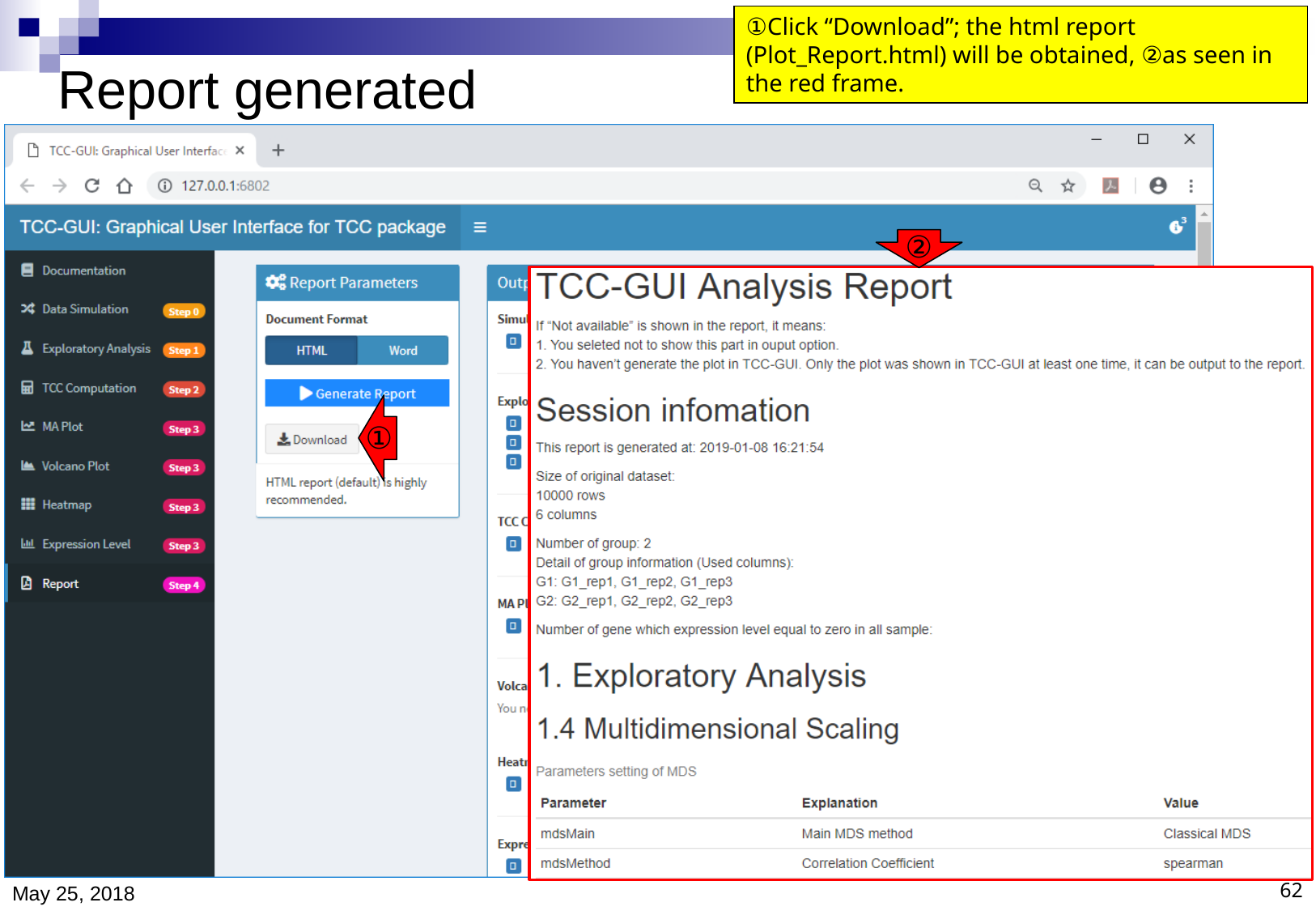

①Click “Download”; the html report (Plot_Report.html) will be obtained, ②as seen in the red frame.
# Report generated
②
①
May 25, 2018
62
