# Supplementary material for: TCC-GUI: a Shiny-based application for differential expression analysis of RNA-Seq count data
Source: BMC Res Notes. 2019 Mar 13;12:133. doi: 10.1186/s13104-019-4179-2 (PMC6417217; doi:10.1186/s13104-019-4179-2)
Supplement: Supplementary file 3 — Additional file 3. Representative analysis of Bottomly’s dataset. A series of screenshots while analyzing Bottomly’s real count dataset is provided. [file 13104_2019_4179_MOESM3_ESM.pptx]

## Slide 1
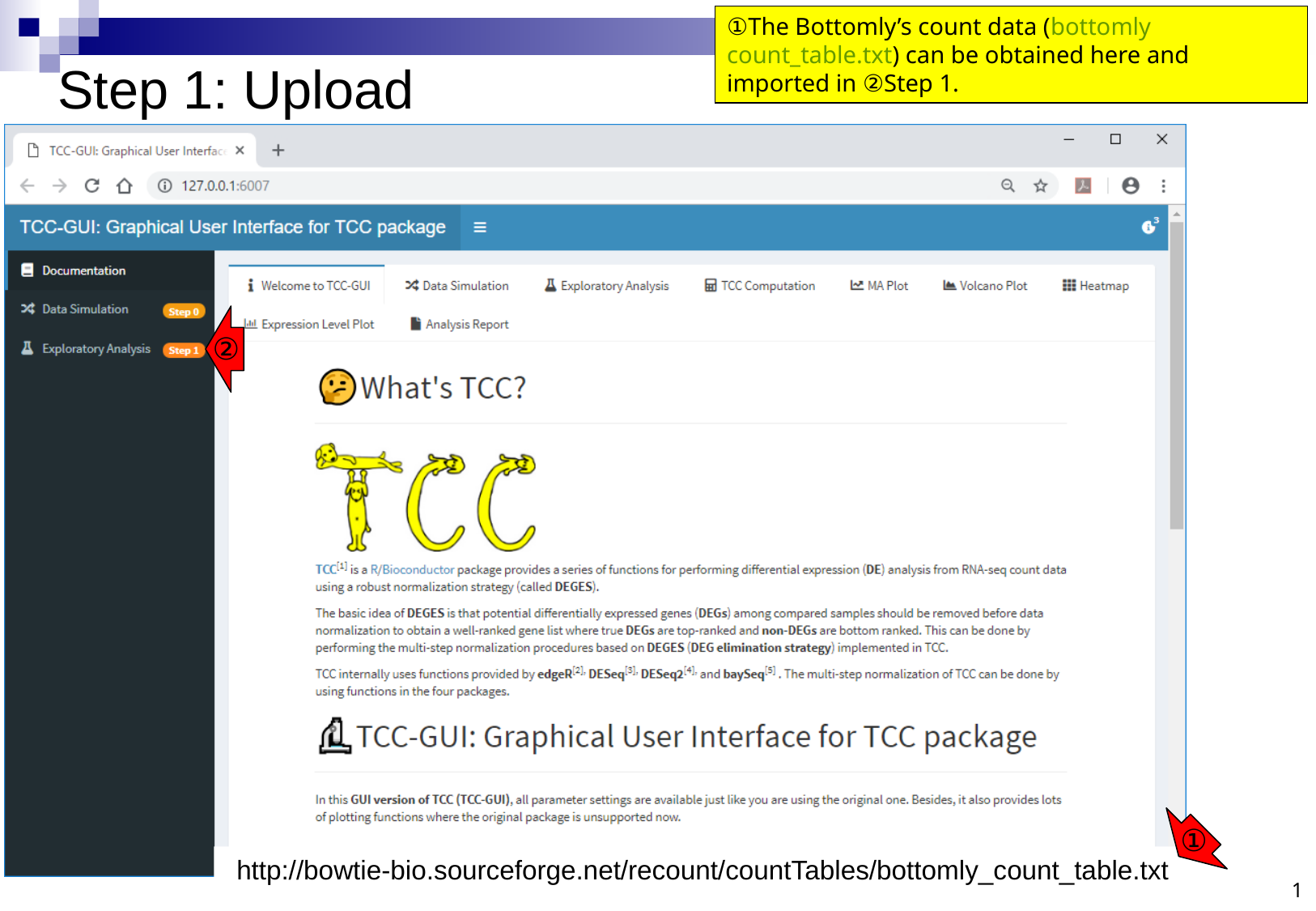

①The Bottomly’s count data (bottomly count_table.txt) can be obtained here and imported in ②Step 1.
# Step 1: Upload
②
①
http://bowtie-bio.sourceforge.net/recount/countTables/bottomly_count_table.txt
1

## Slide 2
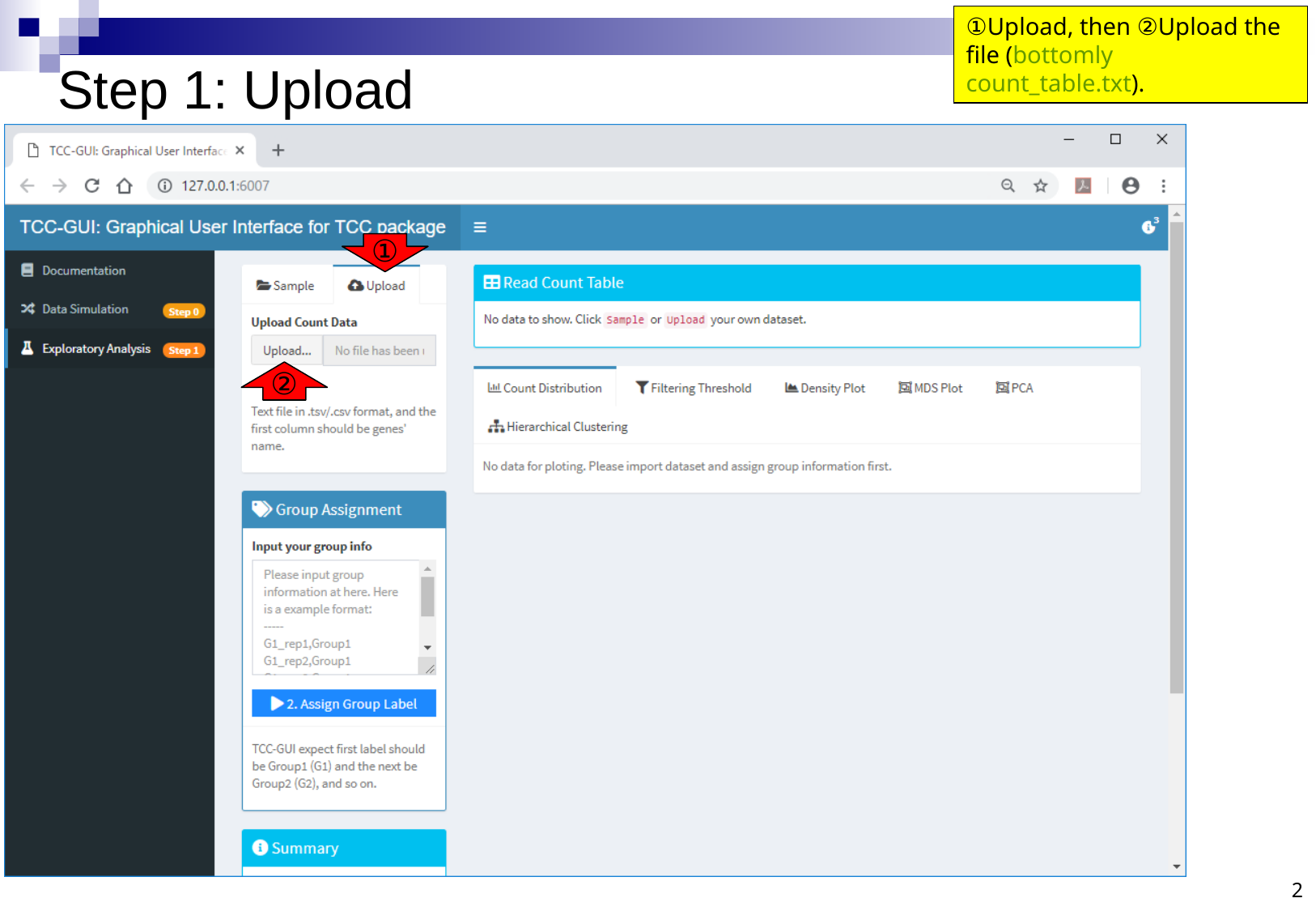

①Upload, then ②Upload the file (bottomly count_table.txt).
# Step 1: Upload
①
②
2

## Slide 3
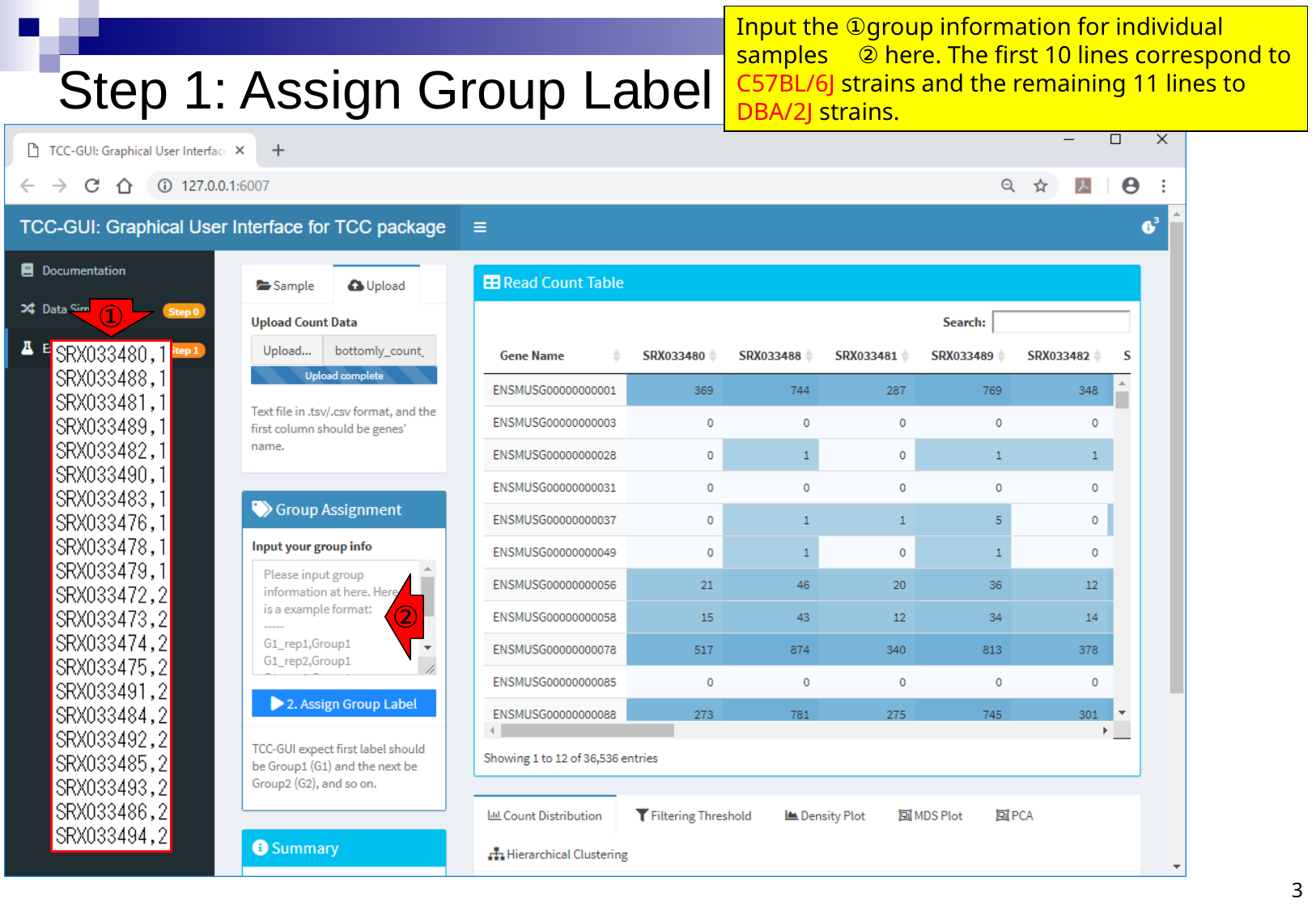

Input the ①group information for individual samples　②here. The first 10 lines correspond to C57BL/6J strains and the remaining 11 lines to DBA/2J strains.
# Step 1: Assign Group Label
①
②
3

## Slide 4
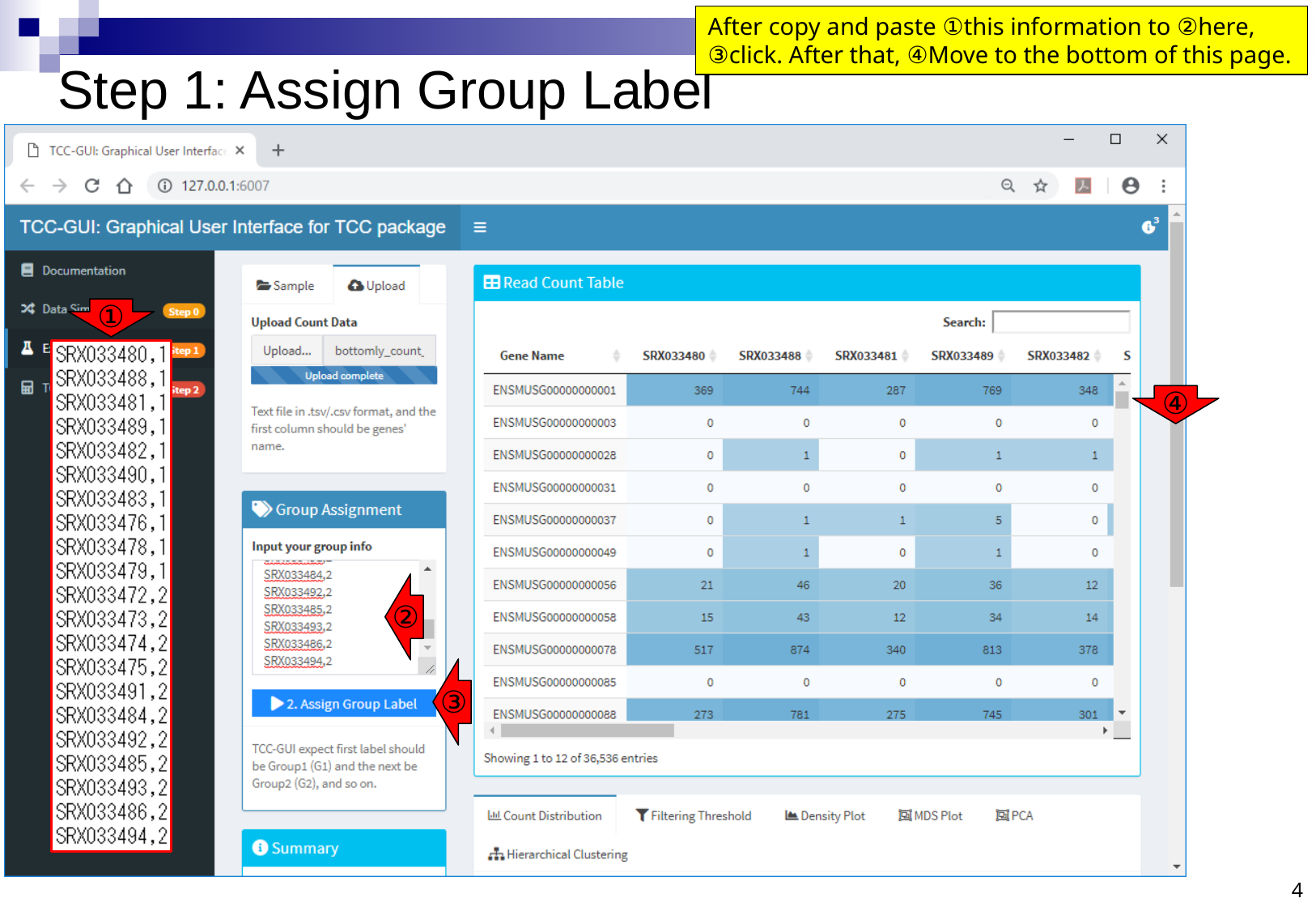

After copy and paste ①this information to ②here, ③click. After that, ④Move to the bottom of this page.
# Step 1: Assign Group Label
①
④
②
③
4

## Slide 5
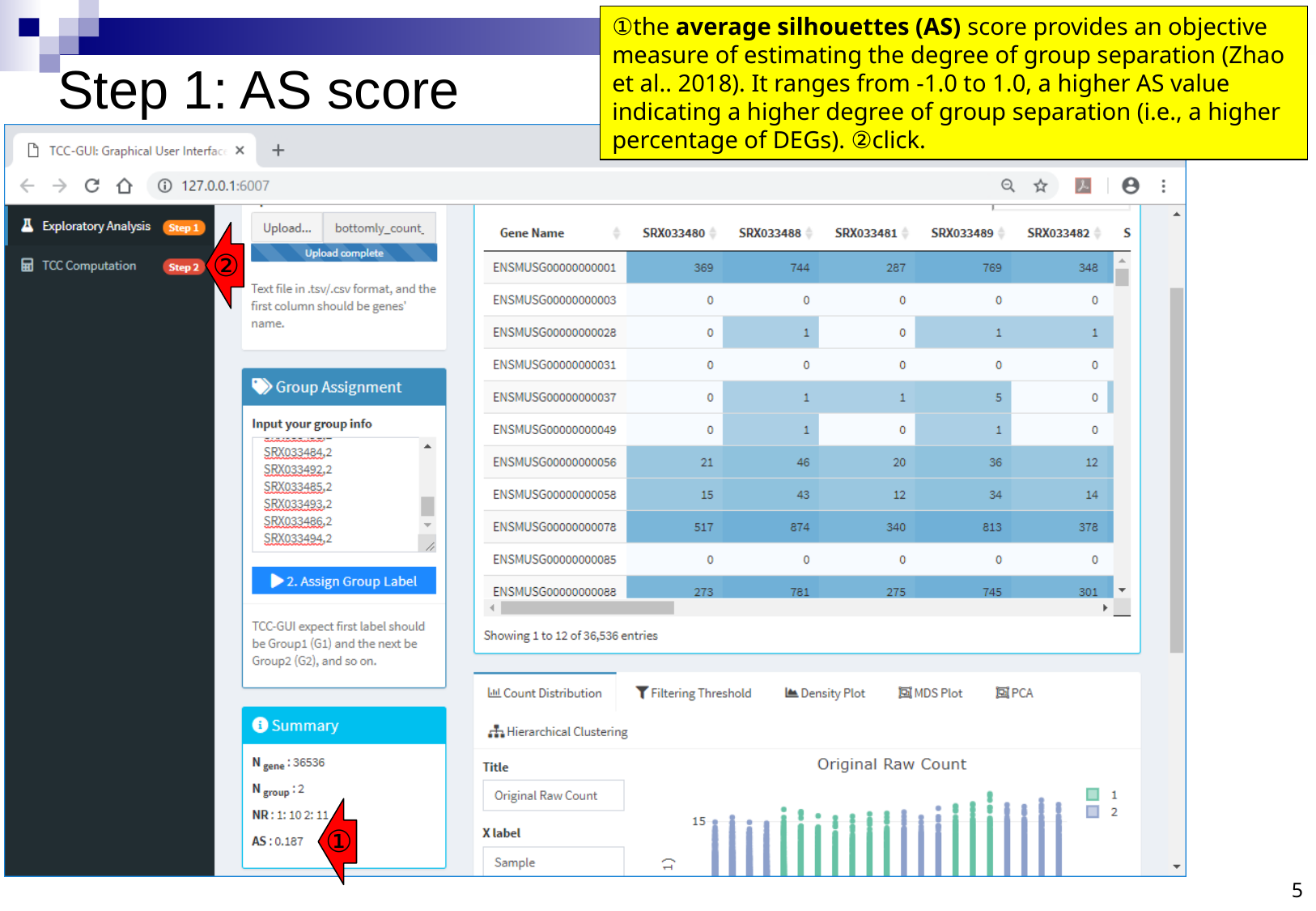

①the average silhouettes (AS) score provides an objective measure of estimating the degree of group separation (Zhao et al.. 2018). It ranges from -1.0 to 1.0, a higher AS value indicating a higher degree of group separation (i.e., a higher percentage of DEGs). ②click.
# Step 1: AS score
②
①
5

## Slide 6
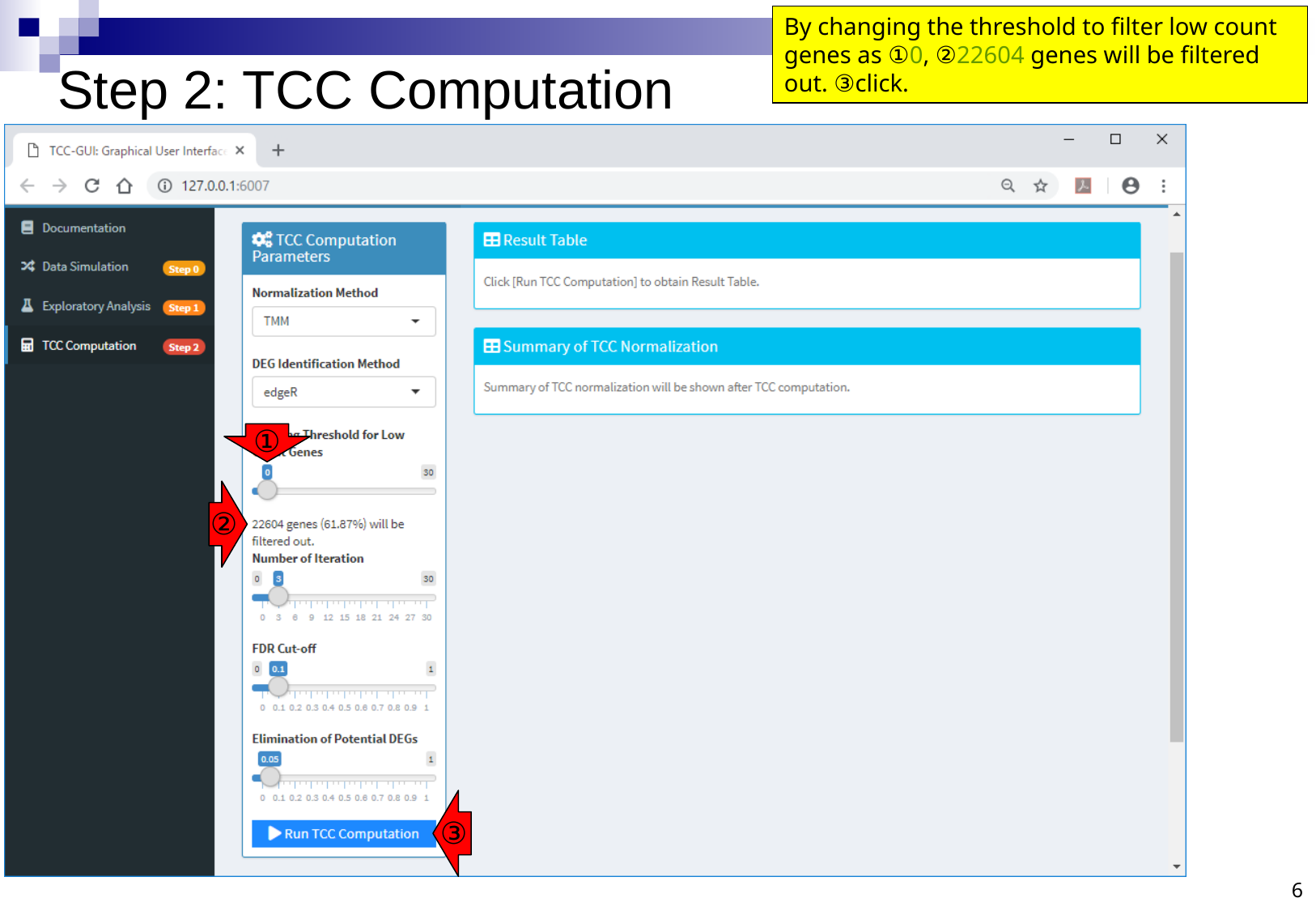

By changing the threshold to filter low count genes as ①0, ②22604 genes will be filtered out. ③click.
# Step 2: TCC Computation
①
②
③
6

## Slide 7
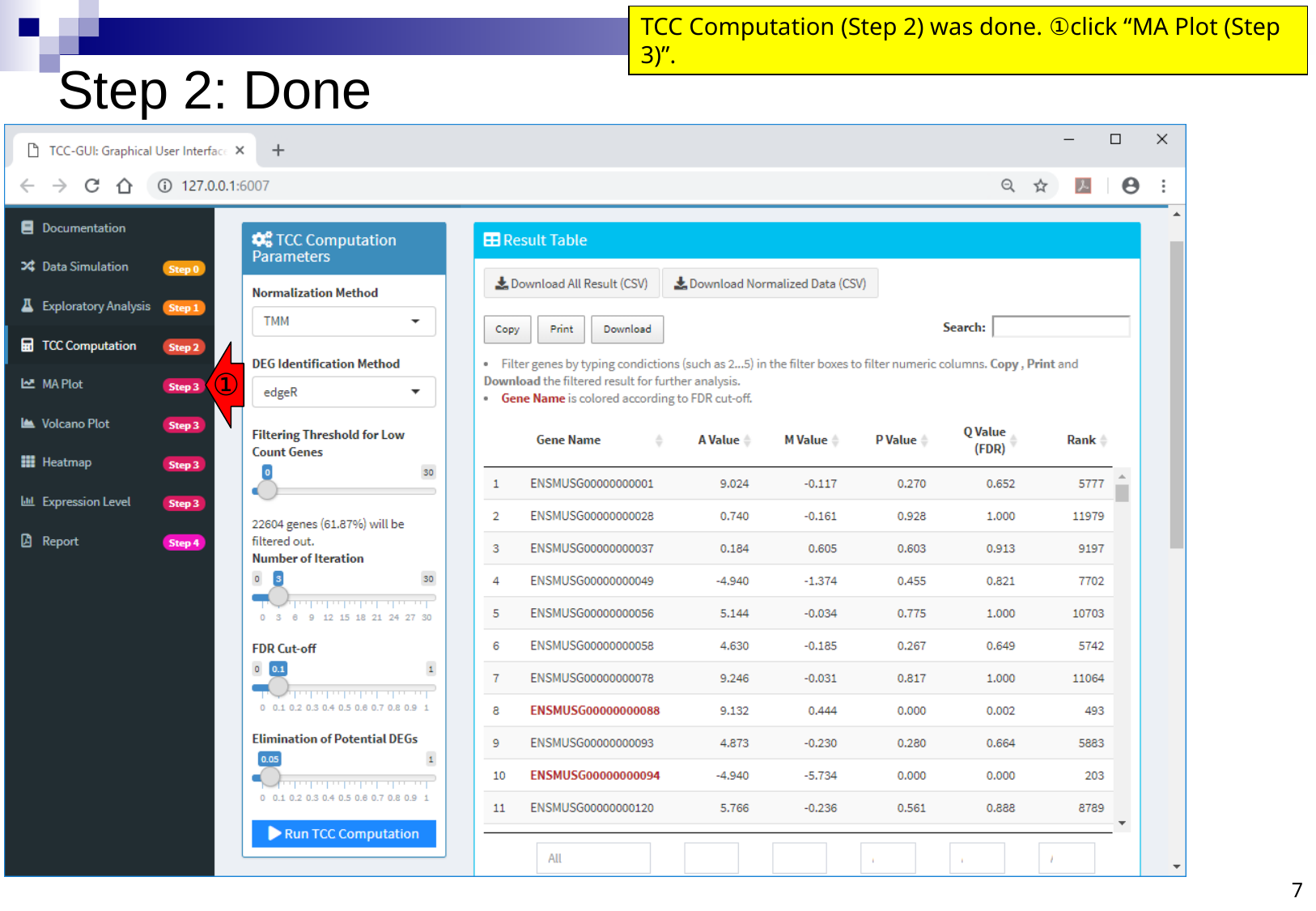

TCC Computation (Step 2) was done. ①click “MA Plot (Step 3)”.
# Step 2: Done
①
7

## Slide 8
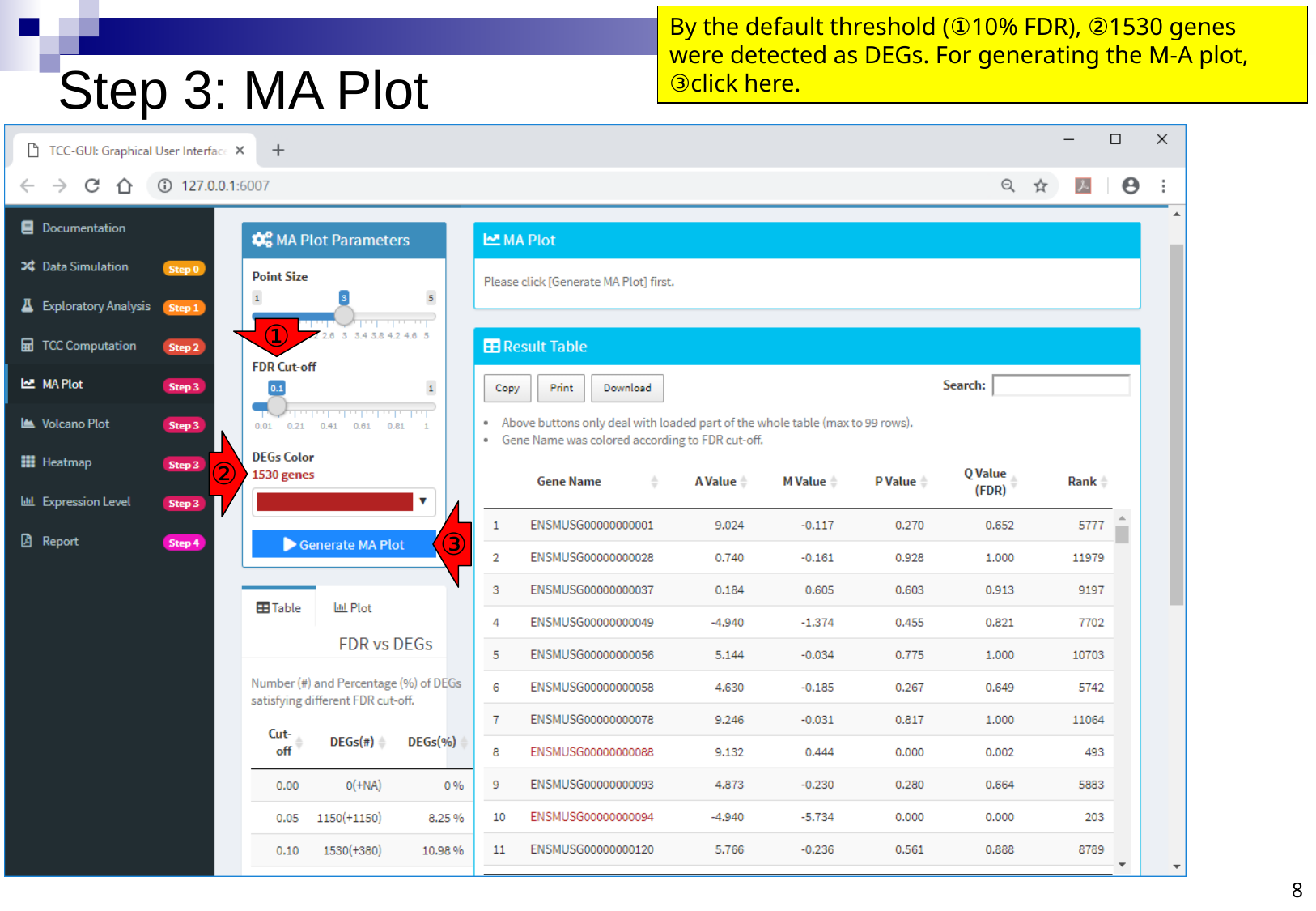

By the default threshold (①10% FDR), ②1530 genes were detected as DEGs. For generating the M-A plot, ③click here.
# Step 3: MA Plot
①
②
③
8

## Slide 9
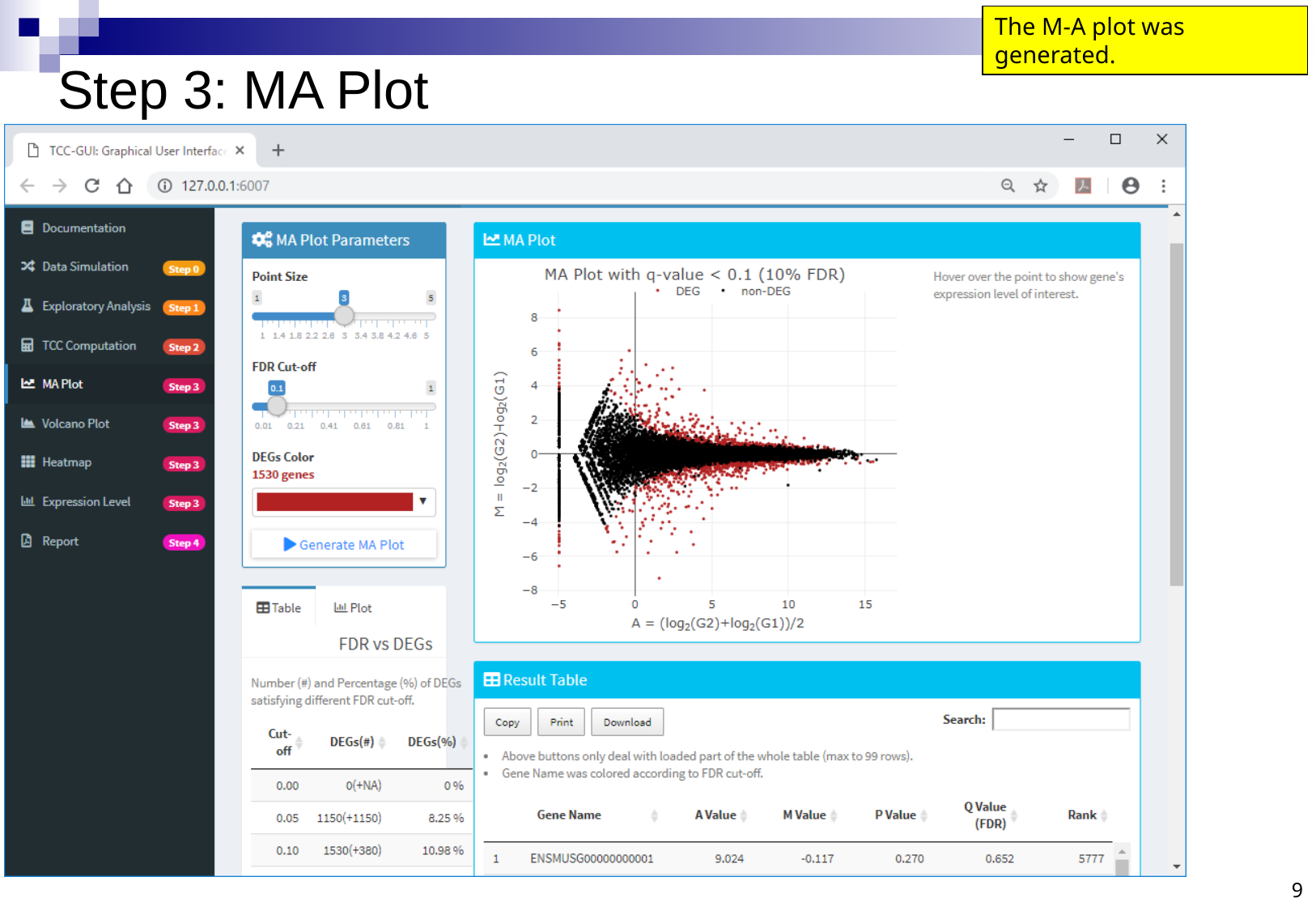

The M-A plot was generated.
# Step 3: MA Plot
9
